# Identifying Serum Metabolomic Markers Associated with Skin Disease Activity in Patients with Psoriatic Arthritis

**DOI:** 10.3390/ijms242015299

**Published:** 2023-10-18

**Authors:** Hani Choksi, Shenghan Li, Nikita Looby, Max Kotlyar, Igor Jurisica, Vathany Kulasingam, Vinod Chandran

**Affiliations:** 1 Schroeder Arthritis Program, Krembil Research Institute, University Health Network, Toronto, ON M5T 0S8, Canada; hani.choksi@utoronto.ca (H.C.); hanhan.li@mail.utoronto.ca (S.L.); nikita.looby@uhn.ca (N.L.); 2 Department of Laboratory Medicine and Pathobiology, University of Toronto, Toronto, ON M5S 1A1, Canada; 3 Osteoarthritis Research Program, Division of Orthopedic Surgery, Schroeder Arthritis Institute and Data Science Discovery Centre for Chronic Diseases, Krembil Research Institute, Toronto, ON M5T 0S8, Canada; max.kotlyar@uhn.ca (M.K.); juris@ai.utoronto.ca (I.J.); 4 Departments of Medical Biophysics and Computer Science, and Faculty of Dentistry, University of Toronto, Toronto, ON M5S 1A1, Canada; 5 Institute of Neuroimmunology, Slovak Academy of Sciences, Dubravská cesta 9, 845 10 Bratislava, Slovakia; 6 Division of Clinical Biochemistry, Laboratory Medicine Program, University Health Network, Toronto, ON M5T 0S8, Canada; 7 Division of Rheumatology, Department of Medicine, University of Toronto, Toronto, ON M5S 1A8, Canada

**Keywords:** metabolomics, solid phase microextraction, liquid chromatography, mass spectrometry, psoriatic arthritis, psoriasis area severity index, machine learning

## Abstract

Psoriatic arthritis (PsA) is a chronic, systemic, immune-mediated inflammatory disease causing cutaneous and musculoskeletal inflammation that affects 25% of patients with psoriasis. Current methods for evaluating PsA disease activity are not accurate enough for precision medicine. A metabolomics-based approach can elucidate psoriatic disease pathogenesis, providing potential objective biomarkers. With the hypothesis that serum metabolites are associated with skin disease activity, we aimed to identify serum metabolites associated with skin activity in PsA patients. We obtained serum samples from patients with PsA (n = 150) who were classified into mild, moderate and high disease activity groups based on the Psoriasis Area Severity Index. We used solid-phase microextraction (SPME) for sample preparation, followed by data acquisition via an untargeted liquid chromatography—mass spectrometry (LC-MS) approach. Disease activity levels were predicted using identified metabolites and machine learning algorithms. Some metabolites tentatively identified include eicosanoids with anti- or pro-inflammatory properties, like 12-Hydroxyeicosatetraenoic acid, which was previously implicated in joint disease activity in PsA. Other metabolites of interest were associated with dysregulation of fatty acid metabolism and belonged to classes such as bile acids, oxidized phospholipids, and long-chain fatty acids. We have identified potential metabolites associated with skin disease activity in PsA patients.

## 1. Introduction

Psoriasis is a chronic, immune-mediated, systemic inflammatory disease that affects over 2.5% of the global population, including 1.25 million Canadians [1,2]. It commonly manifests as chronic plaque psoriasis, where red, itchy scaly plaques develop on the scalp, elbows, and other parts of the body [2]. Approximately 25% of psoriasis patients also carry the additional burden of inflammatory arthritis and have psoriatic arthritis (PsA) [3]. Patients with PsA have cutaneous and musculoskeletal inflammation [2,4]. The progressive joint damage, stiffness, pain, and disability associated with PsA is responsible for numerous other comorbidities, especially those associated with high risk for cardiovascular diseases [5,6], including diabetes [7,8], obesity [9,10], and lipidemia [11,12]. This leads to a reduced overall quality of life and early mortality for these patients. While their precise etiologies are unknown, psoriasis and psoriatic arthritis have complex pathogenesis involving environmental, immunologic, and genetic factors [13]. 

Measuring and monitoring disease activity is critical to the management of PsA. Assessing PsA activity involves evaluating multiple domains including skin and musculoskeletal domains [14]. Skin lesions are usually assessed using the Psoriasis Area and Severity Index (PASI) [15]. Despite skin plaques being a prominent feature of psoriatic disease, current methods for evaluating skin disease activity, including PASI, body surface area affected, and physician global assessments, are largely subjective, imprecise and have high inter-rater variability [15,16]. As such, there is a tremendous clinical need for identifying valid, objective, and reliable biomarkers for routine clinical assessment of PsA disease activity. 

Recently, there has been growing interest in performing mass spectrometry-based metabolomics studies to discover such biomarkers and further elucidate disease pathogenesis [17]. Metabolomic analysis involves studying small molecule metabolites (<1500 Da) present in biological cells, tissues, or fluids and includes compounds with diverse chemical and physical properties like sugars, nucleotides, amino acids, and lipids to name a few [17,18]. Positioned at the intersection between the genome, transcriptome and proteome with environment, metabolomic analysis involves studying the interaction of real-life end products of multiple biological processes with environmental stimuli [17,18]. It is a powerful technique for biomarker discovery in a complex disease such as PsA that results from the interaction between multiple biological and environmental factors.

The purpose of this study was to perform comprehensive metabolomic profiling of serum samples from PsA patients with varying levels of psoriasis activity, as defined by the PASI, to identify metabolomic biomarkers associated with skin disease activity. To achieve this goal, we first used SPME to extract metabolites from 150 serum samples from PsA patients. This was followed by metabolomic data acquisition using a fatty-acid-focused high-performance liquid chromatography–mass spectrometry (HPLC-MS) methodology [19]. We subsequently identified tentative biomarkers for PsA skin disease activity via data analyses using multiple machine learning algorithms.

## 2. Results

Table 1 provides the demographic and disease characteristics of the study subjects. Most patients had long-standing psoriasis and PsA and were on treatment. 

### 2.1. Principal Component Analysis

**Figure 1:**
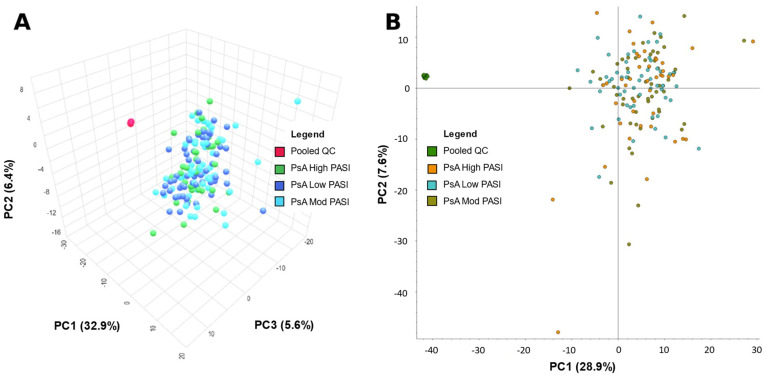
Principal component analysis (PCA) of metabolite data from psoriatic arthritis (PsA) patients from positive mode data acquisition. (**A**) PCA—PC1: 32.9%, PC2: 6.4%, PC3: 5.6% of patients with PsA, and pooled quality control (PooledQC). High-skin-disease-activity PsA, low-disease-activity PsA, moderate-disease-activity PsA, and PooledQC are represented in green, dark blue, light blue, and red, respectively. Data shown was processed via Compound Discoverer 3.3 then exported to MetaboAnalyst. (**B**) PCA—PC1: 28.9%, PC2: 7.6% of PsA patients, and pooled QCs. High-skin-disease-activity PsA, low-disease-activity PsA, moderate-disease-activity PsA, and PooledQC are represented in orange, teal, brown, and green, respectively. The data shown were obtained using Compound Discoverer 3.3.

**Figure 2:**
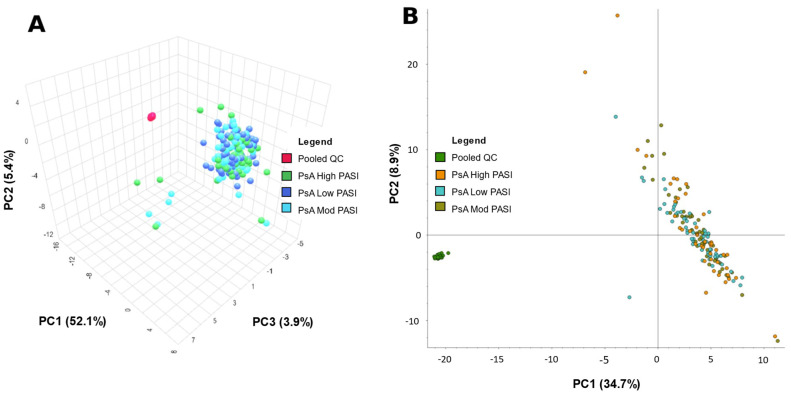
Principal component analysis (PCA) of metabolite data from PsA patients from negative mode data acquisition. (**A**) PCA—PC1: 52.1%, PC2: 5.4%, PC3: 3.9% of patients with psoriatic arthritis (PsA) and pooled quality control (PooledQC). High-skin-disease-activity PsA, low-disease-activity PsA, moderate-disease-activity PsA, and PooledQC are represented in green, dark blue, light blue, and red, respectively. The data shown were processed via Compound Discoverer 3.3 then exported to MetaboAnalyst. (**B**) PCA—PC1: 34.7%, PC2: 8.9%—of PsA patients and pooled QCs. High-skin-disease-activity PsA, low-disease-activity PsA, moderate-disease-activity PsA, and PooledQC are represented in orange, teal, brown, and green, respectively. Data shown was obtained on Compound Discoverer 3.3.

MetaboAnalyst and Compound Discoverer 3.3 were both used to generate a visual representation of the overall positive and negative mode dataset from all samples via a principal component analysis (PCA) (Figure 1 and Figure 2, respectively). Figure 1A was created by importing positive mode data processed from Compound Discoverer 3.3 into MetaboAnalyst. Figure 1B utilizes the same positive mode dataset, but was created within Compound Discoverer 3.3. Figure 2 is analogous to Figure 1, but uses negative mode data. The PCA was used to identify any differences between patients classified by PASI. The quality of data were also assessed via the spread and location of pooled QCs in the PCA plots. Their tight clustering indicates strong instrumental stability during data acquisition. However, the low-, moderate-, and high-PASI groups were not separated in Figure 1 and Figure 2.

### 2.2. Machine Learning Models

**Figure 3:**
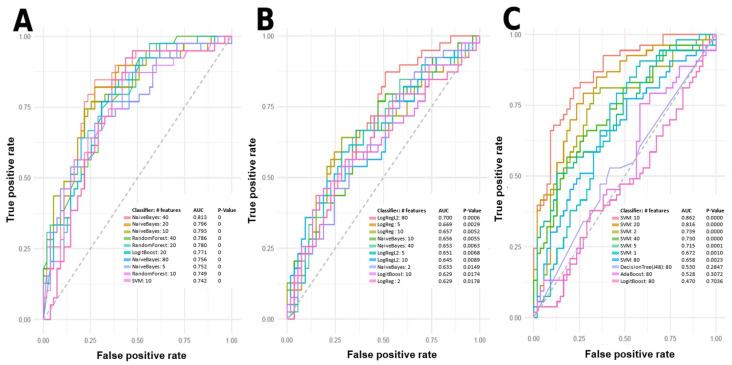
Top-performing models according to the receiver operating characteristic (ROC) curve for predicting skin disease activity in PsA patients based on positive mode data. Models include support vector machine (SVM), logistic regression (LogReg), LogitBoost, Naïve Bayes, Random Forest, Ridge Regression (LogRegL2), J48 Decision Tree, and Adaptive Boosting (AdaBoost). (**A**) Best ROC curves of predictive single or combinations of features for low vs. high disease activity. (**B**) Best ROC curves of predictive single or combinations of features for moderate vs. high disease activity. (**C**) Best ROC curves of predictive single or combination of features for low vs. moderate disease activity. AUC: Area under the receiver operating characteristic curve.

**Figure 4:**
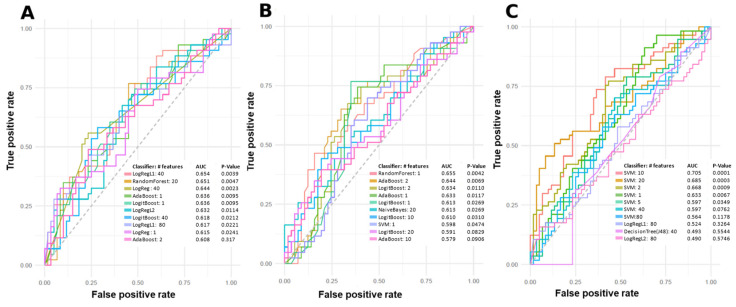
Top-performing models according to the receiver operating characteristic (ROC) curve for predicting skin disease activity in PsA based on negative mode data. Models include: support vector machine (SVM), logistic regression (LogReg), Random Forest, Lasso Regression (LogRegL1), Adaptive Boosting (AdaBoost), LogitBoost, Ridge Regression (LogRegL2), Naïve Bayes, and J48 Decision Tree. (**A**) ROC curves of predictive single or combinations of features for low vs. high disease activity. (**B**) ROC curves of predictive single or combinations of features for moderate vs. high disease activity. (**C**) ROC curves of predictive single or combinations of features for low vs. moderate disease activity. AUC: Area under the receiver operating characteristic curve.

Machine learning (ML) models were used to predict PsA patients with low, moderate, and high skin disease activity using metabolomic data. The top-performing predictive models using individual and combinations of features are summarized in Figure 3 and Figure 4. Features for model building were ranked and selected based on their individual area under the curve (AUC) scores for predicting class labels. Models shown in Figure 3 and Figure 4 were built using various algorithms and features, and only models with an AUC equal to or greater than 0.7 were considered to include potential metabolite markers of disease activity. Features in these models were tentatively identified. Many features from the positive mode dataset can be used to predict between low disease activity and moderate disease activity, low disease activity and high disease activity, and between moderate disease activity and high disease activity. Figure 3A shows the ten best models for predicting low and high disease activity, with the worst-performing model having an AUC of 0.742, and the best-performing model having an AUC of 0.813. Figure 3B shows the ten best models for predicting moderate and high disease activity. However, only LogRegL2, with 80 features, has an AUC of 0.7. Figure 3C shows the ten best models for predicting low and moderate disease activity, with the best five having AUC scores between 0.715 and 0.862. Figure 4C contains a single negative mode model with AUC > 0.7, predicting low and moderate disease activity.

### 2.3. Tentative Metabolite Identification

Features used in the models were tentatively identified using the Human Metabolome Database (HMDB). Other databases, MS-DIAL and Global Natural Product Social (GNPS), were used to identify MS level 2 matches, but no matches for statistically significant features were found. Below are Table 2, Table 3, Table 4 and Table 5, containing a summary of the top 10 predictive features with tentatively identified endogenous metabolites. Positive and negative mode features in Table 2, Table 3, Table 4 and Table 5 are shown as images of peak shape and quality in Tables S1 and S2, respectively. See Tables S3–S10 for a complete list of tentatively identified compounds in positive and negative mode, respectively. 

### 2.4. Overlapping Features

Table 6 is a list of overlapping features from different models that classify between two different disease severity groups. Eight features are shared between models that predict low vs. high skin disease activity and moderate vs. high skin disease activity groups. Three features are shared between models that predict moderate vs. high and low vs. moderate disease activity groups. One feature is shared between models that predict low vs. moderate or high disease activity groups. One feature is shared between all disease activity groups. The corresponding peak images can be found in Table S11.

## 3. Discussion

There are two aims in this study: (1) to determine if there are metabolome differences between PsA patients of low, moderate, and high skin disease activity based on PASI; and (2) to identify candidate metabolomic markers associated with skin disease activity. 

### 3.1. Models

The serum metabolome differences between PASI classifications were initially assessed with PCA plots. PCA clusters patients or samples by grouping them based on their similarities, correlations and covariances with each other. However, PCAs in Figure 2 and Figure 3 do not demonstrate meaningful and visible separation between PASI classes based on metabolite features gathered in this study. Considering that the existing literature has shown metabolome differences between PsA disease activity, the lack of separation suggests that PASI scores may not reflect subclinical metabolome differences between PsA patients of varying disease activity. On the other hand, in Figure 3 and Figure 4, ML models built with select features were able to determine PASI disease activity classes with good predictability. This is likely due to PCA and ML models being designed for different functions. PCA models are generally designed as a dimensionality reduction method wherein features or metabolites in the samples are linearly combined into new variables called ‘principal components’ based on their covariance [20]. This approach is good for visualizing an otherwise complex dataset, but original variables (features) may lose meaning because of their transformation into derived variables (principal components) [20]. However, the ML models selected in this study are generally applied as methods of classification to identify the features most important for identifying differences in the sample groups (i.e., classes) [21]. Furthermore, the features selected for the models were selected based on how informative these features are for predicting class labels, and non-informative features are removed [21]. Using the AUC to measure the performance of these classification models, it is concluded that the Naïve Bayes (with 40 features), LogRegL (with 80 features), and SVM (with 10 features) model in Figure 3 has the best performance when predicting low vs. high, moderate vs. high, and low vs. moderate disease activity, respectively, in positive mode. However, only the SVM 10-feature model was able to exceed the minimum standard for acceptable performance, with an of AUC > 0.7, and only when predicting low and moderate skin disease activity (Figure 4C). Overall, there are metabolome differences between PsA patients of varying skin disease severity.

### 3.2. Metabolite Identification and Biological Significance

Another aim of this study was to identify candidate metabolites that can predict PsA skin disease activity. The features selected in building the ML models can be identified to elucidate the specific pathways involved in PsA disease progression. Biologically interesting features tentatively identified include acylcarnitines, oxylipins, lipids, and other compound classes. 

#### 3.2.1. Overlapping Features

Table 6 shows the list of features that were used to create multiple model groups that classify samples between 2 disease severity groups. It is likely that these overlapping features are metabolites that are uniquely present for a specific disease severity group, or their concentrations are noticeably different in that specific disease severity group compared to other disease severity groups. Thus, the skin disease progression of PsA could potentially be divided into stages wherein certain biochemical reactions are active at a stage of the disease but are less active or inactive at another stage. However, given the lack of information of these specific metabolites in the literature, it is hard to draw conclusions about which biochemical reactions are of interest in different PsA skin disease activity stages. 

#### 3.2.2. Positive Mode

One such tentatively identified metabolite is platelet-activating factor (PAF), a phospholipid that mediates inflammation, immune responses, and platelet activation and aggregation [22]. PAF has pro- or anti-inflammatory properties by influencing the production of pro- or anti-inflammatory cytokines, regulates T cell activity, affects the endothelial barrier between tissues and blood vessels, and has other mechanisms [22,23,24]. In addition, platelet aggregation and activation, functions of PAF, have been positively correlated with PASI and are increased in PsA patients [25]. PAF is a tentatively identified metabolite used in models predicting low vs. high and low vs. moderate disease activity scored by PASI. 

Phospholipids are a group of metabolites tentatively identified in features used to build models predicting all three classes. The cell membrane is largely composed of phospholipids, and is involved in processes such as cell metabolism, communication, transport, and other cellular functions [26]. A disturbance in membrane phospholipid homeostasis can result in the dysfunction of these cellular functions in many disease states [26]. In patients with PsA or psoriasis, phospholipids experience greater reactive oxidative species (ROS)-mediated metabolism compared to healthy individuals, leading to greater amounts of phospholipid products, including oxidized phospholipids [23,26]. Alternatively, oxidized phospholipids can be generated through oxidation via enzymes such as lipoxygenase [23,24]. 

Oxidized phospholipids (OxPLs) are another group of metabolites tentatively identified among features of models predicting all three classes. These metabolites facilitate a wide range of functions via the activation of receptors, intracellular signaling, mediating transcription factors, and activation of cell stress pathways [23]. OxPLs with a similar structure to PAF can bind to PAF receptors to mediate inflammation and platelet aggregation [22,23]. OxPLs also bind to prostaglandin receptors to activate integrins, facilitating leukocyte recruitment and inflammation [23]. STAT3, a transcription factor regulating the expression of genes for IL-8, a pro-inflammatory chemokine, can be activated by OxPLs [23]. STAT3 is also upregulated in both PsA and psoriasis patients [26]. Oxidized phospholipids have been found to be elevated in psoriatic skin, and are also associated with cardiovascular disease, a comorbidity of PsA [24]. 

Some features used in all three comparison type models were tentatively identified as acylcarnitines. Acylcarnitines are metabolites involved in fatty acid metabolism and energy production via beta-oxidation [27]. Psoriasis patients have been shown to exhibit increased levels of acylcarnitine and carnitines in comparison to healthy individuals [27,28]. However, information on the roles of specific acylcarnitines is sparse in the literature.

Oxylipins and their subset, eicosanoids, were components in models predicting low vs. high and low vs. moderate disease activity. Oxylipins are typically formed from poly-unsaturated fatty acids via enzymatic reaction with cyclooxygenases, lipoxygenases, and cytochrome p450s, or via non-enzymatic reactions [29,30]. Tentatively identified oxylipins include 12-HETE, Leukotriene B4, and prostaglandins that are pro-inflammatory and are positively correlated with joint disease activity in PsA patients [30,31,32]. Leukotriene B4 is a lipid chemoattractant that recruits leukocytes to sites of inflammation, and 12-HETE can promote oxidative stress and impacts the signaling pathways involved in inflammation [31,32]. 

Proline is another tentatively identified metabolite involved in the prediction of high disease activity vs. low or moderate disease activity. Proline is an important amino acid for the synthesis of collagen, and may be associated with psoriasis and PsA skin lesions [33]. PsA patients have been found to exhibit increased amounts of degradation products of collagen in the serum that may originate from cartilage destruction or skin lesions, and high collagen turnover is suspected in the skin lesions of psoriasis patients [34]. In addition, as shown in Table 6, proline is a metabolite that could be associated with high disease activity. Given that the PASI was used to classify disease severity, it is likely that proline concentration is abnormal due to high collagen turnover in the skin lesions, or joint cartilage of high-disease-activity-PsA patients.

These models also identified diacylglycerols (DAGs) and monoacylglycerols (MAGs) as contributors to disease severity. Although information about the role of the specific DAGs and MAGs tentatively identified is very sparse in the literature, the compound classes are involved in immune signaling, and regulation. DAGs can act as secondary messengers and drive the activation, proliferation, and effector functions of several innate and adaptive immune cells [35]. DAGs can indirectly activate the nuclear factor kappa light chain enhancer of activated B cells (NF-KB) and extracellular regulated kinase (ERK) pathways of T cells, B cells, macrophages, and more [35]. MAGs can activate immune receptors including PPARs that regulate lipid metabolism and control energy production in the mitochondria, inflammation, cytokine secretion, and more [36]. 

Many other tentatively identified metabolites in positive mode belong to lipid and fatty acid compound classes, such as medium- and long-chain fatty acids. In general, these compound classes are noted to be involved in inflammation and several diseases with similar manifestations to PsA. Multiple long- and medium-chain fatty acids have been found to be significantly different between PsA and healthy patients [37]. The roles of fatty acids include being precursors to pro- and anti-inflammatory eicosanoids, and regulating immune homeostasis via altering cytokine production, leukocyte recruitment, and more [30,37,38]. Overall, the results highlight dyslipidemia among PsA patients, and that lipids and fatty acids can play roles in immune-mediated inflammation and PsA pathogenesis.

#### 3.2.3. Negative Mode

In negative mode, there was only one model created that had an AUC equal or greater than 0.7, and this model predicts between low vs. moderate disease activity (Figure 4C). The features used in that model were identified, and similarly to positive-mode models, bile acids and phospholipids were tentatively identified. 

Bile acids (Table 5) include Glyco-beta-muricholic acid and Glycoursodeoxycholic acid. Bile acids were tentatively identified in models distinguishing between moderate disease activity and high disease activity. Bile acid metabolites in general are involved in lipid absorption and can mediate immune signaling [39,40]. The receptors Farnesoid-X-Receptor (FXR) and G-protein bile acid receptor 1 (GPBAR1) are among multiple receptors that can be activated by bile acids, and are present on many immune cells [39,40]. The activation of these receptors on macrophages, dendritic cells, and natural killer T cells can modulate immune response [40]. FXR activation can block NF-KB-mediated pro-inflammatory responses [39,40]. GPBAR1 activation is also responsible for regulating pro-inflammatory responses in innate immune cells [40]. Furthermore, these receptors are involved in the regulation of the nucleotide-binding domain, leucine-rich-containing family, and pyrin domain-containing-3 (NLPR3) inflammasome [40]. A study by Paine et al. found ester versions of these bile acids were significantly lower in PsA patients compared to psoriasis patients [39]. Phosphatidylcholine, phosphatidylethanolamine, their oxidized versions, and methyl phosphatidylethanolamine are phospholipids that were tentatively identified in negative mode. 

Another interesting feature used in this model is Indoxyl sulfate (Table 5), a metabolite of Tryptophan and a uremic toxin that accumulate in the body under chronic kidney disease [41,42,43]. This metabolite is associated with impairment of bone turnover via inhibition of osteoblast and osteoclast maturation [43]. Furthermore, this metabolite has been shown to enhance oxidative stress, thereby promoting pro-inflammatory cytokines [41,42]. Thus, indoxyl sulfate could be a potential metabolite of PsA that exhibits inflammatory responses and bone-related health effects.

#### 3.2.4. Limitations

The results from this study agree with the literature examining metabolomic differences between PsA patients of varying disease activity, or that comparing PsA, psoriasis, and healthy individuals. However, this study is limited by several factors. Although all samples were sourced from patients suffering from PsA, the observed metabolome differences could be confounded by comorbidities, diet, and pharmacotherapy (Table 1). Some tentatively identified metabolites included unrelated drugs, food biomarkers, and metabolites associated with other diseases that could influence the expression of other metabolites. Comorbidities like hypertension, diabetes, and hyperlipidemia share common inflammatory pathways involving inflammatory cytokines such as tumor necrosis factor (TNF) [44]. Given these diseases share common pathways, they may also share common metabolite expressions that could confound the results of our machine learning. According to Tables S4 and S6, our method detected metabolites such as Hydroxyibuprofen, which is associated with Ibuprofen, a non-steroidal anti-inflammatory drug (NSAID). The presence of NSAIDs and other drugs is known to alter the metabolomic profile of mouse serum, especially for lipid and fatty acid compound classes [45]. In addition, there was a very wide range in PASI scores for high disease activity, from approximately 10 to 55, whereas low and moderate PASI scores approximately span a range of 5. Although this is the general rule applied in a clinical setting, it is uncertain whether a patient with a PASI score of 10 and a PASI score of 55 will exhibit similar disease-related metabolomic markers. Ideally, a control group of psoriasis patients classified into three groups based on PASI and who do not have PsA would have helped us identify PsA-specific markers. However, we have not included such a comparison, which is a significant limitation. 

Another point of improvement for this study would be to use a multiple chromatographic methodology and SPME coatings. Although this study used 1:1 HLB:PS-DVB WAX, a coating that was evaluated to extract a wide range of compounds [46], using multiple specialized coatings may greatly expand the metabolome coverage for analysis. Similarly, this study used a chromatographic method developed for fatty acids, but use of other methods such as an HILIC method for more polar metabolites could also expand the metabolome coverage for analysis. This expanded coverage could uncover other classes of compounds that allow for insights into the disease state. 

## 4. Materials and Methods

### 4.1. Patients 

**Figure 5:**
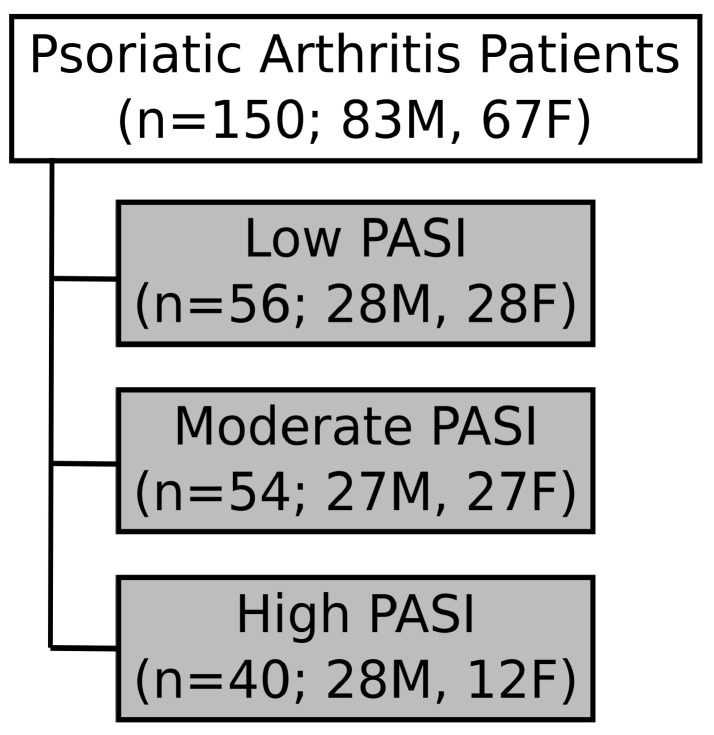
Grouping of patient samples based on skin disease severity. Serum samples were obtained from patients with psoriatic arthritis (PsA; n = 150). PsA patients were classified, based on the Psoriatic Area Severity Index (PASI), as low (n = 56), moderate (n = 54), or high disease activity severity (n = 40). The samples were age-balanced and sex-matched for PsA patients. However, due to low numbers of patients in the high-PASI group, only 28 male and 12 female PsA patients were included. M: Male. F: Female.

Serum samples of psoriatic arthritis (PsA) patients satisfying classification criteria (CASPAR) [47] were obtained from the Schroeder Arthritis Institute Psoriatic Disease Research Program Biobank. All samples were collected at Toronto Western Hospital (2006–2021). The PASI of PsA patients was assessed by a trained rheumatologist during their visit. Patients were further categorized into three subgroups based on skin disease severity: mild (PASI: <5), moderate (PASI: 5–10), and high (PASI: >10) (Figure 5). Patients with active infections (<3 months), recent cardiac events/heart disease (<6 months), recent surgery (<6 months), and cancer were excluded from the study, along with patients receiving any treatment with biologics or targeted synthetic drugs. Sub-groups were sex- and age-balanced to the fullest possible extent. Full ethics approval was received through the University Health Network Research Ethics Board. A summary of patient information such as sex, age, duration of psoriasis, duration of PsA, treatment, and associated comorbidities per group can be found in Table 1. Individual patient information can be found in Tables S12–S14.

### 4.2. Materials

We purchased stainless steel combs and the manual concept-96 unit from PAS technologies (Magdala, Germany), and the dip coater and lab oven from Ni-Lo Scientific (Ottawa, ON, Canada) and Hogentogler (Columbia, MD, USA), respectively. Oasis hydrophilic–lipophilic balanced (HLB) particles (45 μm) and polystyrene divinylbenzene with weak anion exchanger (PS-DVB-WAX) particles (45–65 μm) were purchased from the Waters Corporation (Milford, MA, USA). HPLC- and LC-MS-grade solvents (acetonitrile, methanol, water, isopropanol, and acetone), concentrated hydrochloric acid, N,N-dimethylformamide (DMF), formic acid, and L-ascorbic acid were purchased from Fisher Scientific (Waltham, MA, USA). We purchased the following internal standards and chemicals: arachidonic acid-d8, nordiazepam-d5, diazepam-d5, and polyacrylonitrile (Millipore Sigma [Burlington, MA, USA]), dihomo-gamma-linolenic acid-d6, docosahexaenoic acid-d5, ketoconazole-d3, lignoceric acid-d3, progesterone-d9, docosapentaenoic acid-d5, eicosapentaenoic acid-d5, itraconazole-d5, alpha-linolenic acid-d5, oxazepam-d5, palmitoleic acid-d13, and taurocholic acid-d4 (Cayman Chemicals [Michigan, MI, USA]), and polyunsaturated fatty acid LC-MS mixture, saturated/monounsaturated fatty acid LC-MS mixture, short-chain fatty acid LC-MS mixture, and short-chain fatty acid mixture 2 (Cayman Chemicals, Ann Arbor, MI, USA). Deep 1 mL 96-well plates were purchased from Canadian Life Science (Peterborough, ON, Canada).

### 4.3. Quality Assurance and Quality Control

For quality assurance and quality control, the samples and desorption solution were injected with isotopically labeled compounds as internal standards. The purpose of internal standards within the desorption solution is to monitor the instrument stability during data acquisition. The internal standards within the samples allow the monitoring of extraction efficiency and technical variability during the sample preparation process in addition to the instrument stability during data acquisition.

Briefly, 200 μL of each sample was diluted with 400 μL of phosphate-buffered saline (PBS), which contained the following deuterated standards: docosapentaenoic acid-d5, eicosapentaenoic acid-d5, itraconazole-d5, alpha-linolenic acid-d5, oxazepam-d5, palmitoleic acid-d13, taurocholic acid-d4, and nordiazepam-d5. The resulting concentration of each internal standard prior to extraction was 250 ng/mL. The desorption solution contained the following deuterated standards at a concentration of 250 ng/mL: dihomo-gamma-linolenic acid-d6, ketoconazole-d3, diazepam-d5, progesterone-d9, lignoceric acid-d3, docosahexaenoic acid-d3, and arachidonic acid-d8. 

Internal standards oxazepam-d5, itraconazole-d5, taurocholic acid-d4, and nordiazepam-d5 were recovered in the samples, while ketoconazole-d3, diazepam-d5, progesterone-d9, docosahexaenoic acid-d5, and dihomo-gamma-linolenic acid-d6 were recovered in desorption solution in positive mode, with a maximum variation of 34%. In negative mode, internal standards oxazepam-d5, docosapentaenoic acid-d5, taurocholic acid-d4, and eicosapentaenoic acid-d5 were recovered in the sample, while docosahexaenoic acid-d5, dihomo-gamma-linolenic acid-d3, arachidonic acid-d8, and lignoceric acid-d3 were recovered in desorption solution in negative mode, with a maximum variation of 35%. 

Instrument stability during data acquisition was monitored via periodic injection of instrumental quality controls (InstQCs) after every ten samples. 

In positive mode, the following compounds were monitored: stearidonic acid, alpha-linolenic acid, eicosapentaenoic acid, arachidonic acid, dihomo-gamma-linoleic acid, docosahexaenoic acid, docosapentaenoic acid, adrenic acid, 18:1(d7) Monoacylglyceride, 18:1(d7) Lyso PE, 18:1(d7) Lyso PC, 15:0-18:1(d7) diacylglyceride, 18:1(d7) chol ester, 15:0-18:1(d7) PE, d18:1-18:1(d9) SM, 15:0-18:1(d7) PC, trans-m-Coumaric acid, 18:1 monoacylglyceride, 18:1 Lyso PE, C15 ceramide, 15:0-18:1 diacylgleride, 15:0-18:1 PE, d18:1-18:1 SM, and 15:0-18:1 PC. The compounds were recovered with variations between 7–25%.

In negative mode, the following compounds were monitored: Hexanoic acid, Octanoic acid, nonanoic acid, decanoic acid, dodecanoic acid, lauric acid, myristic acid, palmitoleic acid, palmitic acid, stearidonic acid, alpha-linolenic acid, gamma-linolenic acid, linoleic acid, stearic acid, eicosapentaenoic acid, arachidonic acid, dihomo-gamma-linoleic acid, arachidic acid, docosahexaenoic acid, docosapentaenoic acid, adrenic acid, nervonic acid, lignoceric acid, 15:0-18:1(d7) PE, 18:1(d7) Lyso PE, dodecanedioic acid, 1,11-undecanedicarboxylic acid, 15:0-18:1 PE, 18:1 Lyso PE, and C15 ceramide. The compounds were recovered with variations between 2–22%.

### 4.4. Preparation of Solid-Phase Microextraction (SPME) Devices

SPME devices were prepared using a dip-coating method optimized in the Schroeder Arthritis Institute—Centre for Arthritis Diagnostic and Therapeutic Innovation: Metabolomics Core Facility. Stainless steel blades were first cleaned with HPLC-grade IPA (sonicated, 30–45 min) followed by HPLC-grade water (sonicated, 10–15 min). The blades were then etched in concentrated hydrochloric acid for 1 h in accordance with a previously well-established protocol [48]. After washing thoroughly with distilled water, blades were dried in an oven overnight at 45 °C. A specialized software-operated dip-coating machine was used to coat the stainless steel support with a slurry mixture consisting of 7% *w*/*v* 1:1 HLB and PS-DVB-WAX particles in 7% polyacrylonitrile (PAN) solution. After each layer of particles was applied, the freshly coated blades were placed in an oven to cure for 1 min at 150 °C. A total of 12 layers were applied to each blade, which resulted in a final coating dimension that was 2 cm long with an average thickness of 0.3 mm. Blades were assembled to form SPME brushes, which were capable of performing high-throughput extractions in 1 mL deep well 96-well plates. Before use, the devices were cleaned with a solution of 50:25:12.5:12.5 (*v*/*v*/*v*/*v*) water:methanol:acetonitrile:isopropanol using the concept-96 manual kit (20 min, 1500 rpm, 30 °C, repeat 3 times).

### 4.5. Solid-Phase Microextraction (SPME) for Sample Preparation

Serum sample extracts were prepared using SPME devices, which consisted of thin stainless steel blades coated with a 1:1 mixture of HLB and PS-DVB-WAX particles. All samples were block randomized across two 96-well plates, and 200 μL of each serum sample was added to its respective well. After gentle agitation of the as-prepared samples for 30 min at 500 rpm, samples were ready for SPME extraction. Prior to extraction, SPME devices were mounted on the Concept-96 manual kit and conditioned in a mixture of 1:1 methanol:water (*v*/*v*) for 30 min at 1500 rpm. Following conditioning, the device was rinsed in water for 15 min at 1500 rpm. Immediately after device pre-conditioning and rinsing, agitated serum samples in PBS were exposed to the blades for 1 h at 1500 rpm for extraction of metabolites onto the solid phase coating. After extraction, the device was rinsed in a mixture of 90:5:5 (*v*/*v*/*v*) water:methanol:acetone for 10 s at 500 rpm to remove any loosely attached matrix components. Metabolites extracted onto the devices were desorbed for 1 h at 1500 rpm in 600 μL of 4:3:3 methanol:acetonitrile:water (*v*/*v*/*v*) + 0.1% ascorbic acid. For optimal compatibility with the LC-MS methodology, the desorption solution was diluted with 400 μL of 45:30:25 soproanol:acetonitrile:methanol (*v*/*v*/*v*) to produce a final extract composed of 3:3:3:1 isopropanol:methanol:acetonitrile:water) (*v*/*v*/*v*/*v*). A pooled quality control (QC) sample was prepared by combining 10 μL of each sample extract. The pooled QC was injected approximately every 10 sample injections during instrumental acquisition.

### 4.6. Instrumental Analysis

A liquid chromatography–high-resolution mass spectrometry (LC-HRMS) method for fatty acid analysis of serum samples was used to perform a discovery-based untargeted analysis in this study. The analysis was conducted at the Schroeder Arthritis Institute—Centre for Arthritis Diagnostic and Therapeutic Innovation: Metabolomics Core Facility using a Vanquish autosampler and pump coupled to a Q-Exactive Plus Hybrid Quadrupole-Orbitrap Mass Spectrometer (Thermo Fisher Scientific, Waltham, MA, USA). An accucore C30 HPLC column (100 mm × 2.1 mm, 2.6 μm) (Thermo Fisher Scientific, Waltham, MA, USA) was used for chromatographic separation. For positive ion mode analysis, chromatographic separation was achieved via gradient elution over 30 min using mobile phases consisting of 99.9/0.1 water/formic acid (*v*/*v*) and 99.9/0.1 methanol/formic acid (*v*/*v*). The mass spectrometer was run at high resolution (70,000), and data were acquired within an *m*/*z* range of 75–1000 with an automatic gain control target of 1E6 and an injection time of 50 ms. For negative ion mode analysis, chromatographic separation was achieved via gradient elution over 20 min using mobile phases consisting of 99.9/0.1 water/formic acid (*v*/*v*) and methanol. The mass spectrometer was run at high resolution (70,000), and data were acquired within an *m*/*z* range of 75–1000 with an automatic gain control target of 1 × 10^6^ and an injection time of 50 ms. Further details on the gradient elution used can be found in Table 7 and Table 8. 

### 4.7. Data Pre-Processing and Statistical Analyses 

Acquired LC-MS data files were pre-processed using Compound Discoverer 3.3 [49], proprietary data processing software developed by Thermo Fisher Scientific (Waltham, USA). With Compound Discoverer 3.3 (Thermo Fisher Scientific [Waltham, MA, USA]), the data were normalized via SERRF, a QC-based normalization method for large-scale untargeted metabolomics data. Peak lists generated by both platforms were filtered to meet the following conditions: (1) Positive mode: features with a pooled-QC: solvent-blank or pooled-QC:fiber-blank peak intensity ratio of <5 were removed from further statistical evaluation. (2) Negative mode: features with a pooled-QC: solvent-blank or pooled-QC:fiber-blank peak intensity ratio of <5 were removed. All multivariate data analysis was performed using MetaboAnalyst 5.0, wherein the data were subjected to a generalized log-transformation and pareto scaling unless otherwise stated. 

Predictive features were identified by first testing each feature individually with five classifiers: linear discriminant analysis (LDA), Naïve Bayes (NB), support vector machine (SVM), logistic regression (LR) and random forest (RF). Each feature received an area under the curve (AUC) score from each classifier, and then features were ranked by their best AUC scores. The top k features were then used as an input for each classifier, where k was 1, 2, 5, 10, 20, 40, 80. Model testing was conducted using 10-fold cross-validation (10-fold CV).

## 5. Conclusions

In this study, sample preparation with Thin-Film SPME with LC-HRMS analysis facilitated the untargeted metabolomics assessment of psoriasis disease activity in serum samples obtained from PsA patients classified according to the PASI. The PCA did not exhibit any clear separation between PASI-based disease activity groups, but machine learning models were able to predict disease activity to an acceptable degree of success. Features used in the creation of successful ML models were tentatively identified, and commonly belong to compound classes like bile acids, lipoxins, and phospholipids. These compounds have been found to be associated with pathways such as fatty acid metabolism and immune-mediated pathways that have been implicated in PsA. This opens a possible direction for future research in metabolite differences based on disease severity in psoriatic arthritis disease. A follow-up study using the same SPME-LC-MS method should be conducted to confirm the results, but should include rigorous patient selection to account for comorbidities, treatments, and should also include rigorous MS/MS spectral validation.

## Figures and Tables

**Table 1:** Summary of demographics and disease information of patients participating in study.

Group	Sex (No. of Patients)	Mean Age *Standard Deviation* (Age Range)	Mean BMI *Standard Deviation* (BMI Range)	PASI Score Mean *Standard Deviation* (Range)	Dur. Psoriasis Mean *Standard Deviation* (Range) (Years)	Dur. PsA Mean *Standard Deviation* (Range) (Years)	Treatment (No. Patients)	Comorbidities (No. Patients in Group)
PsA	M (28)	44.4	27.6	2.0	17.6	9.3	NSAIDs (17)	Hypertension (5)
(Low)		*15.4*	*5.4*	*1.3*	*11.5*	*9.1*	DMARDs (9)	Depression (1)
		(18.1–74.6)	(16.2–42.6)	(0.3–4.8)	(0.5–53.6)	(0.0–25.9)	Steroids (1)	Hyperlipidemia (6)
								Diabetes (3)
	F (28)	47.9	28.5	1.8	22.1	9.5	NSAIDs (19)	Hypertension (2)
		*10.0*	*7.4*	*1.4*	*13.6*	*8.5*	DMARDs (10)	Depression (3)
		(30.6–67.6)	(19.8–49.3)	(0.1–4.8)	(0.5–46.8)	(0.5–28.8)	Steroids (1)	Hyperlipidemia (6)
PsA	M (27)	51.0	28.5	6.4	22.5	13.6	NSAIDs (19)	Hypertension (1)
(Moderate)		*10.1*	*6.9*	*1.4*	*12.7*	*10.5*	DMARDs (11)	Depression (2)
		(28.9–73.7)	(19.8–52.1)	(5.0–9.8)	(3.1–50.1)	(0.6–34.7)		Hyperlipidemia (3)
								Diabetes (1)
	F (27)	47.1	29.1	7.2	23.7	10.2	NSAIDs (15)	Hypertension (1)
		*12.1*	*6.6*	*1.4*	*11.0*	*10.0*	DMARDs (14)	Depression (3)
		(19.3–71.6)	(18.2–41.7)	(5.1–9.4)	(3.3–44.6)	(0.2–37.1)	Steroids (1)	Hyperlipidemia (3)
PsA	M (28)	46.5	27.7	21.1	18.8	7.9	NSAIDs (15)	Hypertension (3)
(High)		*13.5*	*4.6*	*9.6*	*11.0*	*9.7*	DMARDs (11)	Depression (2)
		(22.5–81.2)	(18.8–34.3)	(10.1–40.7)	(5.5–45.2)	(0.0–37.3)	Steroids (1)	Diabetes (3)
								Hyperlipidemia (5)
	F (12)	48.0	31.0	22.5	19.4	14.0	NSAIDs (3)	Hyperlipidemia (2)
		*12.7*	*6.6*	*13.3*	*11.4*	*12.6*	DMARDs (7)	Diabetes (3)
		(27.2–71.7)	(19.1–40.1)	(10.2–54.6)	(0.4–36.5)	(0.2–32.2)	Steroids (2)	

F: female. M: male. BMI: body mass index, NSAID: non-steroidal anti-inflammatory drugs, DMARD: disease-modifying anti-rheumatic drugs. low, moderate and high indicate low, moderate and high skin disease activity based on PASI.

**Table 2:** Top 10 tentatively identified endogenous metabolites for low vs. high skin disease activity.

*m*/*z*	Retention Time (min)	Adduct	Monoisotopic Mass	AUC	Tentative Identification
546.3528	14.59	[M + Na]^+^	523.3638	0.59	Platelet-activating factor; 6 other hits
544.3373	13.35	[M + Na]^+^	521.3481	0.58	LysoPC(0:0/18:1(9Z)); 5 other hits
116.0708	0.60	[M + H]^+^	115.0633	0.58	Proline
427.1938	5.74	[M + NH_4_]^+^	409.1584	0.58	dermatan L-iduronate; 1 other hit
496.3397	12.95	[M + Na]^+^	473.3505	0.57	Clupanodonyl carnitine; 5 other hits
373.2735	9.79	[M + H]^+^	372.2664	0.57	Cervonoyl ethanolamide
520.3398	12.33	[M + H]^+^	519.3325	0.57	LysoPC(0:0/18:2(9Z,12Z)); 1 other hit
370.2950	8.20	[M + NH_4_]^+^	352.2614	0.57	MG(18:3(6Z,9Z,12Z)/0:0/0:0); 8 other hits
289.1409	5.12	[M + Na]^+^	266.1518	0.57	pentadeca-5,7,9-trienedioic acid; 8 other hits *
356.2793	7.49	[M + NH_4_]^+^	338.2457	0.57	11,12-DiHETrE; 8 other hits

* Clear peak, but insufficient quality; AUC: Area under the receiver operating characteristic curve.

**Table 3:** Top 10 tentatively identified endogenous metabolites for moderate vs. high skin disease activity.

*m*/*z*	Retention Time (min)	Adduct	Monoisotopic Mass	AUC	Tentative Identification
211.1441	3.90	[M + NH_4_]^+^	193.1103	0.60	(R)-N-Methylsalsolinol; 3 other hits
215.1279	4.78	[M + H]^+^	214.1205	0.58	undec-3-enedioic acid; 3 other hits *
769.4224	21.08	[M + Na]^+^	746.4370	0.57	PG(20:4(8Z,11Z,14Z,17Z)-2OH(5S,6R)/i-12:0); 5 other hits
760.5845	20.22	[M + H]^+^	759.5778	0.57	Pe-NMe2(20:1(11Z)/15:0); 26 other hits
802.5350	19.32	[M + Na]^+^	779.5465	0.57	PC(20:5(6E,8Z,11Z,14Z,17Z)-OH(5)/P-16:0); 89 other hits
496.3397	12.95	[M + Na]^+^	473.3505	0.57	Clupanodonyl carnitine; 5 other hits
544.3373	13.35	[M + Na]^+^	521.3481	0.56	LysoPC(18:1(9Z)/0:0); 5 other hits
518.3215	12.95	[M + Na]^+^	495.3325	0.56	LysoPC(16:0/0:0); 2 other hits
351.2504	12.67	[M + Na]^+^	328.2614	0.56	MG(0:0/16:1(9Z)/0:0); 1 other hit
828.5507	19.67	[M + Na]^+^	805.5621555	0.56	PC(20:5(6E,8Z,11Z,14Z,17Z)-OH(5)/P-18:1(9Z)); 107 other hits

* Clear peak, but insufficient quality; AUC: Area under the receiver operating characteristic curve.

**Table 4:** Top 10 tentatively identified endogenous metabolites for low vs. moderate skin disease activity.

*m*/*z*	Retention Time (min)	Adduct	Monoisotopic Mass	AUC	Tentative Identification
311.1464	3.40	[M + NH_4_]^+^	293.1111	0.75	4-Hydroxyproline galactoside; 3 other hits
524.3710	14.21	[M + H]^+^	523.3638	0.72	Platelet-activating factor; 4 other hits
263.0887	6.70	[M + Na]^+^	240.0998	0.72	3-Carboxy-4-methyl-5-propyl-2-furanpropionic acid; 4 other hits
641.5110	20.65	[M + NH_4_]^+^	623.4761	0.71	Cer(d16:1/6 keto-PGF1alpha); 58 other hits
830.5664	19.88	[M + Na]^+^	807.5778	0.71	PC(20:4(5Z,7E,11Z,14Z)-OH(9)/P-18:1(9Z)); 151 other hits
769.4224	21.08	[M + Na]^+^	746.4370	0.70	PG(i-12:0/20:4(6Z,8E,10E,14Z)-2OH(5S,12R)); 5 other hits
188.0707	2.35	[M + H]^+^	187.0633	0.70	Indoleacrylic acid
813.6839	21.40	[M + H]^+^	812.6771	0.68	SM(d18:2(4E,14Z)/24:0); 1 other hit
377.2659	13.29	[M + Na]^+^	354.2770	0.68	Glyceryl monolinoleate
343.2241	11.17	[M + Na]^+^	320.2351	0.67	12-HETE; 39 other hits

AUC: Area under the receiver operating characteristic curve.

**Table 5:** Tentatively identified negative mode metabolites in low vs. moderate skin disease activity, using the SVM 10 model.

*m*/*z*	Retention Time (min)	Adduct	Monoisotopic Mass	AUC	Tentative ID
212.0026	3.97	[M − H]^−^	213.0096	0.72	Indoxyl sulfate; 3 other hits
729.1788	11.02	[M + Cl]^−^	694.2109	0.69	Neocuscutoside C; 3 other hits
464.3020	7.39	[M − H]^−^	465.3090	0.67	Glyco-beta-muricholic acid; 5 other hits
802.5614	13.14	[M − H]^−^	803.5676	0.67	PE(PGF1alpha/P-18:0); 38 other hits
343.1707	8.15	[M − H]^−^	344.1776	0.64	Tamoxifen-ol
667.1419	11.98	[M − H]^−^	668.1506	0.63	Etoposide Phosphate; 1 other hit
448.3073	8.18	[M − H]^−^	449.3141	0.63	Glycohyodeoxycholic acid; 7 other hits
388.1559	8.00	[M + Cl]^−^	353.1852	0.63	Epiroprim
203.08299	3.58	[M − H]^−^	204.0899	0.63	L-Tryptophan; 13 other hits

AUC: Area under the receiver operating characteristic curve.

**Table 6:** Tentative identifications of overlapping features between machine learning model groups.

Moderate vs. High (*m*/*z*)	Low vs. Moderate (*m*/*z*)	Low vs. High (*m*/*z*)	Tentative Identification
736.2599		736.2599	N/A
215.1279		215.1279	Medium chain fatty acids
680.3549		680.3549	N/A
496.3397		496.3397	Phospholipid, Acylcarnitine
159.1168		159.1168	Exposome—Benzene and derivatives
544.3373		544.3373	Lysophosphatidylcholine
688.3047		688.3047	N/A
116.0708		116.0708	Proline; Exposome-related Metabolites
415.2536	415.2536		Exposome—Peptides
769.4224	769.4224		Oxidized phospholipid
646.2575	646.2575		Exposome—Drug for increasing blood glucose concentration
	668.5443	668.5443	DAGs
549.1857	549.1857	549.1857	Exposome—Neoacrimarine I/F

N/A: No tentative identification available for this feature.

**Table 7:** Liquid chromatographic gradient used for separation in positive mode with an accucore C30 HPLC column (100 mm × 2.1 mm, 2.6 μm).

**Time (min)**	**% Mobile Phase B** **(Methanol + 0.1% Formic Acid)**
0	5
1	5
20	100 (curve 3)
22.5	100
25	5
30	5

**Table 8:** Liquid chromatographic gradient used for separation in negative mode with an accucore C30 HPLC column (100 mm × 2.1 mm, 2.6 μm).

**Time (min)**	**% Mobile Phase B (Methanol)**
0	0
2	0
12	100 (curve 3)
15	100
18	0
20	0

## Data Availability

Publicly available datasets were analyzed in this study. These data can be found here: https://massive.ucsd.edu/ProteoSAFe/dataset.jsp?accession=MSV000092967.

## Supplementary Materials

The supporting information can be downloaded at: https://www.mdpi.com/article/10.3390/ijms242015299/s1.
