# Supplementary material for: Identifying Serum Metabolomic Markers Associated with Skin Disease Activity in Patients with Psoriatic Arthritis
Source: Int J Mol Sci. 2023 Oct 18;24(20):15299. doi: 10.3390/ijms242015299 (PMC10607811; doi:10.3390/ijms242015299)
Supplement: Supplementary file 1 [file ijms-24-15299-s001.zip › ijms-2630022-supplementary.pdf]

Supplementary Information

Identifying Serum Metabolomic Markers Associated with Psoriasis Skin Disease Activity in patients with Psoriatic Arthritis

Hani Choksi [1,2,†], Shenghan Li [1,2,†], Nikita Looby [1], Max Kotlyar [3], Igor Jurisica [3,4,5], Vathany Kulasingam [2,6,*,‡] and Vinod Chandran [1,2,7,*,‡]

Affiliations:

[1] Schroeder Arthritis Program, Krembil Research Institute, University Health Network, Toronto, ON M5T 0S8, Canada; hani.choksi@utoronto.ca (H.C.); hanhan.li@mail.utoronto.ca (S.L.); nikita.looby@uhn.ca (N.L.)

[2] Department of Laboratory Medicine and Pathobiology, University of Toronto, Toronto, ON M5S 1A1, Canada

[3] Osteoarthritis Research Program, Division of Orthopedic Surgery, Schroeder Arthritis Institute and Data Science Discovery Centre for Chronic Diseases, Krembil Research Institute, Toronto, ON M5T 0S8, Canada; max.kotlyar@uhn.ca (M.K.); juris@ai.utoronto.ca (I.J.)

[4] Departments of Medical Biophysics and Computer Science, and Faculty of Dentistry, University of Toronto, Toronto, ON M5S 1A1, Canada

[5] Institute of Neuroimmunology, Slovak Academy of Sciences, Dubravská cesta 9, 845 10 Bratislava 45, Slovakia

[6] Division of Clinical Biochemistry, Laboratory Medicine Program, University Health Network, Toronto, ON M5T 0S8, Canada

[7] Division of Rheumatology, Department of Medicine, University of Toronto, Toronto, ON M5S 1A8, Canada

* Correspondence: vathany.kulasingam@uhn.ca (V.K.); vinod.chandran@uhn.ca (V.C.); Tel.: +1-4163404800 (ext. 8589) (V.K.); +1-4166035192 (V.C.)

† These authors contributed equally to this work.

‡ These authors contributed equally to this work.

Table S1: Peaks of summarized positive mode features

Feature Peak	m/z
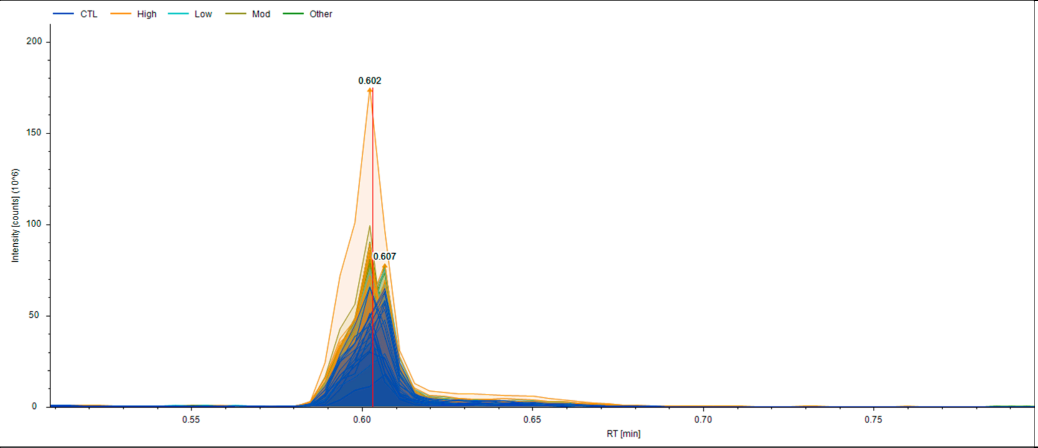	116.0708
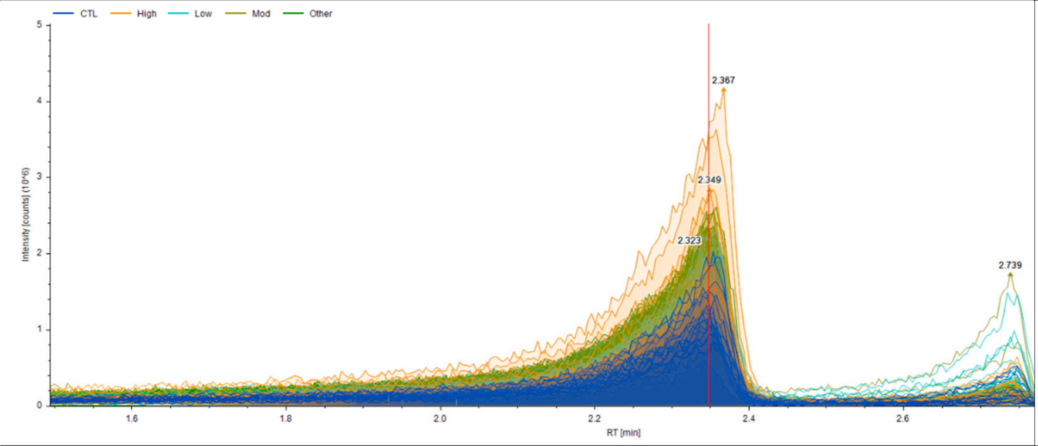	188.0707
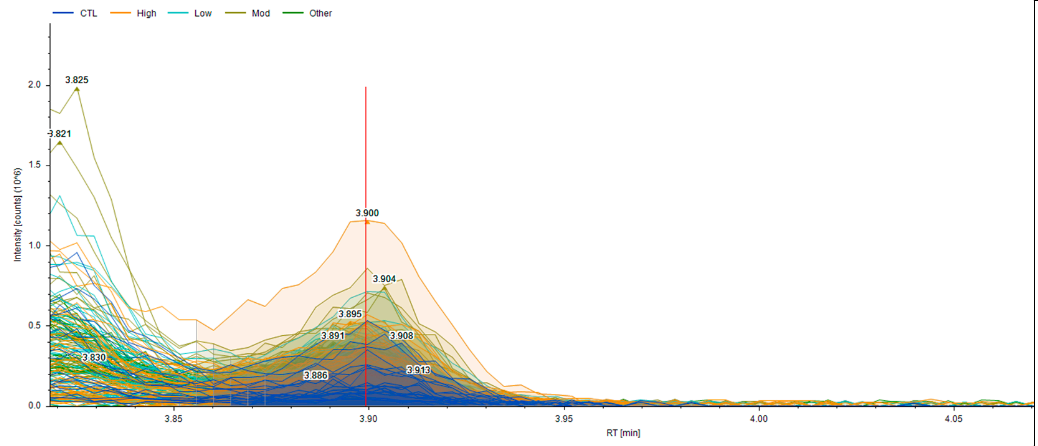	211.1441

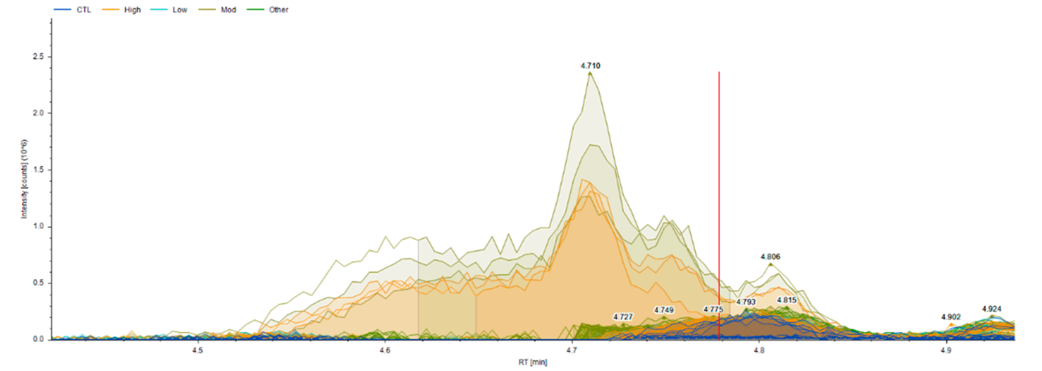	215.1279
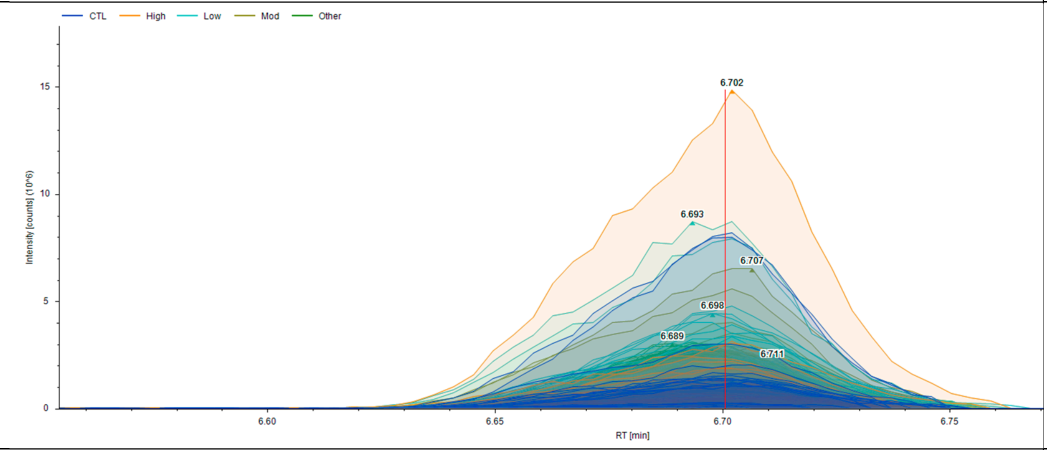	263.0887
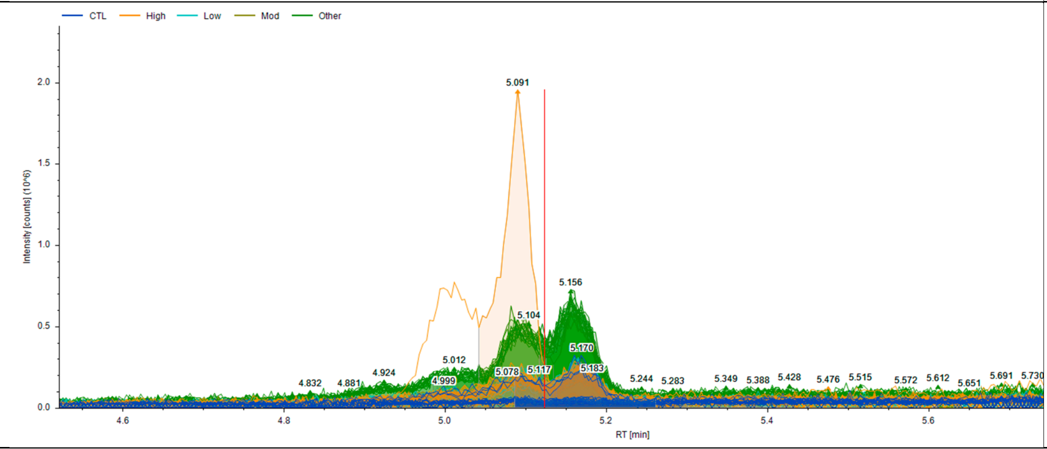	289.1409

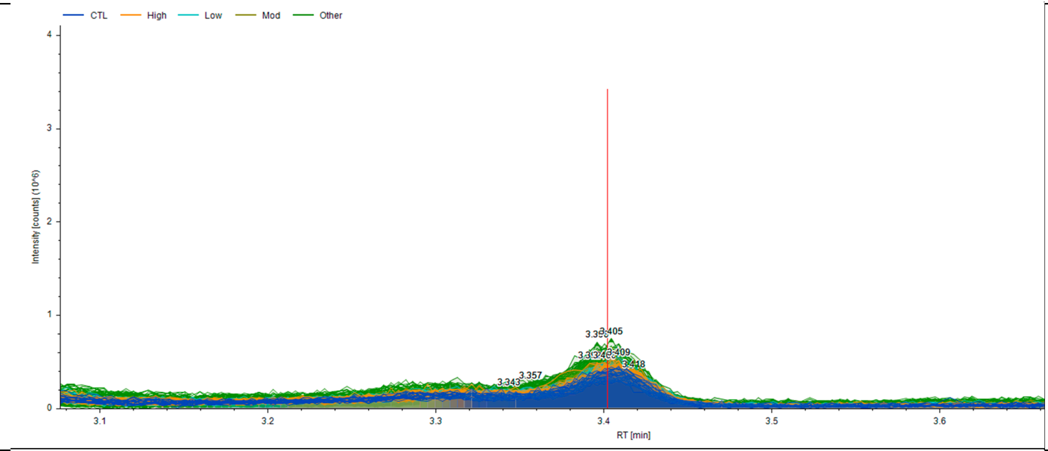

311.1464

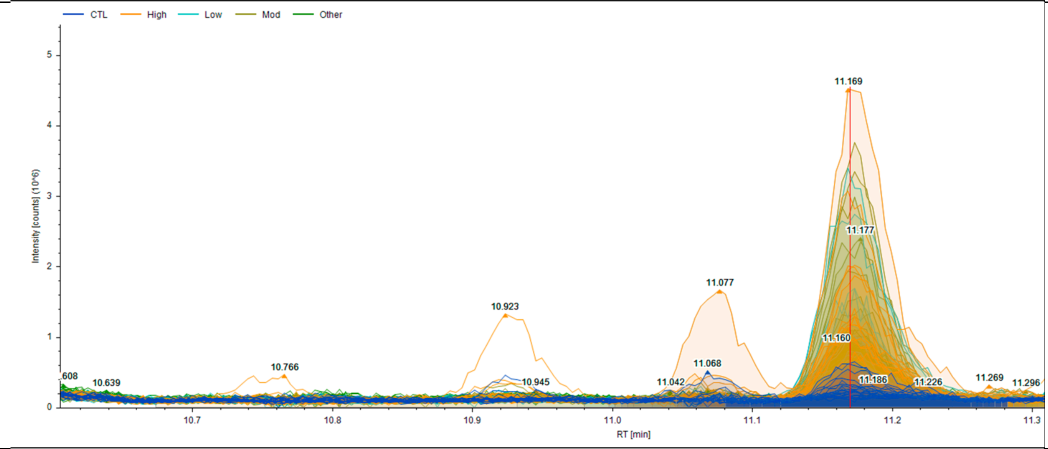

343.2241

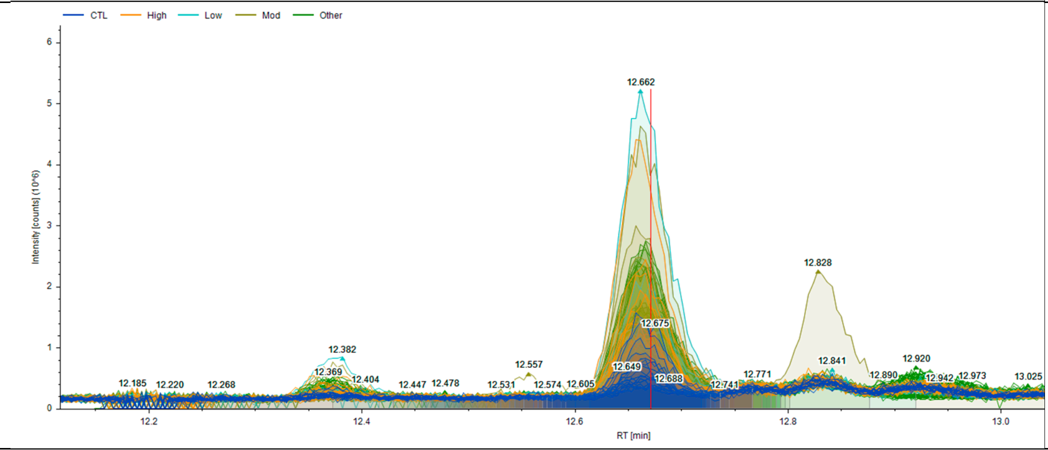

351.2504

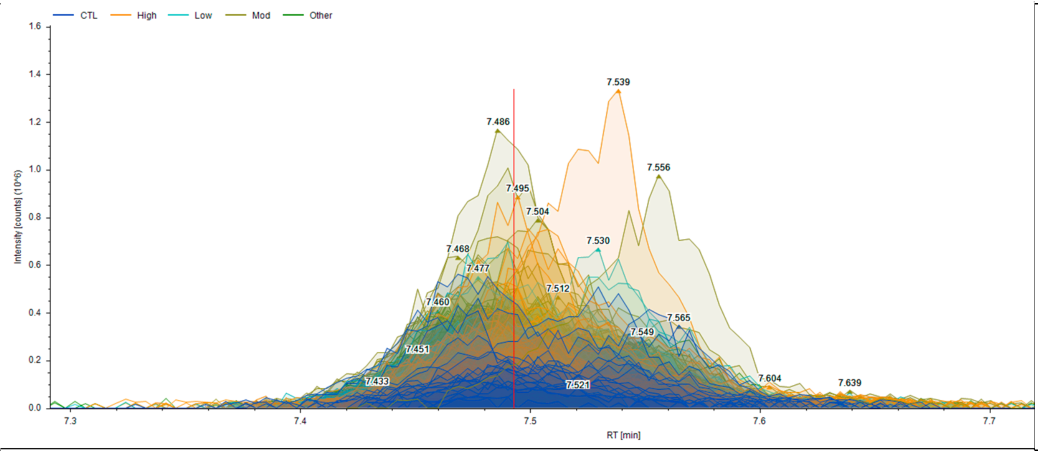

356.2793

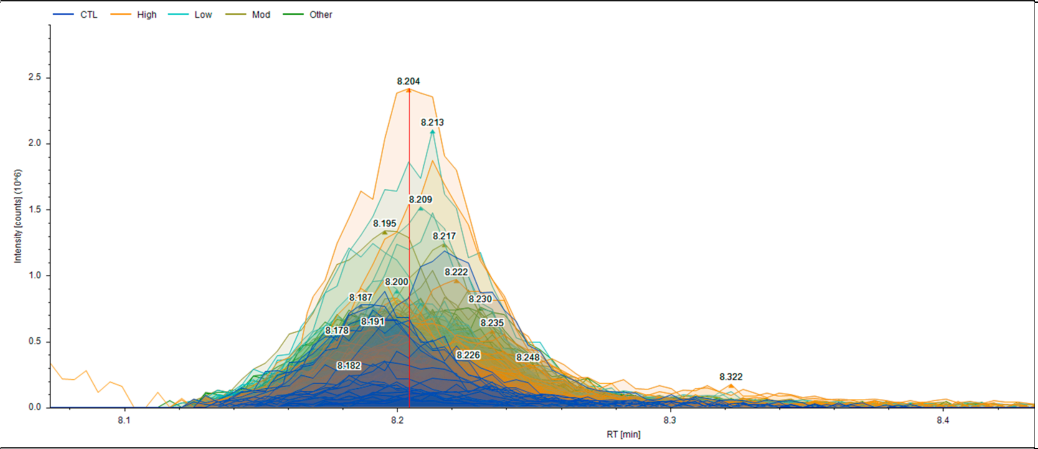

370.2950

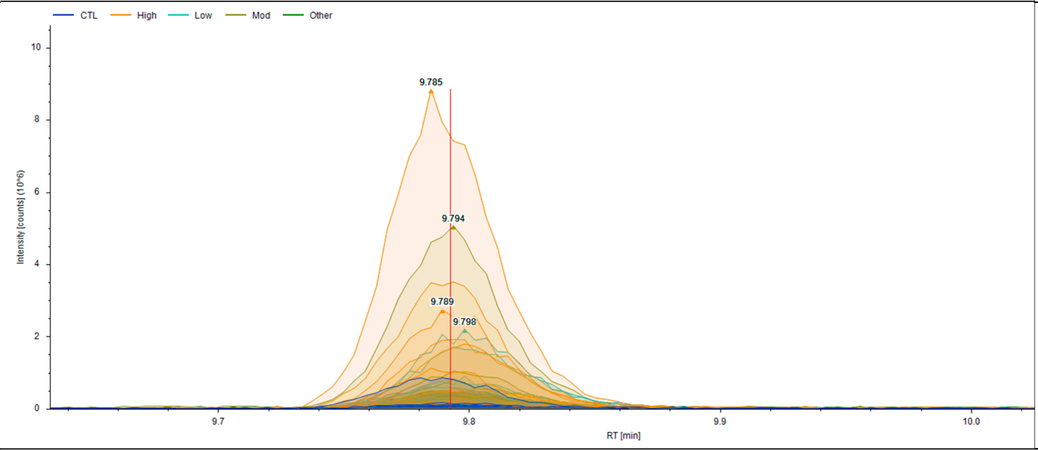

373.2735

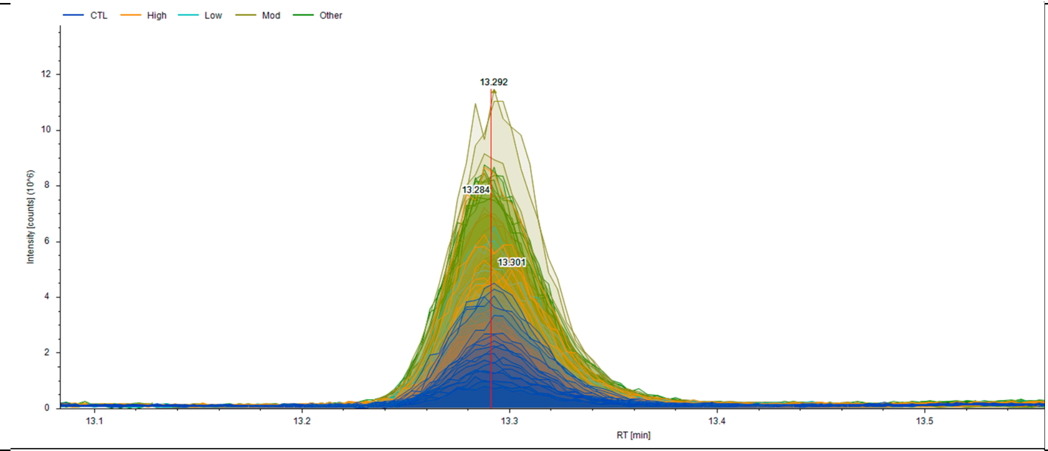	377.2659
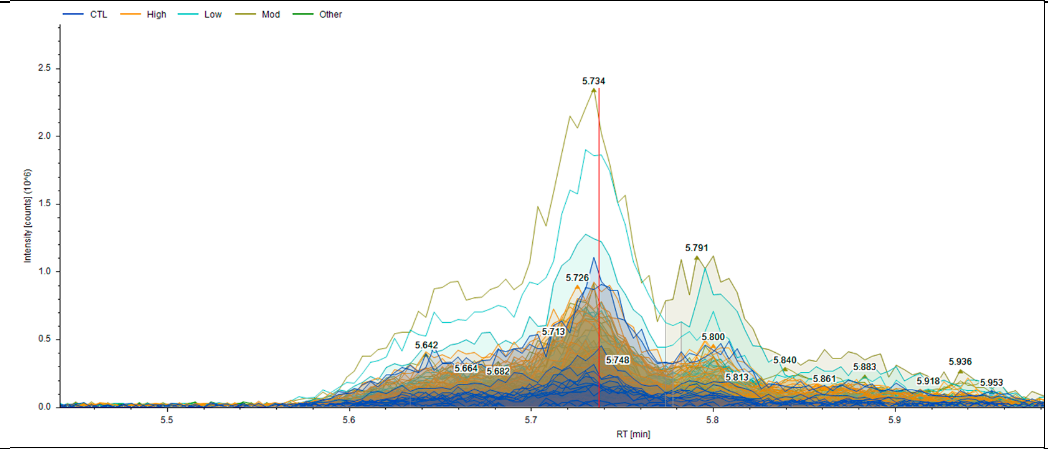	427.1938
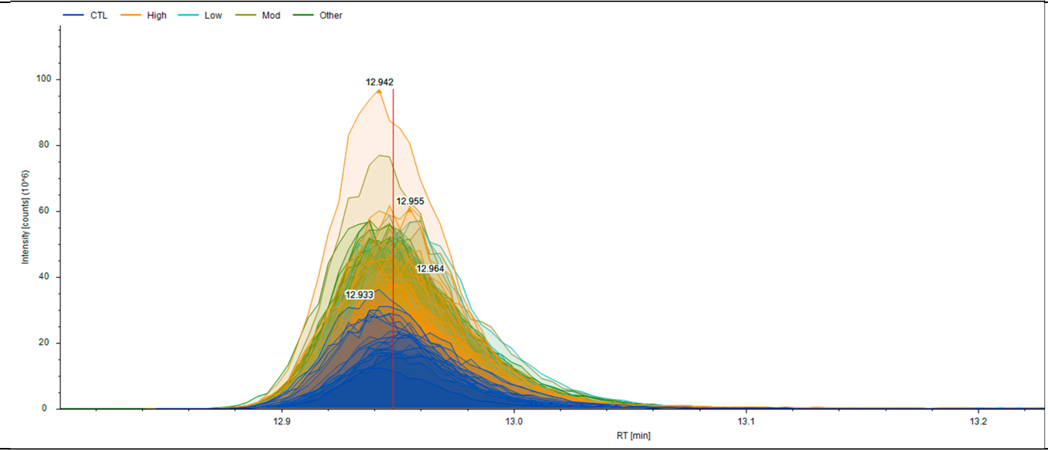	496.3397

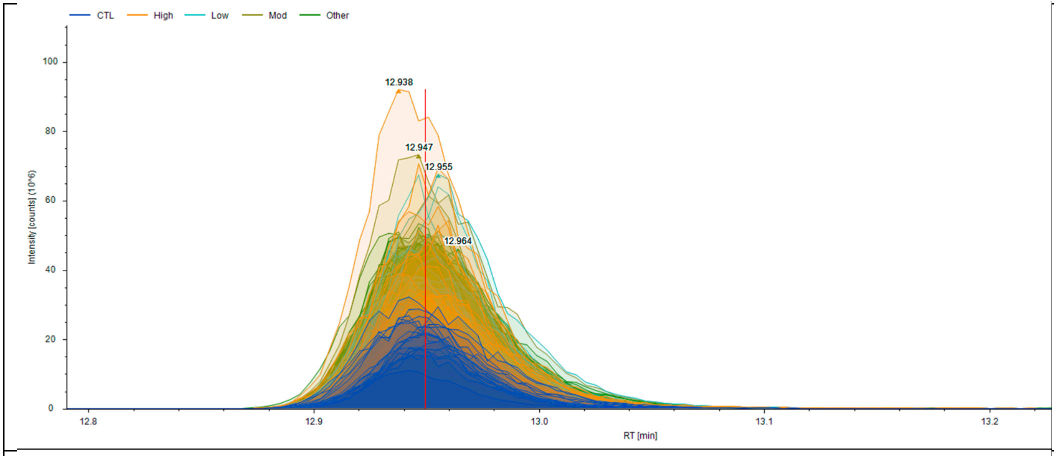	518.3215
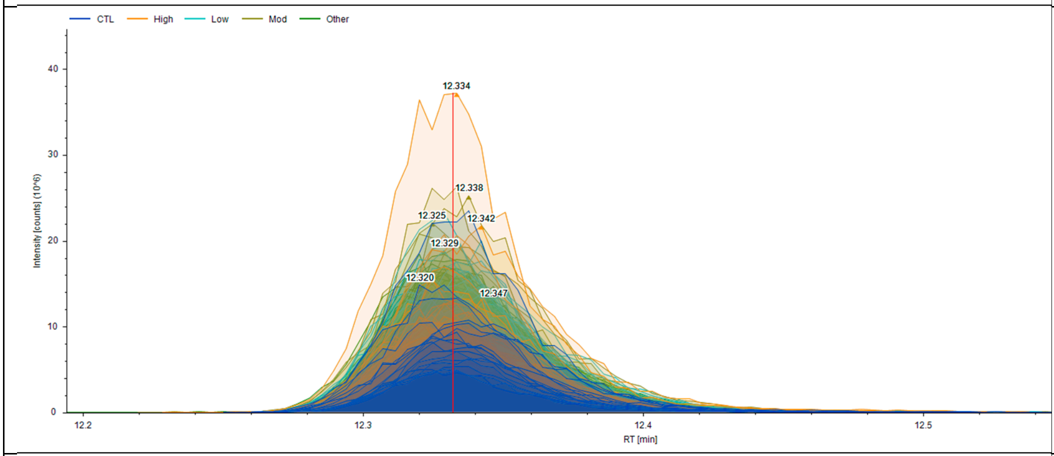	520.3398
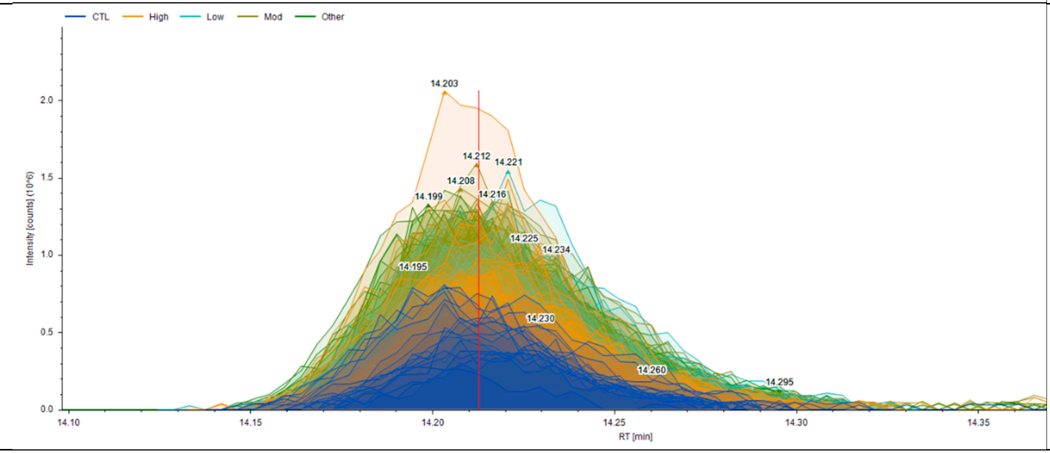	524.3710

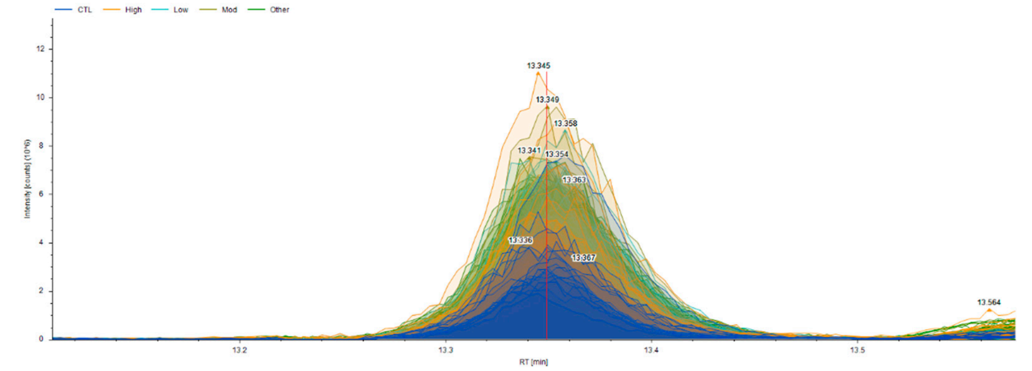	544.3373
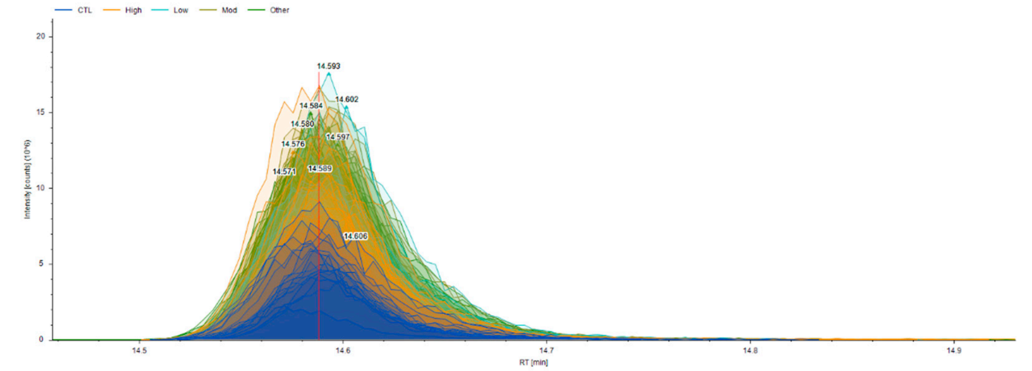	546.3528
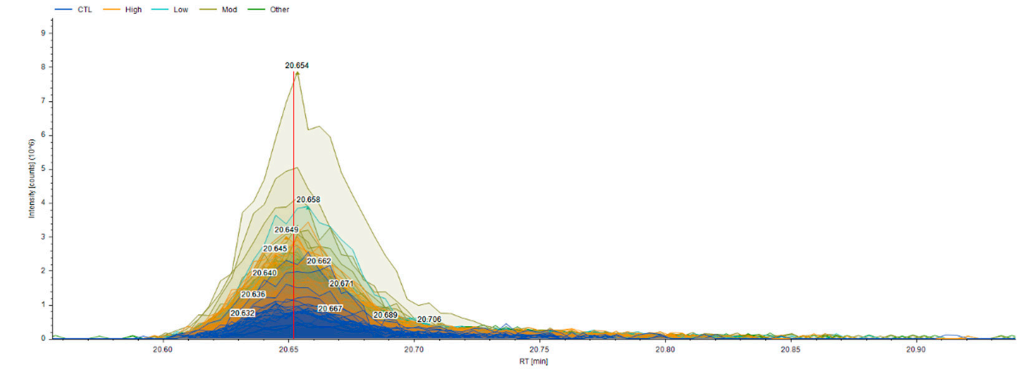	641.5110
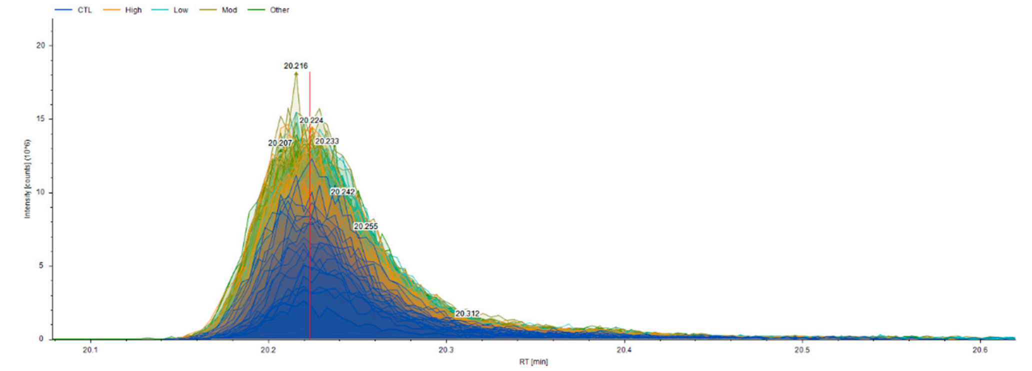	760.5845

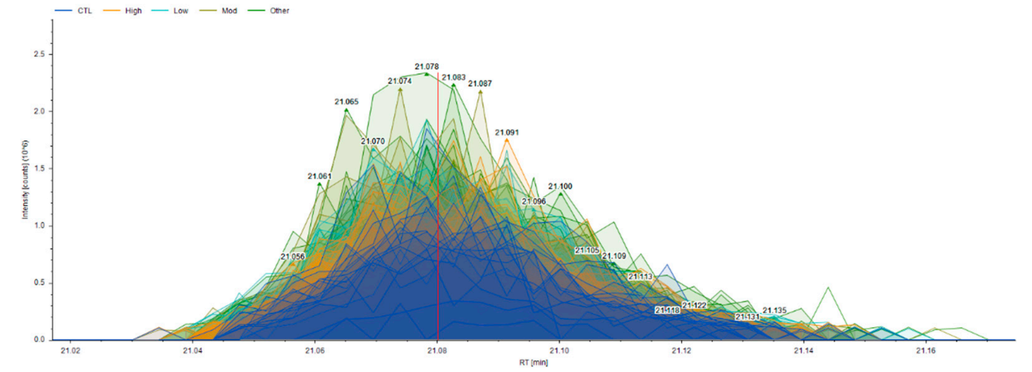	769.4224
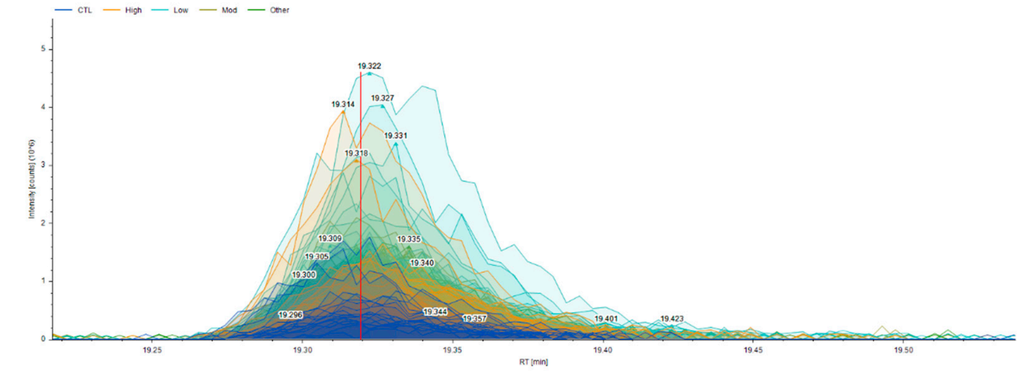	802.5350
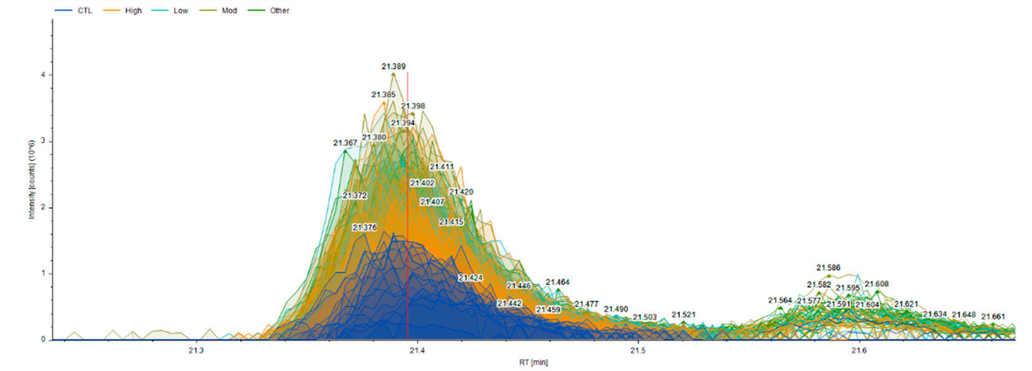	813.6839
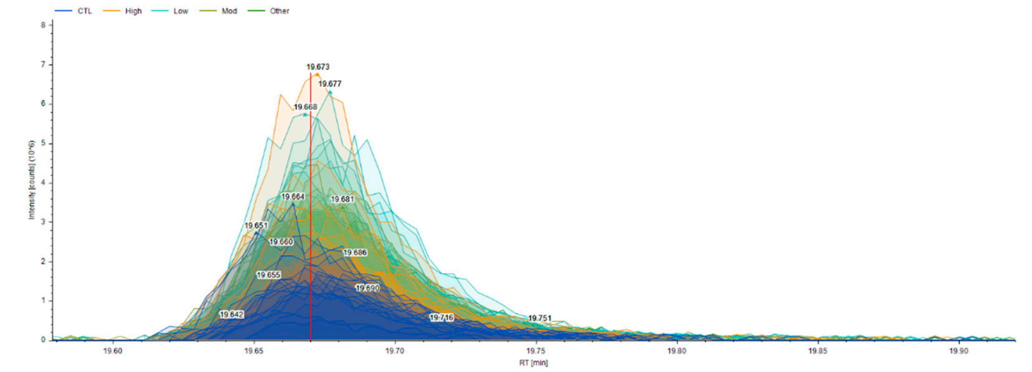	828.5507

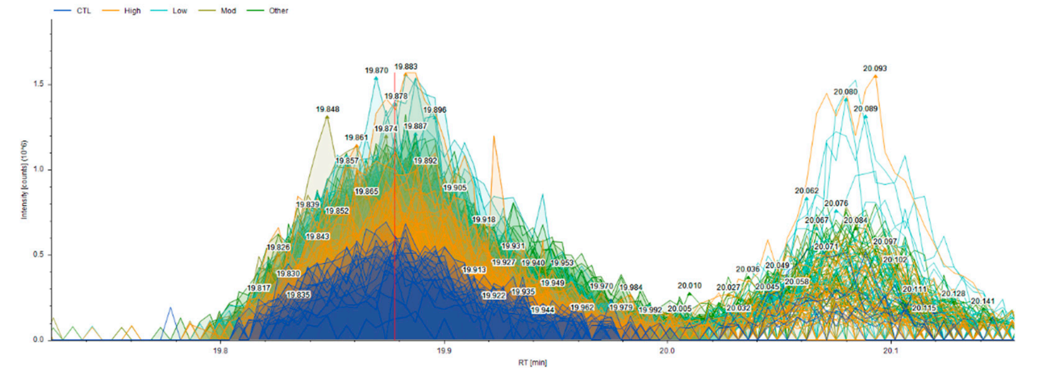	830.5664

Table S2: Peaks of summarized negative mode features

Feature Peak	m/z
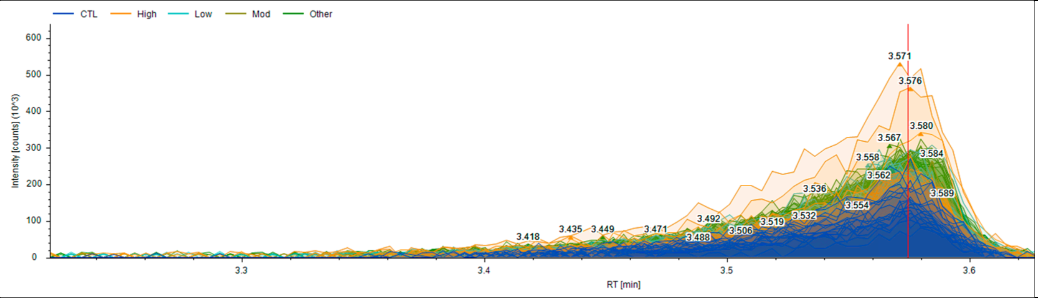	203.0830
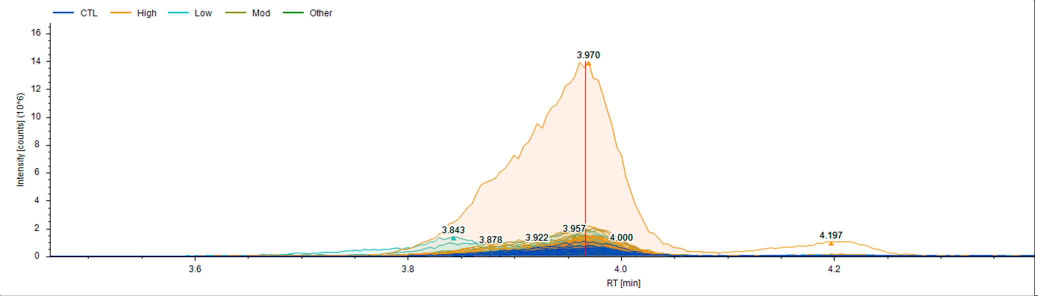	212.0026
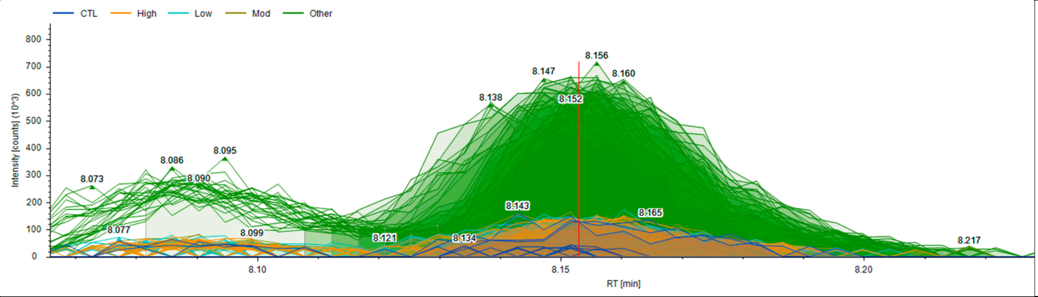	343.1707
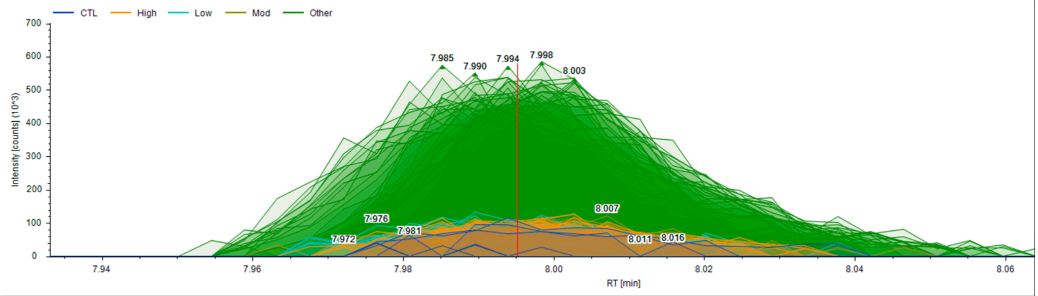	388.1559

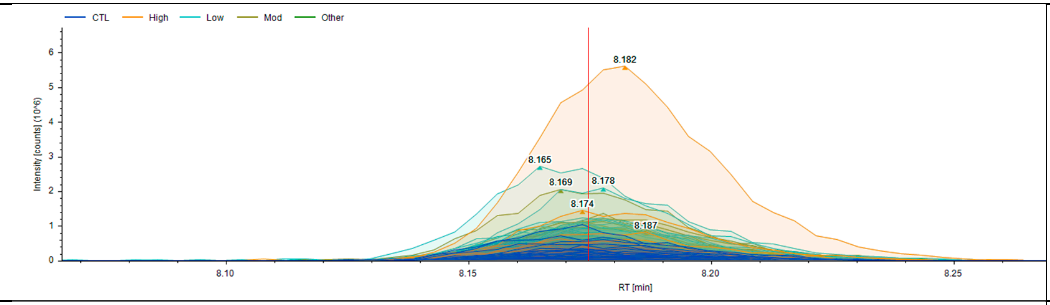	448.3073
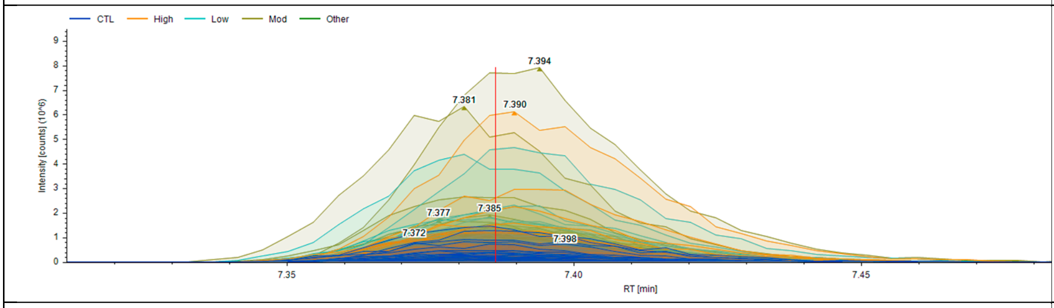	464.3020
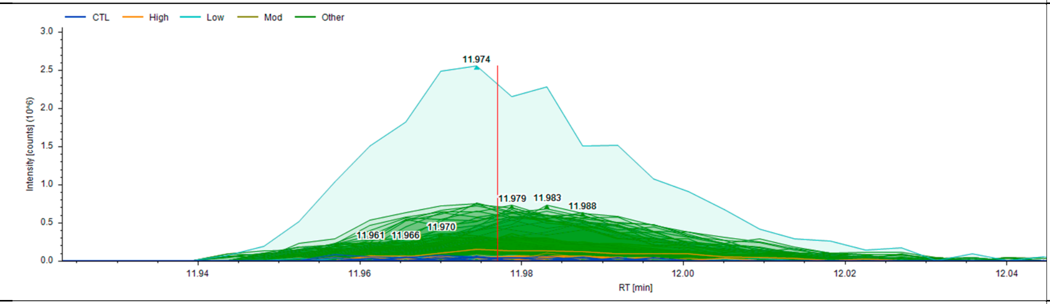	667.1419
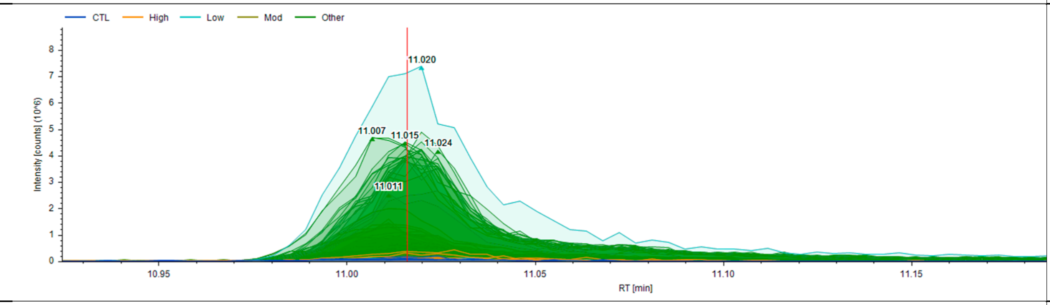	729.1788
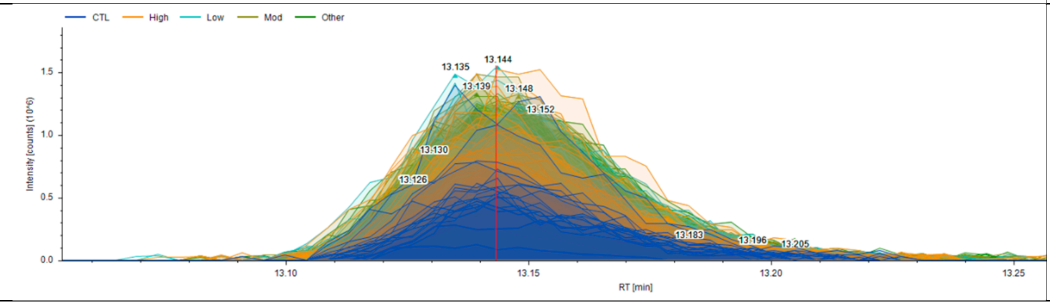	802.5614

Table S3: Complete List of Tentatively Identified Endogenous Metabolites in Low vs. High Models

m/z	RT	Tentative ID	Adduct	Monoisotopic Mass	Biological Significance
		17Z-hexacosenoic acid	$[M+NH_4]^+$	394.3811	Very long chain fatty acid
412.4147*	14.78	Ximenic acid	$[M+NH_4]^+$	394.3811	Very long chain fatty acid
		Cholylhistidine	$[M+H]^+$	545.3465	Bile acid-amino acid conjugates
		2-LysoPhosphatidylcholine	$[M+Na]^+$	523.3638	LysoPhosphatidylcholine
		LysoPC(0:0/18:0)	$[M+Na]^+$	523.3638	LysoPhosphatidylcholine
		LysoPC(20:3(8Z,11Z,14Z)/0:0)	$[M+H]^+$	545.3481	LysoPhosphatidylcholine
		LysoPC(20:3(5Z,8Z,11Z)/0:0)	$[M+H]^+$	545.3481	LysoPhosphatidylcholine
		LysoPC(18:0/0:0)	$[M+Na]^+$	523.3638	LysoPhosphatidylcholine
546.3528	14.59	Platelet-activating factor	$[M+Na]^+$	523.3638	Potent phospholipid activator and mediator of inflammation that has an important role in the pathogenesis of inflammatory disorders and cardiovascular disease
		LysoPC(0:0/20:4(5Z,8Z,11Z,14Z))	$[M+H]^+$	543.3325	LysoPhosphatidylcholine
		LysoPC(0:0/18:1(9Z))	$[M+Na]^+$	521.3481	LysoPhosphatidylcholine
		LysoPC(20:4(8Z,11Z,14Z,17Z)/0:0)	$[M+H]^+$	543.3325	LysoPhosphatidylcholine
		LysoPC(20:4(5Z,8Z,11Z,14Z)/0:0)	$[M+H]^+$	543.3325	LysoPhosphatidylcholine
		LysoPC(18:1(11Z)/0:0)	$[M+Na]^+$	521.3481	LysoPhosphatidylcholine
544.3373	13.35	LysoPC(18:1(9Z)/0:0)	$[M+Na]^+$	521.3481	LysoPhosphatidylcholine
116.0708	0.60	Proline	$[M+H]^+$	115.0633	Amino acid
		dermatan L-iduronate	$[M+NH_4]^+$	409.1584	N-acyl-alpha-hexosamines
427.1938	5.74	Chondroitin D-glucuronate	$[M+NH_4]^+$	409.1584	N-acyl-alpha-hexosamines
496.3397	12.95	LysoPC(0:0/16:0)	$[M+H]^+$	495.3325	LysoPhosphatidylcholine

		1-PalmitoylPhosphatidylcholine	$[M+H]^+$	495.3325	PalmitoylPhosphatidylcholine
		23-Acetoxysoladulcidine	$[M+Na]^+$	473.3505	Steroidal alkaloid
		LysoPC(16:0/0:0)	$[M+H]^+$	495.3325	LysoPhosphatidylcholine
		Clupanodonyl carnitine	$[M+Na]^+$	473.3505	Acylcarnitine
		Docosa-4,7,10,13,16-pentaenoyl carnitine	$[M+Na]^+$	473.3505	Acylcarnitine
373.2735	9.79	Cervonoyl ethanolamide	$[M+H]^+$	372.2664	N-aclethanolamines – A class of lipid compounds as constituents of membrane-bound phospholipid N-acylphosphatidylethanolamine (NAPE). Released from NAPE in response to stimuli and attributed to neurotransmission, membrane protection, immunomodulation
520.3398	12.33	LysoPC(0:0/18:2(9Z,12Z))	$[M+H]^+$	519.3325	LysoPhosphatidylcholine
		LysoPC(18:2(9Z,12Z)/0:0)	$[M+H]^+$	519.3325	LysoPhosphatidylcholine
370.2950	8.20	N-Oleoyl-L-Serine	$[M+H]^+$	369.2879	acyl-l-alpha-amino acids – Secondary metabolite for defense, signalling or from incomplete metabolism
		Myristoleoylcarnitine	$[M+H]^+$	369.2879	Acylcarnitines
		trans-2-Tetradecenoylcarnitine	$[M+H]^+$	369.2879	Acylcarnitines
		MG(18:3(9Z,12Z,15Z)/0:0/0:0)	$[M+NH_4]^+$	352.2614	Monoacylglyceride
		MG(18:3(6Z,9Z,12Z)/0:0/0:0)	$[M+NH_4]^+$	352.2614	Monoacylglyceride
		MG(0:0/18:3(6Z,9Z,12Z)/0:0)	$[M+NH_4]^+$	352.2614	Monoacylglyceride
		MG(0:0/18:3(9Z,12Z,15Z)/0:0)	$[M+NH_4]^+$	352.2614	Monoacylglyceride
		cis-5-Tetradecenoylcarnitine	$[M+H]^+$	369.2879	long-chain fatty acids
289.1409	5.12	pentadeca-3,5,7-trienedioic acid	$[M+Na]^+$	266.1518	Acylcarnitine
		pentadeca-5,7,9-trienedioic acid	$[M+Na]^+$	266.1518	Acylcarnitine

		(2E,6E,10E)-pentadeca-2,6,10-trienedioic acid	$[M+Na]^+$	266.1518	Acylcarnitine
		pentadeca-4,7,10-trienedioic acid	$[M+Na]^+$	266.1518	Acylcarnitine
		pentadeca-4,6,8-trienedioic acid	$[M+Na]^+$	266.1518	Acylcarnitine
		pentadeca-2,5,8-trienedioic acid	$[M+Na]^+$	266.1518	Acylcarnitine
		pentadeca-3,6,9-trienedioic acid	$[M+Na]^+$	266.1518	Acylcarnitine
		pentadeca-2,4,6-trienedioic acid	$[M+Na]^+$	266.1518	Acylcarnitine
		(3E,5Z,11Z)-pentadeca-3,5,11-trienedioic acid	$[M+Na]^+$	266.1518	Acylcarnitine
		15-Hydroperoxyeicosa-8Z,11Z,13E-trienoate	$[M+NH_4]^+$	338.2457	Hydroperoxyeicosatrienoic
		6,7-dihydro-12-epi-LTB4	$[M+NH_4]^+$	338.2457	Eicosanoid
		10,11-dihydro-leukotriene B4	$[M+NH_4]^+$	338.2457	Metabolite of lipid omega-oxidation of leukotriene B4 (LTB4). LTB4 is the major metabolite in neutrophil polymorphonuclear leukocytes
		12-Keto-tetrahydro-leukotriene B4	$[M+NH_4]^+$	338.2457	Eicosanoid
		5,6-DHET	$[M+NH_4]^+$	338.2457	Epoxide intermediate in the oxygenation of arachidonic acid by hepatic monooxygenases pathway
		11,12-DiHETrE	$[M+NH_4]^+$	338.2457	Cytochrome P450 (P450) Eicosanoid
		8,9-DiHETrE	$[M+NH_4]^+$	338.2457	Cytochrome P450 (P450) Eicosanoid
356.2793	7.49	14,15-DiHETrE	$[M+NH_4]^+$	338.2457	Cytochrome P450 (P450) Eicosanoid
215.1279	4.78	undec-3-enedioic acid	$[M+H]^+$	214.1205	Medium-chain fatty acids

		undec-4-enedioic acid	$[M+H]^+$	214.1205	Medium-chain fatty acids
		(2E)-undec-2-enedioic acid	$[M+H]^+$	214.1205	Medium-chain fatty acids
		undec-5-enedioic acid	$[M+H]^+$	214.1205	Medium-chain fatty acids
		(3E)-pent-3-enoic acid	$[M+NH_4]^+$	100.0524	Straight chain fatty acids
		3-Methylbut-3-enoic acid	$[M+NH_4]^+$	100.0524	Methyl-branched fatty acids
		N-Methyl-4-aminobutyric acid	$[M+H]^+$	117.0790	Gamma-amino acid/derivative
		2-Pentenoic acid	$[M+NH_4]^+$	100.0524	Straight chain fatty acids
		4-Pentenoic acid	$[M+NH_4]^+$	100.0524	Straight chain fatty acids
		D-Valine	$[M+H]^+$	117.0790	Valine and derivatives
		Senecioic acid	$[M+NH_4]^+$	100.0524	Methyl-branched fatty acids – Found in mealy bug species, Madeira cockroach, and Southern long-nosed bats. Also appears in urine of patients with 3-Methylcrotonic aciduria caused by 3-hydroxy-3-methylglutaryl-CoA lyase deficiency
		Betaine	$[M+H]^+$	118.0868	Trimethylated amino acid – Found in beet. Associated with metabolic syndrome, lipid disorders, and diabetes, and may have a role in vascular and other diseases. Betaine has been used in the treatment of liver disorders, for hyperkalemia, for homocystinuria, for hypochlorhydria, and for gastrointestinal disturbances
		L-Valine	$[M+H]^+$	117.0790	Alpha amino acid
118.0865	0.59	5-Aminopentanoic acid	$[M+H]^+$	117.0790	Lysine degradation product that can also indicate bacterial overgrowth or endogenous tissue necrosis. Normal metabolite in saliva. Function as weak inhibitor of blood clotting pathway and as weak GABA agonist.
668.5443	8.65	DG(20:3(8Z,11Z,14Z)-2OH(5,6)/0:0/i-16:0)	$[M+NH_4]^+$	650.5122	Diacylglycerols- glycerolipids containing a common glycerol backbone with at least one

					fatty acyl group esterified. Involved in the phospholipid metabolic pathway
		DG(i-16:0/0:0/20:3(8Z,11Z,14Z)-2OH(5,6))	$[M+NH_4]^+$	650.5122	Diacylglycerols- glycerolipids containing a common glycerol backbone with at least one fatty acyl group esterified. Involved in the phospholipid metabolic pathway
		DG(20:3(8Z,11Z,14Z)-2OH(5,6)/i-16:0/0:0)	$[M+NH_4]^+$	650.5122	Diacylglycerols- glycerolipids containing a common glycerol backbone with at least one fatty acyl group esterified. Involved in the phospholipid metabolic pathway
		DG(i-16:0/20:3(8Z,11Z,14Z)-2OH(5,6)/0:0)	$[M+NH_4]^+$	650.5122	Diacylglycerols- glycerolipids containing a common glycerol backbone with at least one fatty acyl group esterified. Involved in the phospholipid metabolic pathway
		DG(20:3(8Z,11Z,14Z)-2OH(5,6)/0:0/16:0)	$[M+NH_4]^+$	650.5122	Diacylglycerols- glycerolipids containing a common glycerol backbone with at least one fatty acyl group esterified. Involved in the phospholipid metabolic pathway
		DG(16:0/0:0/20:3(8Z,11Z,14Z)-2OH(5,6))	$[M+NH_4]^+$	650.5122	Diacylglycerols- glycerolipids containing a common glycerol backbone with at least one fatty acyl group esterified. Involved in the phospholipid metabolic pathway
		DG(20:3(8Z,11Z,14Z)-2OH(5,6)/16:0/0:0)	$[M+NH_4]^+$	650.5122	Diacylglycerols- glycerolipids containing a common glycerol backbone with at least one fatty acyl group esterified. Involved in the phospholipid metabolic pathway
		DG(16:0/20:3(8Z,11Z,14Z)-2OH(5,6)/0:0)	$[M+NH_4]^+$	650.5122	Diacylglycerols- glycerolipids containing a common glycerol backbone with at least one fatty acyl group esterified. Involved in the phospholipid metabolic pathway
359.2190	9.40	Prostaglandin C1(1-)	$[M+Na]^+$	336.2301	Prostaglandin and related compounds

		Prostaglandin C1	$[M+Na]^+$	336.2301	Prostaglandin and related compounds
		12,20-DiHETE	$[M+Na]^+$	336.2301	Hydroxyeicosatetraenoic acids
		9-Deoxy-delta12-PGD2	$[M+Na]^+$	336.2301	Prostaglandin and related compounds
		6,7-dihydro-5-oxo-12-epi-LTB4	$[M+Na]^+$	336.2301	Product of Leukotriene B4 beta-oxidation
		10,11-dihydro-12-oxo-LTB4	$[M+Na]^+$	336.2301	Product of Leukotriene B4 beta-oxidation
		Prostaglandin B-1	$[M+Na]^+$	336.2301	Prostaglandin and related compounds
		Prostaglandin A-1	$[M+Na]^+$	336.2301	Prostaglandin and related compounds
		7-Acetylintermedine	$[M+NH_4]^+$	341.1838	Alkaloids and derivatives – Found in borage
		Acetyllycopsamine	$[M+NH_4]^+$	341.1838	Alkaloids and derivatives
		9alpha-(3-Methylbutanoyloxy)-4S-hydroxy-10(14)-oplopen-3-one	$[M+Na]^+$	336.2301	Sesquiterpenoids
		7-Acetyllycopsamine	$[M+NH_4]^+$	341.1838	Alkaloids and derivatives
		Methyl-[8]-gingerol	$[M+Na]^+$	336.2301	Dimethoxybenzenes – Found in ginger
		[9]-Gingerol	$[M+Na]^+$	336.2301	Gingerol – Found in ginger
		(14S)-14,15-Dihydroxy-8(17),13(16)-labdadien-19-oic acid	$[M+Na]^+$	336.2301	Diterpenoids
		ent-1(10)-Halimene-15,19-dioic acid	$[M+Na]^+$	336.2301	Carbocyclic fatty acids
		5-Hydroperoxyicosa-6,8,11,14-tetraenoic acid (5-HPETE)	$[M+Na]^+$	336.2301	Hydroperoxyeicosatetraenoic acids
		5-HPETE	$[M+Na]^+$	336.2301	Hydroperoxyeicosatetraenoic acids
		8,15-DiHETE	$[M+Na]^+$	336.2301	Double oxidation product of arachidonic acid via 15-lipoxygenase. Eosinophil chemotactic factor of anaphylaxis
		5,15-DiHETE	$[M+Na]^+$	336.2301	Hydroxyeicosatetraenoic acids
		17,18-DiHETE	$[M+Na]^+$	336.2301	Hydroxyeicosatetraenoic acids
		14,15-DiHETE	$[M+Na]^+$	336.2301	Hydroxyeicosatetraenoic acids

		12(S)-Leukotriene B4	$[M+Na]^+$	336.2301	Agonist of G-protein-coupled receptors for leukotriene B4, BLT1, and BLT2
		6-trans-12-epi-Leukotriene B4	$[M+Na]^+$	336.2301	Metabolite of lipid omega-oxidation of leukotriene B4
		6-trans-Leukotriene B4	$[M+Na]^+$	336.2301	Enzymatic metabolite of leukotriene B4. Increases after stimulation with calcium-ionophore in asthma patients compared to healthy controls
		15H-11,12-EETA	$[M+Na]^+$	336.2301	Epoxyeicosatrienoic acids
		8(S)-HPETE	$[M+Na]^+$	336.2301	Hydroperoxyeicosatetraenoic acids
		12(R)-HPETE	$[M+Na]^+$	336.2301	Hydroperoxyeicosatetraenoic acids – Involved in conversion of arachidonic acid to 12(R)-HETE
		11H-14,15-EETA	$[M+Na]^+$	336.2301	Epxoyeicosatrienoic acids
		11(R)-HPETE	$[M+Na]^+$	336.2301	Hydroperoxyeicosatetraenoic acids – Formed from arachidonic acid in prostaglandin endoperoxide H synthase-1 cyclooxygenase site
		Hepoxilin B3	$[M+Na]^+$	336.2301	Normal human epidermis Eicosanoid – Dramatically elevated in psoriatic lesions.
		Hepoxilin A3	$[M+Na]^+$	336.2301	Electrophilic Eicosanoids synthesized during arachidonic acid oxidative metabolism
		12(S)-HPETE	$[M+Na]^+$	336.2301	Monohydroperoxy fatty acids produced by non-enzymatic oxidation of arachidonic acids.
		15(S)-HPETE	$[M+Na]^+$	336.2301	Hydroperoxyeicosatetraenoic acid – Corresponding hydroperoxide of 15(S)-HETE. Initiates apoptosis in vascular smooth muscle cells. Lipoxygenase metabolite that affect expression of cell adhesion molecules involved in adhesion of leukocytes and/or accumulation of leukocytes in the vascular

					endothelium (Initial events in endothelial cell injury). Enhances activity of enzymes lipoxygenase and Na+,K+-ATPase of brain microvessels
		Prostaglandin B1	$[M+Na]^+$	336.2301	Metabolite of PGE1. Inhibit arachidonic acid mobilization in human neutrophils and endothelial cells. Enhances peripheral vascular resistance and elevate blood pressure. Blocks S-phase DNA synthesis
		Prostaglandin A1	$[M+Na]^+$	336.2301	Prostaglandin characterized by cyclopentenone structure. Antiviral ability, induce heat shock protein synthesis, tumor suppressant.
		8-iso-PGA1	$[M+Na]^+$	336.2301	Isoprostane – Produced by peroxidative attack of membrane lipids. Associated with asthma, hypertension, ischemia reperfusion injury. Marker of oxidative stress
		5(S)-Hydroperoxyeicosatetraenoic acid	$[M+Na]^+$	336.2301	Lipid hydroperoxide precursor of leukotrienes. Produced from arachidonic acid. Implicated with cancer, cardiovascular disease, neurodegeneration
		Leukotriene B4	$[M+Na]^+$	336.2301	Major metabolite in neutrophil polymorphonuclear leukocytes. Pro-inflammation. Recruits and activates neutrophils, monocytes, eosinophils. Stimulates production of proinflammatory cytokines and mediators

Note: * = Clear peak, but peak shape is subpar

Table S4: Complete List of Tentatively Identified Exogenous Metabolites in Low vs. High Models

m/z	RT	Tentative ID	Adduct	Monoisotopic Mass	Biological Significance
603.2990	12.95	Hordatine B	$[M+Na]^+$	580.3122	2-arylbenzofuran flavonoids – Found in barley and cereals
412.4147	14.78	trans-2-Hexacosenoic acid	$[M+NH_4]^+$	394.3811	Very long chain fatty acids
		10,12-Hexacosanedione	$[M+NH_4]^+$	394.3811	Beta-diketones - Found in fats and oil
		6,8-Hexacosanedione	$[M+NH_4]^+$	394.3811	Beta-diketones - Found in fats and oil
		5,7-Hexacosanedione	$[M+NH_4]^+$	394.3811	Beta-diketones - Found in fats and oil
		11,13-Hexacosanedione	$[M+NH_4]^+$	394.3811	Beta-diketones - Found in fats and oil
		4,6-Hexacosanedione	$[M+NH_4]^+$	394.3811	Beta-diketones - Found in fats and oil
		7,9-Hexacosanedione	$[M+NH_4]^+$	394.3811	Beta-diketones - Found in fats and oil
245.1146	5.61	3,5-Diisopropylsalicylic acid	$[M+Na]^+$	222.1256	Salicylic acids
		3-Hexylsalicylic acid	$[M+Na]^+$	222.1256	Salicylic acids
		(+)-Dehydrovomifoliol	$[M+Na]^+$	222.1256	Sesquiterpenoids – Found in rice
		Hexyl salicylic acid	$[M+Na]^+$	222.1256	O-hydroxybenzoic acid esters
		3-Hydroxyibuprofen	$[M+Na]^+$	222.1256	Phenylpropanoic acids – Metabolite of ibuprofen, a nonsteroidal anti-inflammatory drug for relief of arthritis, fever, and pain
		2-Hydroxyibuprofen	$[M+Na]^+$	222.1256	Phenylpropanoic acids – Metabolite of ibuprofen, a nonsteroidal anti-inflammatory drug for relief of arthritis, fever, and pain
		1-Hydroxyibuprofen	$[M+Na]^+$	222.1256	Phenylpropanoic acids – Metabolite of ibuprofen, a nonsteroidal anti-inflammatory drug for relief of arthritis, fever, and pain
		Methyl 3-epi-4,5-didehydrojasmonate	$[M+Na]^+$	222.1256	Methyl esters

		Annuionone B	$[M+Na]^+$	222.1256	Oxepanes – Found in fats, oils, sunflowers
		Isoamyl p-anisate	$[M+Na]^+$	222.1256	P-methoxybenzoic acids and derivatives
		Dehydrovomifoliol	$[M+Na]^+$	222.1256	Sesquiterpenoids
321.1307	4.55	Piribedil	$[M+Na]^+$	298.1430	N-arylpiperiazines
		(2S,3S,4R,5R)-5-(6-Aminopurin-9-yl)-N-cyclopropyl-3,4-dihydroxyoxolane-2-carboxamide	$[M+H]^+$	320.1233	(AKA CPCA) Purine nucleoside -
		(2S,3S,4S,5S)-2-(5-Amino-7-methyl-2,6,7,9,11-pentazatricyclo[6.3.1.04,12]dodeca-1(12),3,5,8,10-pentaen-2-yl)-5-(hydroxymethyl)oxolane-3,4-diol	$[M+H]^+$	320.1233	Glycosylamines
		D-Fructosazine	$[M+H]^+$	320.1220	Pyrazines
		Gynocardin	$[M+NH_4]^+$	303.0954	Cyanogenic glycosides – Found
		3,7,8,15-Scirpenetetrol	$[M+Na]^+$	298.1416	Trichothecenes
		Toxin T2 tetrol	$[M+Na]^+$	298.1416	Type-a Trichothecene mycotoin produced by Fusarium species. Associated with weight loss, diarrhea, lethargy, low plasma glucose, decreases in blood cell and leukocyte count. damage to cartilaginous tissues, and induction of apoptosis
		Isomugineic acid	$[M+H]^+$	320.1220	Gamma amino acids and derivatives – Found in barleys, and cereal
		Nialamide	$[M+Na]^+$	298.1430	Pyridinecarboxylic acids and derivatives – Non-selective, irreversible monoamine oxidase inhibitor of hydrazine class that was

					used as antidepressant. Withdrawn due to risk of hepatotoxicity
544.3373	13.35	(S)-1-(Sec-Butyl)-N-((4,6-dimethyl-2-oxo-1,2-dihydropyridin-3-yl)methyl)-3-methyl-6-(6-(piperazin-1-yl)pyridin-3-yl)-1H-indole-4-carboxamide	$[M+NH_4]^+$	526.3056245	Pyridinylpiperazines
		Luminol	$[M+NH_4]^+$	177.0538	Phthalazinones
		1,3,9-Trimethylxanthine	$[M+H]^+$	194.0804	(AKA Isocaffeine) Xanthines
		3-[(2-Mercapto-1-methylpropyl)thio]-2-butanol	$[M+H]^+$	194.0799	Secondary alcohols
		4-Aminophthalhydrazide	$[M+NH_4]^+$	177.0538	Phthalazinones
		Enprofylline	$[M+H]^+$	194.0804	Xanthines – Drug for managing asthma, peripheral vascular disease, cerebrovascular insufficiency, sickle cell disease, and diabetic neuropathy
195.0876	3.38	Caffeine	$[M+H]^+$	194.0804	Methyl xanthine alkaloid or purine.
		N-Allylglycine	$[M+H]^+$	115.0633	Alpha amino acids
		2-Pentenedial	$[M+NH_4]^+$	98.0368	Enals
		5-Hydroxy-1-methylpyrrolidin-2-one	$[M+H]^+$	115.0633	N-alkylpyrrolidines
		N-(2-Hydroxyethyl)acrylamide	$[M+H]^+$	115.0633	(AKA PNHEA or polyduramide) n-acylethanolamines
		5-Methyl-3(2H)-furanone	$[M+NH_4]^+$	98.0368	Furanones
		alpha-Methylene-gamma-butyrolactone	$[M+NH_4]^+$	98.0368	Gamma butyrolactones
		DL-Allylglycine	$[M+H]^+$	115.0633	Alpha amino acids
116.0708	0.60	Acetamidopropanal	$[M+H]^+$	115.0633	Acetamidopropanal is associated with urea cycle and metabolism of arginine, proline, glutamate, aspartate and asparagine

		2-Furanmethanol	$[M+NH_4]^+$	98.0368	Heteroaromatic compounds – Found in cereals, potato, white mustards, arabica coffee, cocoa
		5-Methyl-2(3H)-furanone	$[M+NH_4]^+$	98.0368	Butenolides – Found in blackberries
		Pterolactam	$[M+H]^+$	115.0633	Pyrrolidines – Found in green vegetables and root vegetables
		5-Hydroxy-4-pentenoic acid d-lactone	$[M+NH_4]^+$	98.0368	Dihydropyranones – Found in nuts
		4-Amino-2-methylenebutanoic acid	$[M+H]^+$	115.0633	Gamma amino acids and derivatives
		Mespirenone	$[M+H]^+$	426.1865	Spironolactones and derivatives – Steroid lactones
		Queuosineueosine	$[M+NH_4]^+$	409.1597	Pyrrolopyrimidine nucleosides and nucleotides. Found in bacteria
		Pisumionoside	$[M+Na]^+$	404.2046	Pisumionoside
		Linustatin	$[M+NH_4]^+$	409.1584	Cyanogenic glycosides – Found in beans, pear shea tree, longan
		Queuosine	$[M+NH_4]^+$	409.1597	Modified nucleoside present in tRNAs in bacteria and eukaryotes
427.1938	5.74	Flunarizine	$[M+Na]^+$	404.2064	Diphenylmethanes – Drug for prophylaxis of migraine, occlusive peripheral vascular disease, vertigo of central and peripheral origin, and adjuvant in therapy of epilepsy. Involved in flunarizine h1-antihistamine action pathway
		Cesamet	$[M+H]^+$	372.2664	(AKA Nabilone) Naphthopyranones
373.2735	9.79	(3R, 6'Z)-3,4-Dihydro-8-hydroxy-3-(6-pentadecenyl)-1H-2-benzopyran-1-one	$[M+H]^+$	372.2664	2-benzopyrans
448.3057	8.77	Piritramide	$[M+NH_4]^+$	430.2733	Synthetic opioid analgesic used for treatment of postoperative pain

		2-(2-Methylbutanoyl)-9-(3-methyl-2E-pentenoyl)-2b,9a-dihydroxy-4Z,10(14)-oplopadien-3-one	$[M+NH_4]^+$	430.2719	Sesquiterpenoids
338.0165	8.26	Efavirenz	$[M+Na]^+$	315.0274	Benzoxazines – antiretroviral drug for preventing HIV transmission and treating HIV type 1. Non-nucleoside reverse transcriptase inhibitor principally metabolized by cytochrome P450 system to hydroxylated metabolites
159.1168	5.53	1-Phenyl-1-cyclohexene	$[M+H]^+$	158.1096	Benzene and substituted derivatives
355.2630	9.79	1,1'-[1,12-Dodecanediylbis(oxy)]bisbenzene	$[M+H]^+$	354.2559	Phenol ethers – Found in nuts
		3-Ethyl-4-(9-hydroxy-4,6,8,10-tetramethyl-7-oxododec-4-en-2-yl)oxetan-2-one	$[M+NH_4]^+$	352.2614	Terpene lactones
370.2950	8.20	Tetradecenoylcarnitine	$[M+H]^+$	369.2879	long-chain fatty acids
		Zaprinast	$[M+NH_4]^+$	271.1069	Triazolopyrimidines
		Deoxyqinghaosu	$[M+Na]^+$	266.1518	Dioxolopyrans
		Deoxyartemsinin	$[M+Na]^+$	266.1518	Metabolite of Artemisinin, a drug against Plasmodium falciparum malaria
		Sporol	$[M+Na]^+$	266.1518	1,3-dioxepanes
		1alpha,15-Dihydroxymarasmene	$[M+Na]^+$	266.1518	Furofurans
		3alpha,15-Dihydroxymarasmene	$[M+Na]^+$	266.1518	Furofurans – Found in mushrooms
		Ipomeamaronol	$[M+Na]^+$	266.1518	Fatty alcohols
		10-Hydroxymyoporone	$[M+Na]^+$	266.1518	Fatty alcohols
		Adhumulinic acid	$[M+Na]^+$	266.1518	Beta-diketones – Found in alcoholic beverages
		Ipomeamaronolide	$[M+Na]^+$	266.1518	Butenolides – Found in potato, root vegetables
289.1409	5.12	7-Hydroxytrichodermol	$[M+Na]^+$	266.1518	Gamma-butyrolactones

		Isoleptospermone	$[M+Na]^+$	266.1518	Monocyclic monoterpenoids
		[4]-Gingerol	$[M+Na]^+$	266.1518	Gingerol – Found in ginger
		[4]-Isogingerol	$[M+Na]^+$	266.1518	Methoxyphenols – Found in ginger
		Alkhanol	$[M+Na]^+$	266.1518	Eudesmanolides
		4'-Dihydroabscisic acid	$[M+Na]^+$	266.1518	Absisic acids and derivatives
		Blennin D	$[M+Na]^+$	266.1518	Terpene lactones
		(1alpha,4alpha,5beta,6alpha,11betaH)-1,4-Epoxy-5-hydroxy-10(14)-germacren-12,6-olide	$[M+Na]^+$	266.1518	Gamma butyrolactones – Found in herbs and spices
		(6beta,8betaOH)-6,8-Dihydroxy-7(11)-eremophilen-12,8-olide	$[M+Na]^+$	266.1518	Terpene lactones
		(R)-Athanagrandione	$[M+Na]^+$	266.1518	Aryl alkyl ketones – Found in potatoes
		Absindiol	$[M+Na]^+$	266.1518	Guaianolides and derivatives
		3,14-Dihydroxy-11,13-dihydrocostunolide	$[M+Na]^+$	266.1518	Germacranolides and derivatives
		Arlatin	$[M+Na]^+$	266.1518	Guaianolides and derivatives
		1alpha-Hydroxyarbusculin A	$[M+Na]^+$	266.1518	Eudesmanolides, secoeudesmanolides, and derivatives
		Arabsin	$[M+Na]^+$	266.1518	Eudesmanolides, secoeudesmanolides, and derivatives
		Humulinic acid A	$[M+Na]^+$	266.1518	Vinylogous acids – Found in alcoholic beverages
		Artemin	$[M+Na]^+$	266.1518	Eudesmanolides, secoeudesmanolides, and derivatives
356.2793	7.49	11-Deoxy-PGE1	$[M+NH_4]^+$	338.2457	Prostaglandin and related compounds
		(+-)-Zoapatanol	$[M+NH_4]^+$	338.2457	Monocylic monoterpenoids
		Kirenol	$[M+NH_4]^+$	338.2457	Diterpenoids
		(3S,4S)-4-[(E,2S,6R,8S,9R,10R)-9-Hydroxy-4,6,8,10-tetramethyl-7-oxododec-4-en-2-yl]-3-methyloxetan-2-one	$[M+NH_4]^+$	338.2457	Terpene lactones

		3-Epiaphidicolin	$[M+NH_4]^+$	338.2457	Aphidicolane and stemodane diterpenoids
		5,8,11-Eicosatrienoic acid, 14,15-dihydroxy-, (5Z,8Z,11Z)-	$[M+NH_4]^+$	338.2457	Hydroxyeicosatrienoic acid
		Sterebin E	$[M+NH_4]^+$	338.2457	Diterpenoids
		Neoacrimarine F	$[M+NH_4]^+$	531.1529	Triacylglycerols
549.1857	16.38	Neoacrimarine I	$[M+NH_4]^+$	531.1529	Acridones – Found in citrus
235.0977	0.50	Burimamide	$[M+Na]^+$	212.1096	Imidazoles – Primary metabolite involved in growth, development, reproduction
674.2887	19.63	Leurubicin	$[M+NH_4]^+$	656.2581	Anthracyclines
		2-Carboxy-5,7-dimethyl-4-octanolide	$[M+H]^+$	214.1205	Gamma butyrolactones
		5-Hexyltetrahydro-2-oxo-3-furancarboxylic acid	$[M+H]^+$	214.1205	Gamma butyrolactones
215.1279	4.78	alpha-Carboxy-delta-decalactone	$[M+H]^+$	214.1205	Delta valerolactones
		Depropionylbezitramide	$[M+H]^+$	436.2263	Diphenylacetonitriles
437.2356	3.97	Caldiamide	$[M+NH_4]^+$	419.2016	Alpha amino acid amides
		Tert-Butyl carbamate	$[M+H]^+$	117.0790	Carbamate esters
		3-Amino-2,2-dimethylpropanoic acid	$[M+H]^+$	117.0790	Beta amino acids and derivatives
		2R-Hydroxymethyl-3S-hydroxypyrolidine	$[M+H]^+$	117.0790	Pyrrolidines
		3-methyl-4-cis-hydroxy-2-butenal	$[M+NH_4]^+$	100.0524	Enals – Found in fennel, sorrel, savoy cabbage, hickory nuts
		2-Butenoic acid methyl ester	$[M+NH_4]^+$	100.0524	Fatty acid esters – Found in papaya
		3-methyl-4-trans-hydroxy-2-butenal	$[M+NH_4]^+$	100.0524	Enals – Found in tamarind, persian lime, European chestnut, sweet bay
		5-Valerolactone	$[M+NH_4]^+$	100.0524	Delta valerolactones
118.0865	0.59	Norvaline	$[M+H]^+$	117.0790	Non-proteinogenic branched-chain amino acids – Found in E. coli and Bacillus subtilis

		DL-Norvaline	$[M+H]^+$	117.0790	Alpha amino acids – Found in anatidaes, chickens, buckwheats, pigs
		Methyl methacrylate	$[M+NH_4]^+$	100.0524	Enoate esters – Found in blackberries
		Dihydro-3-methyl-2(3H)-furanone	$[M+NH_4]^+$	100.0524	Gamma butyrolactones – Found in fruits
		Angelic acid	$[M+NH_4]^+$	100.0524	Found in fats and oils, constituent of Angelica archangelica. Formerly used as sedative
		Dihydro-5-methyl-2(3H)-furanone	$[M+NH_4]^+$	100.0524	Gamma butyrolactones – Also considered fatty ester lipid
		2,4-Pentanedione	$[M+NH_4]^+$	100.0524	Beta-diketones – Found in blackberries and papaya
		2-Methyltetrahydrofuran-3-one	$[M+NH_4]^+$	100.0524	Furanones – Flavouring agent in coffee
		2,3-Pentanedione	$[M+NH_4]^+$	100.0524	Alpha-diketones – Found in coffee, tamarinds, cauliflowers, green beans, cereal
		Ethyl acrylate	$[M+NH_4]^+$	100.0524	Flavouring agent with potential carcinogenic properties
		3-Methylbutyrolactone	$[M+NH_4]^+$	100.0524	Gamma butyrolactones – Found in anatidaes, chickens, pigs, pulses
		N-Methyl-a-aminoisobutyric acid	$[M+H]^+$	117.0790	Alpha amino acids – Found in anatidaes, chickens, pigs. Is a secondary metabolite
		2-Ethylacrylic acid	$[M+NH_4]^+$	100.0524	Alpha, beta-unsaturated monocarboxylic acid. Associated with 3-oxoacyl-CoA thiolase deficiency and methylmalonyl-CoA mutase deficiency

		Tiglic acid	$[M+NH_4]^+$	100.0524	Monocarboxylic unsaturated organic acid – Found in croton oil and other natural products
		Glutaral	$[M+NH_4]^+$	100.0524	Antimicrobial agent in sugar mills and high fructose corn syrup
		Vaporole	$[M+H]^+$	117.0790	Antianginal agent that also cause direct vasorelaxation through vascular generation of Nitric oxide and relaxation via cyclic guanosine monophosphate-dependent process
		Isopropenyl acetate	$[M+NH_4]^+$	100.0524	Enol esters
		Amyl Nitrite	$[M+H]^+$	117.0790	Organic o-nitroso compounds – Drug for rapid relief of angina pectoris
		3-Methyl-alpha-pyrrolidinopropiophenone	$[M+NH_4]^+$	217.1467	Alkyl-phenylketones
		4'-Methyl-alpha-pyrrolidinopropiophenone	$[M+NH_4]^+$	217.1467	Alkyl-phenylketones
		1,2,3,4,5,6-Hexahydro-6,11-dimethyl-2,6-methano-3-benzazocin-8-ol	$[M+NH_4]^+$	217.1467	2,6-dimethyl-3-benzazocines
		1-Acetyl-2-(3-pyrrolidino-1-propynyl)piperidine	$[M+H]^+$	234.1732	N-acylpiperidines
		Ethoxyquin	$[M+NH_4]^+$	217.1467	Antioxidant used in animal feeds, preservation of colour in production of chili powder, paprika, ground chili. Formerly used as pesticide/herbicide. Used as post-harvest dip for apples and pears to prevent scald
		5-Methyl-2,5-di-1-pyrrolidinyl-2-cyclopenten-1-one	$[M+H]^+$	234.1732	N-alkylpyrrolidines
235.1803	2.98	Lidocaine	$[M+H]^+$	234.1732	Loal anesthetic and cardiac depressant used as antiarrhythmia agent
331.0938	7.14	1-Nitrohydroxyphenyl-N-benzoylalanine	$[M+H]^+$	330.0852	Tyrosine and derivatives

		Glyinflanin H	$[M+Na]^+$	308.1049	2-arylbenzofuran flavonoids – Found in tea. Secondary metabolite
		Kanzonol U	$[M+Na]^+$	308.1049	2-arylbenzofuran flavonoids
		Moracin G	$[M+Na]^+$	308.1049	2-arylbenzofuran flavonoids – Found in mulberries and fruits
		Moracin E	$[M+Na]^+$	308.1049	2-arylbenzofuran flavonoids – Found in mulberries and fruits
		Moracin D	$[M+Na]^+$	308.1049	2-arylbenzofuran flavonoids – Found in mulberries and fruits
		(1S,2R,3S)-2,3-Dihydro-4-(4-hydroxyphenyl)-1H-phenalene-1,2,3-triol	$[M+Na]^+$	308.1049	Phenylnaphthalenes – Found in fruits
		Warfarin	$[M+Na]^+$	308.1049	Anticoagulant that inhibits synthesis of vitamin-K-dependent coagulation factors. Used for prophylaxis, venous thrombosis, pulmonary embolism, atrial fibrillation with embolization. Also used as rodenticide and adjunct in prophylaxis of systemic embolism after myocardial infarction
359.2190	9.40	(3E,7E,11R,12E)-11-Hydroxy-3,7,11,15-tetramethyl-14-oxohexadeca-3,7,12-trienoic acid	$[M+Na]^+$	336.2301	Acyclic diterpenoids
		1H-Purine-2,6-dione, 7-(2-(ethylamino)ethyl)-3,7-dihydro-1,3-dimethyl-8-(phenylmethyl)-	$[M+NH_4]^+$	341.1852	Xanthines
		8S,15S-Dihydroxy-5Z,9E,11Z,13E-eicosatetraenoic acid	$[M+Na]^+$	336.2301	Hydroxyeicosatetraenoic acids
		12-Hydroperoxy-5,8,10,14-eicosatetraenoic acid	$[M+Na]^+$	336.2301	Hydroperoxyeicosatraenoic acids
		(+/-)-11,12-Dihydroxy-5Z,8Z,14Z,17Z-eicosatetraenoic acid	$[M+Na]^+$	336.2301	Hydroxyeicosatetraenoic acids

		5(S),15(R)-DiHETE(1-)	$[M+Na]^+$	336.2301	Hydroxyeicosatetraenoic acids
		5(S),15(R)-DiHETE	$[M+Na]^+$	336.2301	Eicosanoid lipid molecule
		8,20-DiHETE	$[M+Na]^+$	336.2301	Hydroxyeicosatetraenoic acids
		15-HPETE	$[M+Na]^+$	336.2301	Hydroperoxyeicosatraenoic acids
		(5Z,9E,14Z)-(8xi,11R,12S)-11,12-epoxy-8-hydroxyicosa-5,9,14-trienoic Acid	$[M+Na]^+$	336.2301	Hepoxilins
		5,20-DiHETE	$[M+Na]^+$	336.2301	Hydroxyeicosatetraenoic acids
		5(S),11(R)-DiHETE	$[M+Na]^+$	336.2301	Hydroxyeicosatetraenoic acids
		10-hydroxy-11S,12S-epoxy-5Z,8Z,14Z-eicosatrienoic acid	$[M+Na]^+$	336.2301	Hepoxilins
		5,12-dihydroxy-6,8,10,14-eicosatetraenoic acid	$[M+Na]^+$	336.2301	Leukotrienes
		Fenethylline	$[M+NH_4]^+$	341.1852	Xanthines
		15-Hydroperoxyicosa-5,8,11,13-tetraenoic acid	$[M+Na]^+$	336.2301	Hydroperoxyeicosatraenoic acids
337.1559	10.65	(2R,3S,9R)-5-Acetyl-8,8-dimethyl-7,16-diazapentacyclo[9.6.1.02,9.03,7.015,18]octadeca-1(17),11(18),12,14-tetraene-4,6-dione	$[M+H]^+$	336.1474	3-alkylindoles
		Valeroyl phenytoin	$[M+H]^+$	336.1474	Phenylhydantoins
		17-Ethynyl-16-fluoroestradiol	$[M+Na]^+$	314.1682	Estrogens and derivatives
		2-Propenamide, 2-cyano-3-(3,4-dihydroxyphenyl)-N-(4-phenylbutyl)-	$[M+H]^+$	336.1474	Hydroxycinnamic acids and derivatives
		Nb-Feruloyltryptamine	$[M+H]^+$	336.1474	Hydroxycinnamic acids and derivatives – Found in cereal, fats, and oils

Table S5: Complete List of Tentatively Identified Endogenous Metabolites in Moderate vs. High Models

m/z	RT	Tentative ID	Adduct	Monoisotopic Mass	Biological Significance
		2-Methyl-N-(4-Methylphenyl)alanine	$[M+NH_4]^+$	193.1103	Alpha amino acids and derivatives
		L,L-Cyclo(leucylprolyl)	$[M+H]^+$	210.1368	Alpha amino acids and derivatives – Secondary metabolite
		(R)-1,2-dimethyl-5,6-dihydroxy-tetrahydroisoquinoline	$[M+NH_4]^+$	193.1103	Derivative of Tetrahydroisoquinoline – Association with Parkinson's disease
211.1441	3.90	(R)-N-Methylsalsolinol	$[M+NH_4]^+$	193.1103	Most potent toxin among isoquinolines – Endogenous neurotoxins for dopamine neurons. Implicated in Parkinson's disease
		undec-3-enedioic acid	$[M+H]^+$	214.1205	Medium-chain fatty acids
		undec-4-enedioic acid	$[M+H]^+$	214.1205	Medium-chain fatty acids
		(2E)-undec-2-enedioic acid	$[M+H]^+$	214.1205	Medium-chain fatty acids
215.1279	4.78	undec-5-enedioic acid	$[M+H]^+$	214.1205	Medium-chain fatty acids
		dodec-6-enedioic acid	$[M+H]^+$	228.1362	Medium-chain fatty acid
		dodec-4-enedioic acid	$[M+H]^+$	228.1362	Medium-chain fatty acid
		dodec-5-enedioic acid	$[M+H]^+$	228.1362	Medium-chain fatty acid
		dodec-2-enedioic acid	$[M+H]^+$	228.1362	Medium-chain fatty acid
229.1433	5.30	dodec-3-enedioic acid	$[M+H]^+$	228.1362	Medium-chain fatty acid
		PG(20:4(8Z,11Z,14Z,17Z)-2OH(5S,6R)/i-12:0)	$[M+Na]^+$	746.4370	Oxidized phosphatidylglycerol
		PG(i-12:0/20:4(8Z,11Z,14Z,17Z)-2OH(5S,6R))	$[M+Na]^+$	746.4370	Oxidized phosphatidylglycerol
		PG(20:4(6E,8Z,11Z,13E)-2OH(5S,15S)/i-12:0)	$[M+Na]^+$	746.4370	Oxidized phosphatidylglycerol
		PG(i-12:0/20:4(6E,8Z,11Z,13E)-2OH(5S,15S))	$[M+Na]^+$	746.4370	Oxidized phosphatidylglycerol
		PG(20:4(6Z,8E,10E,14Z)-2OH(5S,12R)/i-12:0)	$[M+Na]^+$	746.4370	Oxidized phosphatidylglycerol
769.4224	21.08	PG(i-12:0/20:4(6Z,8E,10E,14Z)-2OH(5S,12R))	$[M+Na]^+$	746.4370	Oxidized phosphatidylglycerol
760.5845	20.22	1-Palmitoyl-2-oleoyl-sn-glycero-3-phosphocholine	$[M+H]^+$	759.5778	Phosphatidylcholines

		PE-NMe2(20:1(11Z)/15:0)	[M+H]+	759.5778	Dimethylphosphatidylethanolamine
		PE-NMe(22:1(13Z)/14:0)	[M+H]+	759.5778	Monomethylphosphatidylethanolamine
		PE-NMe(22:0/14:1(9Z))	[M+H]+	759.5778	Monomethylphosphatidylethanolamine
		PE-NMe(20:1(11Z)/16:0)	[M+H]+	759.5778	Monomethylphosphatidylethanolamine
		PE-NMe(20:0/16:1(9Z))	[M+H]+	759.5778	Monomethylphosphatidylethanolamine
		PE-NMe(18:1(9Z)/18:0)	[M+H]+	759.5778	Monomethylphosphatidylethanolamine
		PE-NMe(18:1(11Z)/18:0)	[M+H]+	759.5778	Monomethylphosphatidylethanolamine
		PE-NMe(14:0/22:1(13Z))	[M+H]+	759.5778	Monomethylphosphatidylethanolamine
		PE-NMe2(15:0/20:1(11Z))	[M+H]+	759.5778	Dimethylphosphatidylethanolamine
		PE-NMe(16:1(9Z)/20:0)	[M+H]+	759.5778	Monomethylphosphatidylethanolamine
		PE-NMe(14:1(9Z)/22:0)	[M+H]+	759.5778	Monomethylphosphatidylethanolamine
		PE-NMe(16:0/20:1(11Z))	[M+H]+	759.5778	Monomethylphosphatidylethanolamine
		PE-NMe(18:0/18:1(11Z))	[M+H]+	759.5778	Monomethylphosphatidylethanolamine
		PE-NMe(18:0/18:1(9Z))	[M+H]+	759.5778	Monomethylphosphatidylethanolamine
		PE(22:1(13Z)/15:0)	[M+H]+	759.5778	phosphatidylethanolamine
		PE(15:0/22:1(13Z))	[M+H]+	759.5778	Phosphatidylethanolamine
		PC(20:1(11Z)/14:0)	[M+H]+	759.5778	Phosphatidylcholine
		PC(20:0/14:1(9Z))	[M+H]+	759.5778	Phosphatidylcholine

		PC(18:1(9Z)/16:0)	[M+H]+	759.5778	Phosphatidylcholine
		PC(18:1(11Z)/16:0)	[M+H]+	759.5778	Phosphatidylcholine
		PC(18:0/16:1(9Z))	[M+H]+	759.5778	Phosphatidylcholine
		PC(16:1(9Z)/18:0)	[M+H]+	759.5778	Phosphatidylcholine
		PC(16:0/18:1(9Z))	[M+H]+	759.5778	Phosphatidylcholine
		PC(16:0/18:1(11Z))	[M+H]+	759.5778	Phosphatidylcholine
		PC(14:1(9Z)/20:0)	[M+H]+	759.5778	Phosphatidylcholine
		PC(14:0/20:1(11Z))	[M+H]+	759.5778	Phosphatidylcholine
		PC(20:5(6E,8Z,11Z,14Z,17Z)-OH(5)/P-16:0)	[M+Na]+	779.5465	Oxidized Phosphatidylcholine
		PC(P-16:0/20:5(6E,8Z,11Z,14Z,17Z)-OH(5))	[M+Na]+	779.5465	Oxidized Phosphatidylcholine
		PC(20:5(5Z,8Z,10E,14Z,17Z)-OH(12)/P-16:0)	[M+Na]+	779.5465	Oxidized Phosphatidylcholine
		PC(P-16:0/20:5(5Z,8Z,10E,14Z,17Z)-OH(12))	[M+Na]+	779.5465	Oxidized Phosphatidylcholine
		PC(20:5(5Z,8Z,11Z,14Z,16E)-OH(18)/P-16:0)	[M+Na]+	779.5465	Oxidized Phosphatidylcholine
		PC(P-16:0/20:5(5Z,8Z,11Z,14Z,16E)-OH(18))	[M+Na]+	779.5465	Oxidized Phosphatidylcholine
		PC(20:5(5Z,8Z,11Z,14Z,16E)-OH(18R)/P-16:0)	[M+Na]+	779.5465	Oxidized Phosphatidylcholine
		PC(P-16:0/20:5(5Z,8Z,11Z,14Z,16E)-OH(18R))	[M+Na]+	779.5465	Oxidized Phosphatidylcholine
		PC(20:4(5Z,8Z,11Z,13E)+=O(15)/P-16:0)	[M+Na]+	779.5465	Oxidized Phosphatidylcholine
		PC(P-16:0/20:4(5Z,8Z,11Z,13E)+=O(15))	[M+Na]+	779.5465	Oxidized Phosphatidylcholine
		PC(20:4(6E,8Z,11Z,14Z)+=O(5)/P-16:0)	[M+Na]+	779.5465	Oxidized Phosphatidylcholine
		PC(P-16:0/20:4(6E,8Z,11Z,14Z)+=O(5))	[M+Na]+	779.5465	Oxidized Phosphatidylcholine
		PE-NMe(20:2(11Z,14Z)/18:3(6Z,9Z,12Z))	[M+Na]+	779.5465	DimethylPhosphatidylethanolamine
		PE-NMe(20:2(11Z,14Z)/18:3(9Z,12Z,15Z))	[M+Na]+	779.5465	DimethylPhosphatidylethanolamine
		PE-NMe(18:3(9Z,12Z,15Z)/20:2(11Z,14Z))	[M+Na]+	779.5465	DimethylPhosphatidylethanolamine
		PE-NMe(18:3(6Z,9Z,12Z)/20:2(11Z,14Z))	[M+Na]+	779.5465	DimethylPhosphatidylethanolamine
		PE-NMe2(22:5(7Z,10Z,13Z,16Z,19Z)/15:0)	[M+Na]+	779.5465	DimethylPhosphatidylethanolamine
		PE-NMe2(22:5(4Z,7Z,10Z,13Z,16Z)/15:0)	[M+Na]+	779.5465	DimethylPhosphatidylethanolamine
		PE-NMe2(15:0/22:5(7Z,10Z,13Z,16Z,19Z))	[M+Na]+	779.5465	DimethylPhosphatidylethanolamine
		PE-NMe2(15:0/22:5(4Z,7Z,10Z,13Z,16Z))	[M+Na]+	779.5465	DimethylPhosphatidylethanolamine
802.5350	19.32	PE-NMe(22:5(7Z,10Z,13Z,16Z,19Z)/16:0)	[M+Na]+	779.5465	MonomethylPhosphatidylethanolamine

		PE-NMe(22:5(4Z,7Z,10Z,13Z,16Z)/16:0)	$[M+Na]^+$	779.5465	MonomethylPhosphatidylethanolamine
		PE-NMe(22:4(7Z,10Z,13Z,16Z)/16:1(9Z))	$[M+Na]^+$	779.5465	MonomethylPhosphatidylethanolamine
		PE-NMe(20:5(5Z,8Z,11Z,14Z,17Z)/18:0)	$[M+Na]^+$	779.5465	MonomethylPhosphatidylethanolamine
		PE-NMe(20:4(8Z,11Z,14Z,17Z)/18:1(9Z))	$[M+Na]^+$	779.5465	MonomethylPhosphatidylethanolamine
		PE-NMe(20:4(8Z,11Z,14Z,17Z)/18:1(11Z))	$[M+Na]^+$	779.5465	MonomethylPhosphatidylethanolamine
		PE-NMe(20:4(5Z,8Z,11Z,14Z)/18:1(9Z))	$[M+Na]^+$	779.5465	MonomethylPhosphatidylethanolamine
		PE-NMe(20:4(5Z,8Z,11Z,14Z)/18:1(11Z))	$[M+Na]^+$	779.5465	MonomethylPhosphatidylethanolamine
		PE-NMe(20:3(8Z,11Z,14Z)/18:2(9Z,12Z))	$[M+Na]^+$	779.5465	MonomethylPhosphatidylethanolamine
		PE-NMe(20:3(5Z,8Z,11Z)/18:2(9Z,12Z))	$[M+Na]^+$	779.5465	MonomethylPhosphatidylethanolamine
		PE-NMe(20:1(11Z)/18:4(6Z,9Z,12Z,15Z))	$[M+Na]^+$	779.5465	MonomethylPhosphatidylethanolamine
		PE-NMe(18:4(6Z,9Z,12Z,15Z)/20:1(11Z))	$[M+Na]^+$	779.5465	MonomethylPhosphatidylethanolamine
		PE-NMe(18:2(9Z,12Z)/20:3(8Z,11Z,14Z))	$[M+Na]^+$	779.5465	MonomethylPhosphatidylethanolamine
		PE-NMe(18:2(9Z,12Z)/20:3(5Z,8Z,11Z))	$[M+Na]^+$	779.5465	MonomethylPhosphatidylethanolamine
		PE-NMe(18:1(9Z)/20:4(8Z,11Z,14Z,17Z))	$[M+Na]^+$	779.5465	MonomethylPhosphatidylethanolamine
		PE-NMe(18:1(9Z)/20:4(5Z,8Z,11Z,14Z))	$[M+Na]^+$	779.5465	MonomethylPhosphatidylethanolamine

		PE-NMe(18:1(11Z)/20:4(5Z,8Z,11Z,14Z))	$[M+Na]^+$	779.5465	MonomethylPhosphatidylethanolamine
		PE-NMe(18:1(11Z)/20:4(8Z,11Z,14Z,17Z))	$[M+Na]^+$	779.5465	MonomethylPhosphatidylethanolamine
		PE-NMe(18:0/20:5(5Z,8Z,11Z,14Z,17Z))	$[M+Na]^+$	779.5465	MonomethylPhosphatidylethanolamine
		PE-NMe(16:1(9Z)/22:4(7Z,10Z,13Z,16Z))	$[M+Na]^+$	779.5465	MonomethylPhosphatidylethanolamine
		PE-NMe(16:0/22:5(7Z,10Z,13Z,16Z,19Z))	$[M+Na]^+$	779.5465	MonomethylPhosphatidylethanolamine
		PE-NMe(16:0/22:5(4Z,7Z,10Z,13Z,16Z))	$[M+Na]^+$	779.5465	MonomethylPhosphatidylethanolamine
		PE-NMe(22:6(4Z,7Z,10Z,13Z,16Z,19Z)/18:2(9Z,12Z))	$[M+H]^+$	801.5309	MonomethylPhosphatidylethanolamine
		PE-NMe(22:5(7Z,10Z,13Z,16Z,19Z)/18:3(6Z,9Z,12Z))	$[M+H]^+$	801.5309	MonomethylPhosphatidylethanolamine
		PE-NMe(22:5(7Z,10Z,13Z,16Z,19Z)/18:3(9Z,12Z,15Z))	$[M+H]^+$	801.5309	MonomethylPhosphatidylethanolamine
		PE-NMe(22:5(4Z,7Z,10Z,13Z,16Z)/18:3(9Z,12Z,15Z))	$[M+H]^+$	801.5309	MonomethylPhosphatidylethanolamine
		PE-NMe(22:5(4Z,7Z,10Z,13Z,16Z)/18:3(6Z,9Z,12Z))	$[M+H]^+$	801.5309	MonomethylPhosphatidylethanolamine
		PE-NMe(22:4(7Z,10Z,13Z,16Z)/18:4(6Z,9Z,12Z,15Z))	$[M+H]^+$	801.5309	MonomethylPhosphatidylethanolamine

		PE-NMe(20:5(5Z,8Z,11Z,14Z,17Z)/20:3(8Z,11Z,14Z))	$[M+H]^+$	801.5309	MonomethylPhosphatidylethanolamine
		PE-NMe(20:5(5Z,8Z,11Z,14Z,17Z)/20:3(5Z,8Z,11Z))	$[M+H]^+$	801.5309	MonomethylPhosphatidylethanolamine
		PE-NMe(20:4(8Z,11Z,14Z,17Z)/20:4(5Z,8Z,11Z,14Z))	$[M+H]^+$	801.5309	MonomethylPhosphatidylethanolamine
		PE-NMe(20:4(8Z,11Z,14Z,17Z)/20:4(8Z,11Z,14Z,17Z))	$[M+H]^+$	801.5309	MonomethylPhosphatidylethanolamine
		PE-NMe(20:4(5Z,8Z,11Z,14Z)/20:4(8Z,11Z,14Z,17Z))	$[M+H]^+$	801.5309	MonomethylPhosphatidylethanolamine
		PE-NMe(20:3(8Z,11Z,14Z)/20:5(5Z,8Z,11Z,14Z,17Z))	$[M+H]^+$	801.5309	MonomethylPhosphatidylethanolamine
		PE-NMe(20:3(5Z,8Z,11Z)/20:5(5Z,8Z,11Z,14Z,17Z))	$[M+H]^+$	801.5309	MonomethylPhosphatidylethanolamine
		PE-NMe(18:4(6Z,9Z,12Z,15Z)/22:4(7Z,10Z,13Z,16Z))	$[M+H]^+$	801.5309	MonomethylPhosphatidylethanolamine
		PE-NMe(18:3(9Z,12Z,15Z)/22:5(7Z,10Z,13Z,16Z,19Z))	$[M+H]^+$	801.5309	MonomethylPhosphatidylethanolamine
		PE-NMe(18:3(9Z,12Z,15Z)/22:5(4Z,7Z,10Z,13Z,16Z))	$[M+H]^+$	801.5309	MonomethylPhosphatidylethanolamine

		PE-NMe(18:3(6Z,9Z,12Z)/22:5(4Z,7Z,10Z,13Z,16Z))	[M+H]+	801.5309	MonomethylPhosphatidylethanolamine
		PE-NMe(18:3(6Z,9Z,12Z)/22:5(7Z,10Z,13Z,16Z,19Z))	[M+H]+	801.5309	MonomethylPhosphatidylethanolamine
		PE-NMe(18:2(9Z,12Z)/22:6(4Z,7Z,10Z,13Z,16Z,19Z))	[M+H]+	801.5309	MonomethylPhosphatidylethanolamine
		PE-NMe(20:4(5Z,8Z,11Z,14Z)/20:4(5Z,8Z,11Z,14Z))	[M+H]+	801.5309	MonomethylPhosphatidylethanolamine
		PC(22:5(7Z,10Z,13Z,16Z,19Z)/14:0)	[M+Na]+	779.5465	Phosphatidylcholine
		PC(22:5(4Z,7Z,10Z,13Z,16Z)/14:0)	[M+Na]+	779.5465	Phosphatidylcholine
		PC(22:4(7Z,10Z,13Z,16Z)/14:1(9Z))	[M+Na]+	779.5465	Phosphatidylcholine
		PC(20:5(5Z,8Z,11Z,14Z,17Z)/18:3(9Z,12Z,15Z))	[M+H]+	801.5309	Phosphatidylcholine
		PC(20:5(5Z,8Z,11Z,14Z,17Z)/18:3(6Z,9Z,12Z))	[M+H]+	801.5309	Phosphatidylcholine
		PC(20:5(5Z,8Z,11Z,14Z,17Z)/16:0)	[M+Na]+	779.5465	Phosphatidylcholine
		PC(20:4(8Z,11Z,14Z,17Z)/18:4(6Z,9Z,12Z,15Z))	[M+H]+	801.5309	Phosphatidylcholine
		PC(20:4(8Z,11Z,14Z,17Z)/16:1(9Z))	[M+Na]+	779.5465	Phosphatidylcholine
		PC(20:4(5Z,8Z,11Z,14Z)/18:4(6Z,9Z,12Z,15Z))	[M+H]+	801.5309	Phosphatidylcholine
		PC(20:4(5Z,8Z,11Z,14Z)/16:1(9Z))	[M+Na]+	779.5465	Phosphatidylcholine
		PC(18:4(6Z,9Z,12Z,15Z)/20:4(8Z,11Z,14Z,17Z))	[M+H]+	801.5309	Phosphatidylcholine
		PC(18:4(6Z,9Z,12Z,15Z)/20:4(5Z,8Z,11Z,14Z))	[M+H]+	801.5309	Phosphatidylcholine
		PC(18:4(6Z,9Z,12Z,15Z)/18:1(11Z))	[M+Na]+	779.5465	Phosphatidylcholine
		PC(18:4(6Z,9Z,12Z,15Z)/18:1(9Z))	[M+Na]+	779.5465	Phosphatidylcholine
		PC(18:3(9Z,12Z,15Z)/20:5(5Z,8Z,11Z,14Z,17Z))	[M+H]+	801.5309	Phosphatidylcholine
		PC(18:3(9Z,12Z,15Z)/18:2(9Z,12Z))	[M+Na]+	779.5465	Phosphatidylcholine
		PC(18:3(6Z,9Z,12Z)/20:5(5Z,8Z,11Z,14Z,17Z))	[M+H]+	801.5309	Phosphatidylcholine
		PC(18:3(6Z,9Z,12Z)/18:2(9Z,12Z))	[M+Na]+	779.5465	Phosphatidylcholine

		PC(18:2(9Z,12Z)/18:3(9Z,12Z,15Z))	$[M+Na]^+$	779.5465	Phosphatidylcholine
		PC(18:2(9Z,12Z)/18:3(6Z,9Z,12Z))	$[M+Na]^+$	779.5465	Phosphatidylcholine
		PC(18:1(9Z)/18:4(6Z,9Z,12Z,15Z))	$[M+Na]^+$	779.5465	Phosphatidylcholine
		PC(18:1(11Z)/18:4(6Z,9Z,12Z,15Z))	$[M+Na]^+$	779.5465	Phosphatidylcholine
		PC(16:1(9Z)/20:4(8Z,11Z,14Z,17Z))	$[M+Na]^+$	779.5465	Phosphatidylcholine
		PC(16:1(9Z)/20:4(5Z,8Z,11Z,14Z))	$[M+Na]^+$	779.5465	Phosphatidylcholine
		PC(16:0/20:5(5Z,8Z,11Z,14Z,17Z))	$[M+Na]^+$	779.5465	Phosphatidylcholine
		PC(14:1(9Z)/22:4(7Z,10Z,13Z,16Z))	$[M+Na]^+$	779.5465	Phosphatidylcholine
		PC(14:0/22:5(7Z,10Z,13Z,16Z,19Z))	$[M+Na]^+$	779.5465	Phosphatidylcholine
		PC(14:0/22:5(4Z,7Z,10Z,13Z,16Z))	$[M+Na]^+$	779.5465	Phosphatidylcholine
		LysoPC(0:0/16:0)	$[M+H]^+$	495.3325	LysoPhosphatidylcholine
		1-PalmitoylPhosphatidylcholine	$[M+H]^+$	495.3325	PalmitoylPhosphatidylcholine
		23-Acetoxysoladulcidine	$[M+Na]^+$	473.3505	Steroidal alkaloid
		LysoPC(16:0/0:0)	$[M+H]^+$	495.3325	LysoPhosphatidylcholine
		Clupanodonyl carnitine	$[M+Na]^+$	473.3505	Acylcarnitine
496.3397	12.95	Docosa-4,7,10,13,16-pentaenoyl carnitine	$[M+Na]^+$	473.3505	Acylcarnitine
		LysoPC(0:0/20:4(5Z,8Z,11Z,14Z))	$[M+H]^+$	543.3325	LysoPhosphatidylcholine
		LysoPC(0:0/18:1(9Z))	$[M+Na]^+$	521.3481	LysoPhosphatidylcholine
		LysoPC(20:4(8Z,11Z,14Z,17Z)/0:0)	$[M+H]^+$	543.3325	LysoPhosphatidylcholine
		LysoPC(20:4(5Z,8Z,11Z,14Z)/0:0)	$[M+H]^+$	543.3325	LysoPhosphatidylcholine
		LysoPC(18:1(11Z)/0:0)	$[M+Na]^+$	521.3481	LysoPhosphatidylcholine
544.3373	13.35	LysoPC(18:1(9Z)/0:0)	$[M+Na]^+$	521.3481	LysoPhosphatidylcholine
		LysoPC(0:0/16:0)	$[M+Na]^+$	495.3325	LysoPhosphatidylcholine
		1-PalmitoylPhosphatidylcholine	$[M+Na]^+$	495.3325	PalmitoylPhosphatidylcholine
518.3215	12.95	LysoPC(16:0/0:0)	$[M+Na]^+$	495.3325	LysoPhosphatidylcholine
		MG(16:1(9Z)/0:0/0:0)	$[M+Na]^+$	328.2614	Monoacylglyceride
351.2504	12.67	MG(0:0/16:1(9Z)/0:0)	$[M+Na]^+$	328.2614	Monoacylglyceride
384.3833*	13.74	Nervonic acid	$[M+NH_4]^+$	366.3498	Long chain unsaturated fatty acid that is enriched in sphingomyelin
828.5507	19.67	PC(20:5(6E,8Z,11Z,14Z,17Z)-OH(5)/P-18:1(9Z))	$[M+Na]^+$	805.5622	Oxidized Phosphatidylcholine

		PC(P-18:1(9Z)/20:5(6E,8Z,11Z,14Z,17Z)-OH(5))	[M+Na]+	805.5622	Oxidized Phosphatidylcholine
		PC(20:5(5Z,8Z,10E,14Z,17Z)-OH(12)/P-18:1(9Z))	[M+Na]+	805.5622	Oxidized Phosphatidylcholine
		PC(P-18:1(9Z)/20:5(5Z,8Z,10E,14Z,17Z)-OH(12))	[M+Na]+	805.5622	Oxidized Phosphatidylcholine
		PC(20:5(5Z,8Z,11Z,14Z,16E)-OH(18)/P-18:1(9Z))	[M+Na]+	805.5622	Oxidized Phosphatidylcholine
		PC(P-18:1(9Z)/20:5(5Z,8Z,11Z,14Z,16E)-OH(18))	[M+Na]+	805.5622	Oxidized Phosphatidylcholine
		PC(20:5(5Z,8Z,11Z,14Z,16E)-OH(18R)/P-18:1(9Z))	[M+Na]+	805.5622	Oxidized Phosphatidylcholine
		PC(P-18:1(9Z)/20:5(5Z,8Z,11Z,14Z,16E)-OH(18R))	[M+Na]+	805.5622	Oxidized Phosphatidylcholine
		PC(20:4(5Z,8Z,11Z,13E)+=O(15)/P-18:1(9Z))	[M+Na]+	805.5622	Oxidized Phosphatidylcholine
		PC(P-18:1(9Z)/20:4(5Z,8Z,11Z,13E)+=O(15))	[M+Na]+	805.5622	Oxidized Phosphatidylcholine
		PC(20:4(6E,8Z,11Z,14Z)+=O(5)/P-18:1(9Z))	[M+Na]+	805.5622	Oxidized Phosphatidylcholine
		PC(P-18:1(9Z)/20:4(6E,8Z,11Z,14Z)+=O(5))	[M+Na]+	805.5622	Oxidized Phosphatidylcholine
		PC(20:5(6E,8Z,11Z,14Z,17Z)-OH(5)/P-18:1(11Z))	[M+Na]+	805.5622	Oxidized Phosphatidylcholine
		PC(P-18:1(11Z)/20:5(6E,8Z,11Z,14Z,17Z)-OH(5))	[M+Na]+	805.5622	Oxidized Phosphatidylcholine
		PC(20:5(5Z,8Z,10E,14Z,17Z)-OH(12)/P-18:1(11Z))	[M+Na]+	805.5622	Oxidized Phosphatidylcholine
		PC(P-18:1(11Z)/20:5(5Z,8Z,10E,14Z,17Z)-OH(12))	[M+Na]+	805.5622	Oxidized Phosphatidylcholine
		PC(20:5(5Z,8Z,11Z,14Z,16E)-OH(18)/P-18:1(11Z))	[M+Na]+	805.5622	Oxidized Phosphatidylcholine
		PC(P-18:1(11Z)/20:5(5Z,8Z,11Z,14Z,16E)-OH(18))	[M+Na]+	805.5622	Oxidized Phosphatidylcholine

		PC(20:5(5Z,8Z,11Z,14Z,16E)-OH(18R)/P-18:1(11Z))	[M+Na]+	805.5622	Oxidized Phosphatidylcholine
		PC(P-18:1(11Z)/20:5(5Z,8Z,11Z,14Z,16E)-OH(18R))	[M+Na]+	805.5622	Oxidized Phosphatidylcholine
		PC(20:4(5Z,8Z,11Z,13E)+=O(15)/P-18:1(11Z))	[M+Na]+	805.5622	Oxidized Phosphatidylcholine
		PC(P-18:1(11Z)/20:4(5Z,8Z,11Z,13E)+=O(15))	[M+Na]+	805.5622	Oxidized Phosphatidylcholine
		PC(20:4(6E,8Z,11Z,14Z)+=O(5)/P-18:1(11Z))	[M+Na]+	805.5622	Oxidized Phosphatidylcholine
		PC(P-18:1(11Z)/20:4(6E,8Z,11Z,14Z)+=O(5))	[M+Na]+	805.5622	Oxidized Phosphatidylcholine
		PC(22:5(4Z,7Z,10Z,13Z,19Z)-O(16,17)/P-16:0)	[M+Na]+	805.5622	Oxidized Phosphatidylcholine
		PC(P-16:0/22:5(4Z,7Z,10Z,13Z,19Z)-O(16,17))	[M+Na]+	805.5622	Oxidized Phosphatidylcholine
		PC(22:6(4Z,7Z,10Z,13E,15E,19Z)-OH(17)/P-16:0)	[M+Na]+	805.5622	Oxidized Phosphatidylcholine
		PC(P-16:0/22:6(4Z,7Z,10Z,13E,15E,19Z)-OH(17))	[M+Na]+	805.5622	Oxidized Phosphatidylcholine
		PC(22:6(4Z,7Z,10Z,12E,16Z,19Z)-OH(14)/P-16:0)	[M+Na]+	805.5622	Oxidized Phosphatidylcholine
		PC(P-16:0/22:6(4Z,7Z,10Z,12E,16Z,19Z)-OH(14))	[M+Na]+	805.5622	Oxidized Phosphatidylcholine
		PC(22:6(4Z,8Z,10Z,13Z,16Z,19Z)-OH(7)/P-16:0)	[M+Na]+	805.5622	Oxidized Phosphatidylcholine
		PC(P-16:0/22:6(4Z,8Z,10Z,13Z,16Z,19Z)-OH(7))	[M+Na]+	805.5622	Oxidized Phosphatidylcholine
		PC(22:6(5Z,7Z,10Z,13Z,16Z,19Z)-OH(4)/P-16:0)	[M+Na]+	805.5622	Oxidized Phosphatidylcholine
		PC(P-16:0/22:6(5Z,7Z,10Z,13Z,16Z,19Z)-OH(4))	[M+Na]+	805.5622	Oxidized Phosphatidylcholine
		PE-NMe(20:4(8Z,11Z,14Z,17Z)/20:2(11Z,14Z))	[M+Na]+	805.5622	MonomethylPhosphatidylethanolamine
		PE-NMe(20:4(5Z,8Z,11Z,14Z)/20:2(11Z,14Z))	[M+Na]+	805.5622	MonomethylPhosphatidylethanolamine
		PE-NMe(20:2(11Z,14Z)/20:4(8Z,11Z,14Z,17Z))	[M+Na]+	805.5622	MonomethylPhosphatidylethanolamine
		PE-NMe(20:2(11Z,14Z)/20:4(5Z,8Z,11Z,14Z))	[M+Na]+	805.5622	MonomethylPhosphatidylethanolamine

		PE-NMe(22:6(4Z,7Z,10Z,13Z,16Z,19Z)/18:0)	$[M+Na]^+$	805.5622	MonomethylPhosphatidylethanolam ine
		PE-NMe(22:5(7Z,10Z,13Z,16Z,19Z)/18:1(11Z))	$[M+Na]^+$	805.5622	MonomethylPhosphatidylethanolam ine
		PE-NMe(22:5(7Z,10Z,13Z,16Z,19Z)/18:1(9Z))	$[M+Na]^+$	805.5622	MonomethylPhosphatidylethanolam ine
		PE-NMe(22:5(4Z,7Z,10Z,13Z,16Z)/18:1(9Z))	$[M+Na]^+$	805.5622	MonomethylPhosphatidylethanolam ine
		PE-NMe(22:5(4Z,7Z,10Z,13Z,16Z)/18:1(11Z))	$[M+Na]^+$	805.5622	MonomethylPhosphatidylethanolam ine
		PE-NMe(22:4(7Z,10Z,13Z,16Z)/18:2(9Z,12Z))	$[M+Na]^+$	805.5622	MonomethylPhosphatidylethanolam ine
		PE-NMe(22:2(13Z,16Z)/18:4(6Z,9Z,12Z,15Z))	$[M+Na]^+$	805.5622	MonomethylPhosphatidylethanolam ine
		PE-NMe(20:5(5Z,8Z,11Z,14Z,17Z)/20:1(11Z))	$[M+Na]^+$	805.5622	MonomethylPhosphatidylethanolam ine
		PE-NMe(20:3(8Z,11Z,14Z)/20:3(8Z,11Z,14Z))	$[M+Na]^+$	805.5622	MonomethylPhosphatidylethanolam ine
		PE-NMe(20:3(8Z,11Z,14Z)/20:3(5Z,8Z,11Z))	$[M+Na]^+$	805.5622	MonomethylPhosphatidylethanolam ine
		PE-NMe(20:3(5Z,8Z,11Z)/20:3(8Z,11Z,14Z))	$[M+Na]^+$	805.5622	MonomethylPhosphatidylethanolam ine
		PE-NMe(20:3(5Z,8Z,11Z)/20:3(5Z,8Z,11Z))	$[M+Na]^+$	805.5622	MonomethylPhosphatidylethanolam ine
		PE-NMe(20:1(11Z)/20:5(5Z,8Z,11Z,14Z,17Z))	$[M+Na]^+$	805.5622	MonomethylPhosphatidylethanolam ine
		PE-NMe(18:4(6Z,9Z,12Z,15Z)/22:2(13Z,16Z))	$[M+Na]^+$	805.5622	MonomethylPhosphatidylethanolam ine
		PE-NMe(18:2(9Z,12Z)/22:4(7Z,10Z,13Z,16Z))	$[M+Na]^+$	805.5622	MonomethylPhosphatidylethanolam ine

		PE-NMe(18:1(9Z)/22:5(7Z,10Z,13Z,16Z,19Z))	[M+Na]+	805.5622	MonomethylPhosphatidylethanolamine
		PE-NMe(18:1(9Z)/22:5(4Z,7Z,10Z,13Z,16Z))	[M+Na]+	805.5622	MonomethylPhosphatidylethanolamine
		PE-NMe(18:1(11Z)/22:5(4Z,7Z,10Z,13Z,16Z))	[M+Na]+	805.5622	MonomethylPhosphatidylethanolamine
		PE-NMe(18:1(11Z)/22:5(7Z,10Z,13Z,16Z,19Z))	[M+Na]+	805.5622	MonomethylPhosphatidylethanolamine
		PE-NMe(18:0/22:6(4Z,7Z,10Z,13Z,16Z,19Z))	[M+Na]+	805.5622	MonomethylPhosphatidylethanolamine
		PE-NMe(22:6(4Z,7Z,10Z,13Z,16Z,19Z)/20:3(5Z,8Z,11Z))	[M+H]+	827.5465	MonomethylPhosphatidylethanolamine
		PE-NMe(22:6(4Z,7Z,10Z,13Z,16Z,19Z)/20:3(8Z,11Z,14Z))	[M+H]+	827.5465	MonomethylPhosphatidylethanolamine
		PE-NMe(22:5(7Z,10Z,13Z,16Z,19Z)/20:4(8Z,11Z,14Z,17Z))	[M+H]+	827.5465	MonomethylPhosphatidylethanolamine
		PE-NMe(22:5(7Z,10Z,13Z,16Z,19Z)/20:4(5Z,8Z,11Z,14Z))	[M+H]+	827.5465	MonomethylPhosphatidylethanolamine
		PE-NMe(22:5(4Z,7Z,10Z,13Z,16Z)/20:4(8Z,11Z,14Z,17Z))	[M+H]+	827.5465	MonomethylPhosphatidylethanolamine
		PE-NMe(22:5(4Z,7Z,10Z,13Z,16Z)/20:4(5Z,8Z,11Z,14Z))	[M+H]+	827.5465	MonomethylPhosphatidylethanolamine
		PE-NMe(22:4(7Z,10Z,13Z,16Z)/20:5(5Z,8Z,11Z,14Z,17Z))	[M+H]+	827.5465	MonomethylPhosphatidylethanolamine

		PE-NMe(20:5(5Z,8Z,11Z,14Z,17Z)/22:4(7Z,10Z,13Z,16Z))	[M+H]+	827.5465	MonomethylPhosphatidylethanolamine
		PE-NMe(20:4(8Z,11Z,14Z,17Z)/22:5(7Z,10Z,13Z,16Z,19Z))	[M+H]+	827.5465	MonomethylPhosphatidylethanolamine
		PE-NMe(20:4(8Z,11Z,14Z,17Z)/22:5(4Z,7Z,10Z,13Z,16Z))	[M+H]+	827.5465	MonomethylPhosphatidylethanolamine
		PE-NMe(20:4(5Z,8Z,11Z,14Z)/22:5(4Z,7Z,10Z,13Z,16Z))	[M+H]+	827.5465	MonomethylPhosphatidylethanolamine
		PE-NMe(20:4(5Z,8Z,11Z,14Z)/22:5(7Z,10Z,13Z,16Z,19Z))	[M+H]+	827.5465	MonomethylPhosphatidylethanolamine
		PE-NMe(20:3(8Z,11Z,14Z)/22:6(4Z,7Z,10Z,13Z,16Z,19Z))	[M+H]+	827.5465	MonomethylPhosphatidylethanolamine
		PE-NMe(20:3(5Z,8Z,11Z)/22:6(4Z,7Z,10Z,13Z,16Z,19Z))	[M+H]+	827.5465	MonomethylPhosphatidylethanolamine
		PC(22:6(4Z,7Z,10Z,13Z,16Z,19Z)/18:3(9Z,12Z,15Z))	[M+H]+	827.5465	Phosphatidylcholine
		PC(22:6(4Z,7Z,10Z,13Z,16Z,19Z)/18:3(6Z,9Z,12Z))	[M+H]+	827.5465	Phosphatidylcholine
		PC(22:6(4Z,7Z,10Z,13Z,16Z,19Z)/16:0)	[M+Na]+	805.5622	Phosphatidylcholine
		PC(22:5(7Z,10Z,13Z,16Z,19Z)/18:4(6Z,9Z,12Z,15Z))	[M+H]+	827.5465	Phosphatidylcholine
		PC(22:5(7Z,10Z,13Z,16Z,19Z)/16:1(9Z))	[M+Na]+	805.5622	Phosphatidylcholine
		PC(22:5(4Z,7Z,10Z,13Z,16Z)/18:4(6Z,9Z,12Z,15Z))	[M+H]+	827.5465	Phosphatidylcholine

		PC(22:5(4Z,7Z,10Z,13Z,16Z)/16:1(9Z))	[M+Na]+	805.5622	Phosphatidylcholine
		PC(20:5(5Z,8Z,11Z,14Z,17Z)/20:4(5Z,8Z,11Z,14Z))	[M+H]+	827.5465	Phosphatidylcholine
		PC(20:5(5Z,8Z,11Z,14Z,17Z)/20:4(8Z,11Z,14Z,17Z))	[M+H]+	827.5465	Phosphatidylcholine
		PC(20:5(5Z,8Z,11Z,14Z,17Z)/18:1(11Z))	[M+Na]+	805.5622	Phosphatidylcholine
		PC(20:5(5Z,8Z,11Z,14Z,17Z)/18:1(9Z))	[M+Na]+	805.5622	Phosphatidylcholine
		PC(20:4(8Z,11Z,14Z,17Z)/20:5(5Z,8Z,11Z,14Z,17Z))	[M+H]+	827.5465	Phosphatidylcholine
		PC(20:4(8Z,11Z,14Z,17Z)/18:2(9Z,12Z))	[M+Na]+	805.5622	Phosphatidylcholine
		PC(20:4(5Z,8Z,11Z,14Z)/20:5(5Z,8Z,11Z,14Z,17Z))	[M+H]+	827.5465	Phosphatidylcholine
		PC(20:4(5Z,8Z,11Z,14Z)/18:2(9Z,12Z))	[M+Na]+	805.5622	Phosphatidylcholine
		PC(20:3(8Z,11Z,14Z)/18:3(9Z,12Z,15Z))	[M+Na]+	805.5622	Phosphatidylcholine
		PC(20:3(8Z,11Z,14Z)/18:3(6Z,9Z,12Z))	[M+Na]+	805.5622	Phosphatidylcholine
		PC(20:3(5Z,8Z,11Z)/18:3(6Z,9Z,12Z))	[M+Na]+	805.5622	Phosphatidylcholine
		PC(20:3(5Z,8Z,11Z)/18:3(9Z,12Z,15Z))	[M+Na]+	805.5622	Phosphatidylcholine
		PC(20:2(11Z,14Z)/18:4(6Z,9Z,12Z,15Z))	[M+Na]+	805.5622	Phosphatidylcholine
		PC(18:4(6Z,9Z,12Z,15Z)/22:5(4Z,7Z,10Z,13Z,16Z))	[M+H]+	827.5465	Phosphatidylcholine
		PC(18:4(6Z,9Z,12Z,15Z)/22:5(7Z,10Z,13Z,16Z,19Z))	[M+H]+	827.5465	Phosphatidylcholine
		PC(18:4(6Z,9Z,12Z,15Z)/20:2(11Z,14Z))	[M+Na]+	805.5622	Phosphatidylcholine
		PC(18:3(9Z,12Z,15Z)/22:6(4Z,7Z,10Z,13Z,16Z,19Z))	[M+H]+	827.5465	Phosphatidylcholine
		PC(18:3(9Z,12Z,15Z)/20:3(5Z,8Z,11Z))	[M+Na]+	805.5622	Phosphatidylcholine
		PC(18:3(9Z,12Z,15Z)/20:3(8Z,11Z,14Z))	[M+Na]+	805.5622	Phosphatidylcholine
		PC(18:3(6Z,9Z,12Z)/22:6(4Z,7Z,10Z,13Z,16Z,19Z))	[M+H]+	827.5465	Phosphatidylcholine
		PC(18:3(6Z,9Z,12Z)/20:3(5Z,8Z,11Z))	[M+Na]+	805.5622	Phosphatidylcholine

		PC(18:3(6Z,9Z,12Z)/20:3(8Z,11Z,14Z))	$[M+Na]^+$	805.5622	Phosphatidylcholine
		PC(18:2(9Z,12Z)/20:4(5Z,8Z,11Z,14Z))	$[M+Na]^+$	805.5622	Phosphatidylcholine
		PC(18:2(9Z,12Z)/20:4(8Z,11Z,14Z,17Z))	$[M+Na]^+$	805.5622	Phosphatidylcholine
		PC(18:1(9Z)/20:5(5Z,8Z,11Z,14Z,17Z))	$[M+Na]^+$	805.5622	Phosphatidylcholine
		PC(18:1(11Z)/20:5(5Z,8Z,11Z,14Z,17Z))	$[M+Na]^+$	805.5622	Phosphatidylcholine
		PC(16:1(9Z)/22:5(7Z,10Z,13Z,16Z,19Z))	$[M+Na]^+$	805.5622	Phosphatidylcholine
		PC(16:1(9Z)/22:5(4Z,7Z,10Z,13Z,16Z))	$[M+Na]^+$	805.5622	Phosphatidylcholine
		PC(16:0/22:6(4Z,7Z,10Z,13Z,16Z,19Z))	$[M+Na]^+$	805.5622	Phosphatidylcholine
		Valylcysteine	$[M+H]^+$	220.0882	Dipeptide of valine and cysteine – Incomplete breakdown of protein digestion or protein catabolism
		N-Acetyl-S-allylcysteine	$[M+NH_4]^+$	203.0616	N-acyl-l-alpha-amino acids
		1-[2-Methyl-3-(methylthio) allyl]cyclohex-2-enol	$[M+Na]^+$	198.1078	Tertiary alcohols – Can be found in feces
		Alanylmethionine	$[M+H]^+$	220.0882	Peptides – Found in anatidaes, chicken, pigs. Secondary metabolite
		Methionyl-Alanine	$[M+H]^+$	220.0882	Dipeptide of methionine and alanine – Incomplete breakdown of protein digestion or protein catabolism
221.0960	5.93	Cysteinyl-Valine	$[M+H]^+$	220.0882	Dipeptide of cysteinyl and valine – Incomplete breakdown product of protein digestion or protein catabolism
		PC(22:6(4Z,7Z,11E,13Z,15E,19Z)-2OH(10S,17)/17:0)	$[M+Na]^+$	851.5676	Oxidized phosphotidylcholine
		PC(17:0/22:6(4Z,7Z,11E,13Z,15E,19Z)-2OH(10S,17))	$[M+Na]^+$	851.5676	Oxidized phosphotidylcholine
874.5538	19.89	PC(22:6(5Z,8E,10Z,13Z,15E,19Z)-2OH(7S, 17S)/17:0)	$[M+Na]^+$	851.5676	Oxidized phosphotidylcholine

		PC(17:0/22:6(5Z,8E,10Z,13Z,15E,19Z)-2OH(7S, 17S))	[M+Na]+	851.5676	Oxidized phosphotidylcholine
		PE(18:3(9,11,15)-OH(13)/DiMe(13,5))	[M+Na]+	851.5676	Oxidized Phosphatidylethanolamine
		PE(DiMe(13,5)/18:3(9,11,15)-OH(13))	[M+Na]+	851.5676	Oxidized Phosphatidylethanolamine
		PE(18:3(10,12,15)-OH(9)/DiMe(13,5))	[M+Na]+	851.5676	Oxidized Phosphatidylethanolamine
		PE(DiMe(13,5)/18:3(10,12,15)-OH(9))	[M+Na]+	851.5676	Oxidized Phosphatidylethanolamine
		PE(18:2(9Z,11E)+=O(13)/DiMe(13,5))	[M+Na]+	851.5676	Oxidized Phosphatidylethanolamine
		PE(DiMe(13,5)/18:2(9Z,11E)+=O(13))	[M+Na]+	851.5676	Oxidized Phosphatidylethanolamine
		PE(18:2(10E,12Z)+=O(9)/DiMe(13,5))	[M+Na]+	851.5676	Oxidized Phosphatidylethanolamine
		PE(DiMe(13,5)/18:2(10E,12Z)+=O(9))	[M+Na]+	851.5676	Oxidized Phosphatidylethanolamine
		PE(20:3(6,8,11)-OH(5)/DiMe(11,5))	[M+Na]+	851.5676	Oxidized Phosphatidylethanolamine
		PE(DiMe(11,5)/20:3(6,8,11)-OH(5))	[M+Na]+	851.5676	Oxidized Phosphatidylethanolamine
		PE(20:4(8Z,11Z,14Z,17Z)-2OH(5S,6R)/22:2(13Z,16Z))	[M+Na]+	851.5676	Oxidized Phosphatidylethanolamine
		PE(22:2(13Z,16Z)/20:4(8Z,11Z,14Z,17Z)-2OH(5S,6R))	[M+Na]+	851.5676	Oxidized Phosphatidylethanolamine
		PE(20:4(6E,8Z,11Z,13E)-2OH(5S,15S)/22:2(13Z,16Z))	[M+Na]+	851.5676	Oxidized Phosphatidylethanolamine
		PE(22:2(13Z,16Z)/20:4(6E,8Z,11Z,13E)-2OH(5S,15S))	[M+Na]+	851.5676	Oxidized Phosphatidylethanolamine
		PE(20:4(6Z,8E,10E,14Z)-2OH(5S,12R)/22:2(13Z,16Z))	[M+Na]+	851.5676	Oxidized Phosphatidylethanolamine
		PE(22:2(13Z,16Z)/20:4(6Z,8E,10E,14Z)-2OH(5S,12R))	[M+Na]+	851.5676	Oxidized Phosphatidylethanolamine
		PE(PGJ2/22:1(13Z))	[M+Na]+	851.5676	Oxidized Phosphatidylethanolamine
		PE(22:1(13Z)/PGJ2)	[M+Na]+	851.5676	Oxidized Phosphatidylethanolamine
		PE(22:6(4Z,7Z,11E,13Z,15E,19Z)-2OH(10S,17)/20:0)	[M+Na]+	851.5676	Oxidized Phosphatidylethanolamine
		PE(20:0/22:6(4Z,7Z,11E,13Z,15E,19Z)-2OH(10S,17))	[M+Na]+	851.5676	Oxidized Phosphatidylethanolamine

		PE(22:6(5Z,8E,10Z,13Z,15E,19Z)-2OH(7S, 17S)/20:0)	$[M+Na]^+$	851.5676	Oxidized Phosphatidylethanolamine
		PE(20:0/22:6(5Z,8E,10Z,13Z,15E,19Z)-2OH(7S, 17S))	$[M+Na]^+$	851.5676	Oxidized Phosphatidylethanolamine
		PE-NMe2(9M5/11M5)	$[M+Na]^+$	851.5676	DimethylPhosphatidylethanolamine
		PE-NMe2(9D5/9D5)	$[M+Na]^+$	851.5676	DimethylPhosphatidylethanolamine
		PE-NMe2(9D5/11D3)	$[M+Na]^+$	851.5676	DimethylPhosphatidylethanolamine
		PE-NMe2(9D3/11D5)	$[M+Na]^+$	851.5676	DimethylPhosphatidylethanolamine
		PE-NMe2(11M5/9M5)	$[M+Na]^+$	851.5676	DimethylPhosphatidylethanolamine
		PE-NMe2(11M5/11M3)	$[M+Na]^+$	851.5676	DimethylPhosphatidylethanolamine
		PE-NMe2(11M3/11M5)	$[M+Na]^+$	851.5676	DimethylPhosphatidylethanolamine
		PE-NMe2(11D5/9D3)	$[M+Na]^+$	851.5676	DimethylPhosphatidylethanolamine
		PE-NMe2(11D3/9D5)	$[M+Na]^+$	851.5676	DimethylPhosphatidylethanolamine
		PE-NMe2(11D3/11D3)	$[M+Na]^+$	851.5676	DimethylPhosphatidylethanolamine
		PE-NMe(9M5/11D5)	$[M+Na]^+$	851.5676	MonomethylPhosphatidylethanolamine
		PE-NMe(9D5/11M5)	$[M+Na]^+$	851.5676	MonomethylPhosphatidylethanolamine
		PE-NMe(9D3/13M5)	$[M+Na]^+$	851.5676	MonomethylPhosphatidylethanolamine
		PE-NMe(13M5/9D3)	$[M+Na]^+$	851.5676	MonomethylPhosphatidylethanolamine
		PE-NMe(11M5/9D5)	$[M+Na]^+$	851.5676	MonomethylPhosphatidylethanolamine
		PE-NMe(11M5/11D3)	$[M+Na]^+$	851.5676	MonomethylPhosphatidylethanolamine
		PE-NMe(11M3/11D5)	$[M+Na]^+$	851.5676	MonomethylPhosphatidylethanolamine
		PE-NMe(11D5/9M5)	$[M+Na]^+$	851.5676	MonomethylPhosphatidylethanolamine

		PE-NMe(11D5/11M3)	$[M+Na]^+$	851.5676	MonomethylPhosphatidylethanolamine
		PE-NMe(11D3/11M5)	$[M+Na]^+$	851.5676	MonomethylPhosphatidylethanolamine
		PE(MonoMe(9,5)/MonoMe(13,5))	$[M+Na]^+$	851.5676	Phosphatidylethanolamine
		PE(MonoMe(13,5)/MonoMe(9,5))	$[M+Na]^+$	851.5676	Phosphatidylethanolamine
		PE(MonoMe(13,5)/MonoMe(11,3))	$[M+Na]^+$	851.5676	Phosphatidylethanolamine
		PE(MonoMe(11,5)/MonoMe(11,5))	$[M+Na]^+$	851.5676	Phosphatidylethanolamine
		PE(MonoMe(11,3)/MonoMe(13,5))	$[M+Na]^+$	851.5676	Phosphatidylethanolamine
		PE(DiMe(9,5)/DiMe(11,5))	$[M+Na]^+$	851.5676	Phosphatidylethanolamine
		PE(DiMe(9,3)/DiMe(13,5))	$[M+Na]^+$	851.5676	Phosphatidylethanolamine
		PE(DiMe(13,5)/DiMe(9,3))	$[M+Na]^+$	851.5676	Phosphatidylethanolamine
		PE(DiMe(11,5)/DiMe(9,5))	$[M+Na]^+$	851.5676	Phosphatidylethanolamine
		PE(DiMe(11,5)/DiMe(11,3))	$[M+Na]^+$	851.5676	Phosphatidylethanolamine
		PE(DiMe(11,3)/DiMe(11,5))	$[M+Na]^+$	851.5676	Phosphatidylethanolamine
		PC(MonoMe(9,5)/DiMe(9,5))	$[M+Na]^+$	852.5755	Phosphatidylcholine
		PC(MonoMe(9,5)/DiMe(11,3))	$[M+Na]^+$	852.5755	Phosphatidylcholine
		PC(MonoMe(11,5)/DiMe(9,3))	$[M+Na]^+$	852.5755	Phosphatidylcholine
		PC(MonoMe(11,3)/DiMe(9,5))	$[M+Na]^+$	852.5755	Phosphatidylcholine
		PC(DiMe(9,5)/MonoMe(9,5))	$[M+Na]^+$	852.5755	Phosphatidylcholine
		PC(MonoMe(11,3)/DiMe(11,3))	$[M+Na]^+$	852.5755	Phosphatidylcholine
		PC(DiMe(9,5)/MonoMe(11,3))	$[M+Na]^+$	852.5755	Phosphatidylcholine
		PC(DiMe(9,3)/MonoMe(11,5))	$[M+Na]^+$	852.5755	Phosphatidylcholine
		PC(DiMe(11,3)/MonoMe(9,5))	$[M+Na]^+$	852.5755	Phosphatidylcholine
		PC(DiMe(11,3)/MonoMe(11,3))	$[M+Na]^+$	852.5755	Phosphatidylcholine
		PGP(18:1(9Z)/18:0)	$[M+NH_4]^+$	856.5231	Glycerophospholipid
		PGP(18:1(11Z)/18:0)	$[M+NH_4]^+$	856.5231	Glycerophospholipid
		PGP(18:0/18:1(9Z))	$[M+NH_4]^+$	856.5231	Glycerophospholipid
		PGP(18:0/18:1(11Z))	$[M+NH_4]^+$	856.5231	Glycerophospholipid
399.1776	6.18	18-Oxocortisol	$[M+Na]^+$	376.1886	21-hydroxysteroids

116.0708	0.60	Proline	$[M+H]^+$	115.0633	Amino acid
311.1463	3.41	4-Hydroxyproline galactoside	$[M+NH_4]^+$	293.1111	Fatty alcohol esters
		Galactosyl 4-hydroxyproline	$[M+NH_4]^+$	293.1111	Alpha-amino acyl ester of carbohydrates
		N-Acetylmuramate	$[M+NH_4]^+$	293.1111	Acylaminosugar
		(3R)-3,4-Dihydroxy-3-(hydroxymethyl)butanenitrile 4-glucoside	$[M+NH_4]^+$	293.1111	Glycosylglycerols
176.0706	4.16	5-Hydroxyindoleacetaldehyde	$[M+H]^+$	175.0633	Hydroxyindoles – Found in rhubarbs, black radish, oriental wheat, garden tomato, wild leeks. Involved in tryptophan metabolism. Can be biosynthesized from serotonin through kynurenine 3-monooxygenase. Biogenic aldehyde of serotonin
		Indoleacetic acid	$[M+H]^+$	175.0633	Breakdown product of tryptophan metabolism and is often produced by bacteria in mammalian gut. Higher levels associated with bacteria from Clostridium species

Note: * = Clear peak, but peak shape is subpar

Table S6: Complete List of Tentatively Identified Exogenous Metabolites in Moderate vs. High Models

m/z	RT	Tentative ID	Adduct	Monoisotopic Mass	Biological Significance
		(-)-Salsoline	$[M+NH_4]^+$	193.1103	Compound that crystallizes from alcohol solution. Used in medicine as antihypertensive agent. Found in male alcoholic inpatients' urine and lumbar cerebrospinal fluid when patients were still intoxicated after heavy alcohol debauch
		(S)-N-Methylsalsolinol	$[M+NH_4]^+$	193.1103	Tetrahydroisoquinolines – Found in anatidaes, chickens, pigs
		4-(Butylamino)benzoic acid	$[M+NH_4]^+$	193.1103	Aminobenzoic acids
		5,6-Dihydroxy-2-methylaminotetralin	$[M+NH_4]^+$	193.1103	Tetralins
		3-Hydroxy-3-phenylpentanamide	$[M+NH_4]^+$	193.1103	Phenylpropanes
		Methoxyphedrine	$[M+NH_4]^+$	193.1103	Alkyl-phenylketones
		Isobutamben	$[M+NH_4]^+$	193.1103	Benzoic acid esters
		Butyl 4-aminobenzoate	$[M+NH_4]^+$	193.1103	Benzoic acid esters
		2-Methylpropyl 2-aminobenzoate	$[M+NH_4]^+$	193.1103	Benzoic acid esters
		Ethyl N-ethylanthranilate	$[M+NH_4]^+$	193.1103	Benzoic acid esters
		Butyl 2-aminobenzoate	$[M+NH_4]^+$	193.1103	Benzoic acid esters – secondary metabolite
		2-Isopropylphenyl methylcarbamate	$[M+NH_4]^+$	193.1103	Cumenes
211.1441	3.90	3,4-Methylenedioxymethamphetamine	$[M+NH_4]^+$	193.1103	Benzodioxoles – Known as ecstasy or MDMA
		3,5-Diisopropylsalicylic acid	$[M+Na]^+$	222.1256	Salicylic acids
		3-Hexylsalicylic acid	$[M+Na]^+$	222.1256	Salicylic acids
		(+)-Dehydrovomifoliol	$[M+Na]^+$	222.1256	Sesquiterpenoids – Found in rice
245.1146	5.61	Hexyl salicylic acid	$[M+Na]^+$	222.1256	O-hydroxybenzoic acid esters

		3-Hydroxyibuprofen	$[M+Na]^+$	222.1256	Phenylpropanoic acids – Metabolite of ibuprofen, a nonsteroidal anti-inflammatory drug for relief of arthritis, fever, and pain
		2-Hydroxyibuprofen	$[M+Na]^+$	222.1256	Phenylpropanoic acids – Metabolite of ibuprofen, a nonsteroidal anti-inflammatory drug for relief of arthritis, fever, and pain
		1-Hydroxyibuprofen	$[M+Na]^+$	222.1256	Phenylpropanoic acids – Metabolite of ibuprofen, a nonsteroidal anti-inflammatory drug for relief of arthritis, fever, and pain
		Methyl 3-epi-4,5-didehydrojasmonate	$[M+Na]^+$	222.1256	Methyl esters
		Annuionone B	$[M+Na]^+$	222.1256	Oxepanes – Found in fats, oils, sunflowers
		Isoamyl p-anisate	$[M+Na]^+$	222.1256	P-methoxybenzoic acids and derivatives
		Dehydrovomifoliol	$[M+Na]^+$	222.1256	Sesquiterpenoids
		2-Carboxy-5,7-dimethyl-4-octanolide	$[M+H]^+$	214.1205	Gamma butyrolactones
		5-Hexyltetrahydro-2-oxo-3-furancarboxylic acid	$[M+H]^+$	214.1205	Gamma butyrolactones
215.1279	4.78	alpha-Carboxy-delta-decalactone	$[M+H]^+$	214.1205	Delta valerolactones
415.2536	3.97	Methyl N-({(2s,3s)-3-[(Propylamino)carbonyl]oxiran-2-Yl}carbonyl)-L-Isoleucyl-L-Prolinate	$[M+NH_4]^+$	397.2213	Peptides
		Butanedioic acid, octenyl-	$[M+H]^+$	228.1362	Fatty acid esters
		5-Heptyltetrahydro-2-oxo-3-furancarboxylic acid	$[M+H]^+$	228.1362	Gamma butyrolactones
229.1433	5.30	Traumatic acid	$[M+H]^+$	228.1362	Monounsaturated dicarboxylic acid – Fount in plants. Potent wound healing agent in plants
		Ginsenoside Rg3	$[M+NH_4]^+$	784.4973	Triterpenoids
802.5350	19.32	Ginsenoside C	$[M+NH_4]^+$	784.4973	Triterpenoids

		Ginsenoside F2	$[M+NH_4]^+$	784.4973	Triterpenoids
356.2922	14.92	2,4,12-Octadecatrienoic acid isobutylamide	$[M+Na]^+$	333.3032	N-acyl amides – Found in Javanese long pepper
		Acarbosa	$[M+H]^+$	645.2480	Aminocyclitol glycosides
		Alpha-Acarbose	$[M+H]^+$	645.2480	Aminocyclitol glycosides
646.2575	18.76	Acarbose	$[M+H]^+$	645.2480	Aminocyclitol glycosides – Drug that inhibits alpha glucosidase that retards the digestion and absorption of carbohydrates in the small intestine, hence reducing the increase in blood-glucose concentration after a carbohydrate load.
159.1168	5.53	1-Phenyl-1-cyclohexene	$[M+H]^+$	158.1096	Benzene and substituted derivatives
544.3373	13.35	(S)-1-(Sec-Butyl)-N-((4,6-dimethyl-2-oxo-1,2-dihydropyridin-3-yl)methyl)-3-methyl-6-(6-(piperazin-1-yl)pyridin-3-yl)-1H-indole-4-carboxamide	$[M+NH_4]^+$	526.3056245	Pyridinylpiperazines
		4-Chlorobenzaldehyde	$[M+Na]^+$	140.0029	Benzoyl derivatives
		Benzoyl chloride	$[M+Na]^+$	140.0029	Benzoic acids and derivatives
162.9914	0.50	3-chlorobenzaldehyde	$[M+Na]^+$	140.0029	Benzoyl derivatives – found in calabash, black raspberry, ginger, tartary buckwheat
381.2245	10.00	Pracinostat	$[M+Na]^+$	358.2369	Benzimidazoles
518.3215	12.95	Laprafylline	$[M+NH_4]^+$	500.2900	diphenylmethanes
		Avocadene 1-acetate	$[M+Na]^+$	328.2614	Long-chain fatty alcohols
		Avocadene 4-acetate	$[M+Na]^+$	328.2614	Long-chain fatty alcohols
351.2504	12.67	Avocadene 2-acetate	$[M+Na]^+$	328.2614	Long-chain fatty alcohols
		10,12-Tetracosanedione	$[M+NH_4]^+$	366.3498	Beta-diketones
		4,6-Tetracosanedione	$[M+NH_4]^+$	366.3498	Beta-diketones – Found in fats and oils
		5,7-Tetracosanedione	$[M+NH_4]^+$	366.3498	Beta-diketones – Found in fats and oils
384.3833*	13.74	7,9-Tetracosanedione	$[M+NH_4]^+$	366.3498	Beta-diketones – Found in fats and oils

		(E)-2-Tetracosenoic acid	$[M+NH_4]^+$	366.3498	Very long-chain fatty acids
		6,8-Tetracosanedione	$[M+NH_4]^+$	366.3498	Beta-diketones
		Benzoylarginine nitroanilide	$[M+H]^+$	398.1703	Hippuric acids and derivatives
		16alpha-Hydroxyprednisolone	$[M+Na]^+$	376.1886	21-hydroxysteroids - Metabolite of budesonide which is a glucocorticoid steroid for treatment of asthma, non-infectious rhinitis, nasal polyposis and Crohn's disease
		1,7-bis-(4-Hydroxy-3-methoxyphenyl)-heptane-3,5-diol	$[M+Na]^+$	376.1886	Curcuminoids – Found in ginger
		12alpha-Hydroxy-13,18-dehydroparain	$[M+Na]^+$	376.1886	Quassinoids
399.1776	6.18	11,12,14-Trihydroxy-7-methoxy-8,11,13-abietatrien-20,6-olide	$[M+Na]^+$	376.1886	Diterpene lactones
		N-Allylglycine	$[M+H]^+$	115.0633	Alpha amino acids
		2-Pentenedial	$[M+NH_4]^+$	98.0368	Enals
		5-Hydroxy-1-methylpyrrolidin-2-one	$[M+H]^+$	115.0633	N-alkylpyrrolidines
		N-(2-Hydroxyethyl)acrylamide	$[M+H]^+$	115.0633	(AKA PNHEA or polyduramide) N-acylethanolamines
		5-Methyl-3(2H)-furanone	$[M+NH_4]^+$	98.0368	Furanones
		alpha-Methylene-gamma-butyrolactone	$[M+NH_4]^+$	98.0368	Gamma butyrolactones
		DL-Allylglycine	$[M+H]^+$	115.0633	Alpha amino acids
		Acetamidopropanal	$[M+H]^+$	115.0633	Acetamidopropanal is associated with urea cycle and metabolism of arginine, proline, glutamate, aspartate and asparagine
		2-Furanmethanol	$[M+NH_4]^+$	98.0368	Heteroaromatic compounds – Found in cereals, potato, white mustards, arabica coffee, cocoa
116.0708	0.60	5-Methyl-2(3H)-furanone	$[M+NH_4]^+$	98.0368	Butenolides – Found in blackberries

		Pterolactam	$[M+H]^+$	115.0633	Pyrrolidines – Found in green vegetables and root vegetables
		5-Hydroxy-4-pentenoic acid d-lactone	$[M+NH_4]^+$	98.0368	Dihydropyranones – Found in nuts
		4-Amino-2-methylenebutanoic acid	$[M+H]^+$	115.0633	Gamma amino acids and derivatives
		Neoacrimarine F	$[M+NH_4]^+$	531.1529	Triacylglycerols
549.1857	16.38	Neoacrimarine I	$[M+NH_4]^+$	531.1529	Acridones – Found in citrus
		2H-1-Benzopyran-2-one, aminomethyl-	$[M+H]^+$	175.0633	Coumarins and derivatives
		indole-3-glycol aldehyde	$[M+H]^+$	175.0633	3-alkylindoles – Found in tree fern, jostaberry, pitanga, pine nut
		3-Acetoxyindole	$[M+H]^+$	175.0633	Indoles
		1,2-Naphthoquinone	$[M+NH_4]^+$	158.0368	Naphthoquinones – primary metabolite
		Gentianine	$[M+H]^+$	175.0633	Pyranopyridines – Found in fenugreek
		Hydroxymethyl indol-3-yl ketone	$[M+H]^+$	175.0633	Indoles – Found in mushrooms
		7-Amino-4-methylcoumarin	$[M+H]^+$	175.0633	Coumarins and derivatives
		1,4-Naphthoquinone	$[M+NH_4]^+$	158.0368	Naphthoquinones – primary metabolite
176.0706	4.16	1-Methoxy-1H-indole-3-carboxaldehyde	$[M+H]^+$	175.0633	Indoles – Found in broccolis
		2-[(2,6-Dioxocyclohexyl)methyl]cyclohexane-1,3-dione	$[M+Na]^+$	236.1049	Beta-diketones
		Carboxy-ibuprofen	$[M+Na]^+$	236.1049	Phenylpropanoic acid – Nonsteroidal anti-inflammatory drug used for relief of symptoms of arthritis, fever, pain. Metabolite of ibuprofen. Can be biosynthesized from 3-hydroxyibuprofen via Cytochrome P450 2c9
259.0938	5.55	Ethyl vanillin isobutyrate	$[M+Na]^+$	236.1049	Phenol esters

Note: * = Clear peak, but peak shape is subpar

Table S7: Complete List of Tentatively Identified Endogenous Metabolites in Low vs. Moderate Models

m/z	RT	Tentative ID	Adduct	Monoisotopic Mass	Biological Significance
311.1464	3.40	4-Hydroxyproline galactoside	$[M+NH_4]^+$	293.1111	Fatty alcohol esters
		Galactosyl 4-hydroxyproline	$[M+NH_4]^+$	293.1111	Alpha-amino acyl ester of carbohydrates
		N-Acetylmuramate	$[M+NH_4]^+$	293.1111	Acylaminosugar – Ubiquitous in all organisms
		(3R)-3,4-Dihydroxy-3-(hydroxymethyl)butanenitrile 4-glucoside	$[M+NH_4]^+$	293.1111	Glycosylglycerols
524.3710	14.21	Deoxycholylasparagine	$[M+NH_4]^+$	506.3356	Bile amino acid conjugates – Consists of deoxycholic acid conjugated to Asparagine at C24 acyl site. Produced by gut microbiota – Secondary bile acid. Increased in patients with Inflammatory Bowel Disease, Cystic Fibrosis, and in infants. Act as agonist for farnesoid X receptor leading to reduced expression of bile acid synthesis genes
		Chenodeoxycholylasparagine	$[M+NH_4]^+$	506.3356	Bile amino acid conjugates – Consists of Chenodeoxycholic acid conjugated to Asparagine at C24 acyl site. Produced by gut microbiota – Secondary bile acids. Increased in patients with Inflammatory Bowel Disease, Cystic Fibrosis, and in infants. Act as agonist for farnesoid X receptor

					leading to reduced expression of bile acid synthesis genes
		LysoPC(0:0/18:0)	[M+H]+	523.3638	Lysophospholipid
		LysoPC(18:0/0:0)	[M+H]+	523.3638	Lysophospholipid
		Platelet-activating factor	[M+H]+	523.3638	Ubiquitous, potent phospholipid activator and mediator of inflammation
263.0887	6.70	3-Carboxy-4-methyl-5-propyl-2-furanpropionic acid	[M+Na]+	240.0998	Uremic Toxin – Significantly accumulated in serum of chronic kidney disease patients. Formed from consumption of fish, vegetables, fruits. Strong inhibitor of mitochondrial respiration, associated with thyroid dysfunction. Directly inhibits renal secretion of various drugs and endogenous organic acids by competitively inhibiting OAT3 transporters. Also inhibits organic acid transport at blood-brain-barrier
		N-Acetyl-serylaspartic acid	[M+H]+	262.0801	Dipeptide
		Glutamylaspartic acid	[M+H]+	262.0801	Dipeptide – Incompelete breakdown product of protein digestion or catabolism. Found in pulses and soybean
		Aspartyl-Glutamate	[M+H]+	262.0801	Dipeptide – Incomplete breakdown product of protein digestion or catabolism
		gamma-Glutamylaspartic acid	[M+H]+	262.0801	Dipeptide – Incomplete breakdown product of protein digestion or catabolism. Found in pulses

		Cer(d16:1/TXB2)	$[M+NH_4]^+$	623.4761	Sphingolipid/glycosylceramide
		Cer(d16:1/6 keto-PGF1alpha)	$[M+NH_4]^+$	623.4761	Sphingolipid/glycosylceramide
		DG(16:0/20:3(11Z,14Z,17Z)/0:0)	$[M+Na]^+$	618.5223	Diacylglycerol
		DG(16:1n7/0:0/20:2n6)	$[M+Na]^+$	618.5223	Diacylglycerol
		DG(14:1n5/0:0/22:2n6)	$[M+Na]^+$	618.5223	Diacylglycerol
		DG(18:0/0:0/18:3n3)	$[M+Na]^+$	618.5223	Diacylglycerol
		DG(18:0/0:0/18:3n6)	$[M+Na]^+$	618.5223	Diacylglycerol
		DG(16:0/0:0/20:3n6)	$[M+Na]^+$	618.5223	Diacylglycerol
		DG(16:0/0:0/20:3n9)	$[M+Na]^+$	618.5223	Diacylglycerol
		DG(20:2n6/0:0/18:4n3)	$[M+H]^+$	640.5067	Diacylglycerol
		DG(20:3n6/0:0/18:3n3)	$[M+H]^+$	640.5067	Diacylglycerol
		DG(18:3n6/0:0/20:3n6)	$[M+H]^+$	640.5067	Diacylglycerol
		DG(20:3n9/0:0/18:3n3)	$[M+H]^+$	640.5067	Diacylglycerol
		DG(20:3n9/0:0/18:3n6)	$[M+H]^+$	640.5067	Diacylglycerol
		DG(18:1n9/0:0/20:5n3)	$[M+H]^+$	640.5067	Diacylglycerol
		DG(18:1n7/0:0/20:5n3)	$[M+H]^+$	640.5067	Diacylglycerol
		DG(16:1n7/0:0/22:5n3)	$[M+H]^+$	640.5067	Diacylglycerol
		DG(16:1n7/0:0/22:5n6)	$[M+H]^+$	640.5067	Diacylglycerol
		DG(16:0/0:0/22:6n3)	$[M+H]^+$	640.5067	Diacylglycerol
		DG(22:6(4Z,7Z,10Z,13Z,16Z,19Z)/16:0/0:0)	$[M+H]^+$	640.5067	Diacylglycerol
		DG(22:5(7Z,10Z,13Z,16Z,19Z)/16:1(9Z)/0:0)	$[M+H]^+$	640.5067	Diacylglycerol
		DG(22:5(4Z,7Z,10Z,13Z,16Z)/16:1(9Z)/0:0)	$[M+H]^+$	640.5067	Diacylglycerol
		DG(22:2(13Z,16Z)/14:1(9Z)/0:0)	$[M+Na]^+$	618.5223	Diacylglycerol
		DG(20:5(5Z,8Z,11Z,14Z,17Z)/18:1(9Z)/0:0)	$[M+H]^+$	640.5067	Diacylglycerol
		DG(20:5(5Z,8Z,11Z,14Z,17Z)/18:1(11Z)/0:0)	$[M+H]^+$	640.5067	Diacylglycerol
		DG(20:4(8Z,11Z,14Z,17Z)/18:2(9Z,12Z)/0:0)	$[M+H]^+$	640.5067	Diacylglycerol
		DG(20:4(5Z,8Z,11Z,14Z)/18:2(9Z,12Z)/0:0)	$[M+H]^+$	640.5067	Diacylglycerol
		DG(20:3(8Z,11Z,14Z)/18:3(9Z,12Z,15Z)/0:0)	$[M+H]^+$	640.5067	Diacylglycerol
		DG(20:3(8Z,11Z,14Z)/18:3(6Z,9Z,12Z)/0:0)	$[M+H]^+$	640.5067	Diacylglycerol
641.5110	20.65	DG(20:3(8Z,11Z,14Z)/16:0/0:0)	$[M+Na]^+$	618.5223	Diacylglycerol

		DG(20:3(5Z,8Z,11Z)/18:3(9Z,12Z,15Z)/0:0)	[M+H]+	640.5067	Diacylglycerol
		DG(20:3(5Z,8Z,11Z)/18:3(6Z,9Z,12Z)/0:0)	[M+H]+	640.5067	Diacylglycerol
		DG(20:3(5Z,8Z,11Z)/16:0/0:0)	[M+Na]+	618.5223	Diacylglycerol
		DG(20:2(11Z,14Z)/18:4(6Z,9Z,12Z,15Z)/0:0)	[M+H]+	640.5067	Diacylglycerol
		DG(20:2(11Z,14Z)/16:1(9Z)/0:0)	[M+Na]+	618.5223	Diacylglycerol
		DG(18:4(6Z,9Z,12Z,15Z)/20:2(11Z,14Z)/0:0)	[M+H]+	640.5067	Diacylglycerol
		DG(18:3(9Z,12Z,15Z)/20:3(8Z,11Z,14Z)/0:0)	[M+H]+	640.5067	Diacylglycerol
		DG(18:3(9Z,12Z,15Z)/20:3(5Z,8Z,11Z)/0:0)	[M+H]+	640.5067	Diacylglycerol
		DG(18:3(9Z,12Z,15Z)/18:0/0:0)	[M+Na]+	618.5223	Diacylglycerol
		DG(18:3(6Z,9Z,12Z)/20:3(8Z,11Z,14Z)/0:0)	[M+H]+	640.5067	Diacylglycerol
		DG(18:3(6Z,9Z,12Z)/20:3(5Z,8Z,11Z)/0:0)	[M+H]+	640.5067	Diacylglycerol
		DG(18:3(6Z,9Z,12Z)/18:0/0:0)	[M+Na]+	618.5223	Diacylglycerol
		DG(18:2(9Z,12Z)/20:4(8Z,11Z,14Z,17Z)/0:0)	[M+H]+	640.5067	Diacylglycerol
		DG(18:2(9Z,12Z)/20:4(5Z,8Z,11Z,14Z)/0:0)	[M+H]+	640.5067	Diacylglycerol
		DG(18:2(9Z,12Z)/18:1(11Z)/0:0)	[M+Na]+	618.5223	Diacylglycerol
		DG(18:2(9Z,12Z)/18:1(9Z)/0:0)	[M+Na]+	618.5223	Diacylglycerol
		DG(18:1(9Z)/20:5(5Z,8Z,11Z,14Z,17Z)/0:0)	[M+H]+	640.5067	Diacylglycerol
		DG(18:1(9Z)/18:2(9Z,12Z)/0:0)	[M+Na]+	618.5223	Diacylglycerol
		DG(18:1(11Z)/20:5(5Z,8Z,11Z,14Z,17Z)/0:0)	[M+H]+	640.5067	Diacylglycerol
		DG(18:1(11Z)/18:2(9Z,12Z)/0:0)	[M+Na]+	618.5223	Diacylglycerol
		DG(18:0/18:3(6Z,9Z,12Z)/0:0)	[M+Na]+	618.5223	Diacylglycerol
		DG(18:0/18:3(9Z,12Z,15Z)/0:0)	[M+Na]+	618.5223	Diacylglycerol
		DG(16:1(9Z)/22:5(7Z,10Z,13Z,16Z,19Z)/0:0)	[M+H]+	640.5067	Diacylglycerol
		DG(16:1(9Z)/22:5(4Z,7Z,10Z,13Z,16Z)/0:0)	[M+H]+	640.5067	Diacylglycerol
		DG(16:1(9Z)/20:2(11Z,14Z)/0:0)	[M+Na]+	618.5223	Diacylglycerol
		DG(16:0/22:6(4Z,7Z,10Z,13Z,16Z,19Z)/0:0)	[M+H]+	640.5067	Diacylglycerol
		DG(16:0/20:3(8Z,11Z,14Z)/0:0)	[M+Na]+	618.5223	Diacylglycerol
		DG(16:0/20:3(5Z,8Z,11Z)/0:0)	[M+Na]+	618.5223	Diacylglycerol
		DG(14:1(9Z)/22:2(13Z,16Z)/0:0)	[M+Na]+	618.5223	Diacylglycerol

		PC(20:4(5Z,7E,11Z,14Z)-OH(9)/P-18:1(9Z))	[M+Na]+	807.5778	Oxidized Phosphatidylcholines - signaling molecules on cell membrane
		PC(P-18:1(9Z)/20:4(5Z,7E,11Z,14Z)-OH(9))	[M+Na]+	807.5778	Oxidized Phosphatidylcholines - signaling molecules on cell membrane
		PC(20:4(5E,8Z,12Z,14Z)-OH(11R)/P-18:1(9Z))	[M+Na]+	807.5778	Oxidized Phosphatidylcholines - signaling molecules on cell membrane
		PC(P-18:1(9Z)/20:4(5E,8Z,12Z,14Z)-OH(11R))	[M+Na]+	807.5778	Oxidized Phosphatidylcholines - signaling molecules on cell membrane
		PC(20:4(5Z,8Z,10E,14Z)-OH(12S)/P-18:1(9Z))	[M+Na]+	807.5778	Oxidized Phosphatidylcholines - signaling molecules on cell membrane
		PC(P-18:1(9Z)/20:4(5Z,8Z,10E,14Z)-OH(12S))	[M+Na]+	807.5778	Oxidized Phosphatidylcholines - signaling molecules on cell membrane
		PC(20:4(5Z,8Z,11Z,13E)-OH(15S)/P-18:1(9Z))	[M+Na]+	807.5778	Oxidized Phosphatidylcholines - signaling molecules on cell membrane
		PC(P-18:1(9Z)/20:4(5Z,8Z,11Z,13E)-OH(15S))	[M+Na]+	807.5778	Oxidized Phosphatidylcholines - signaling molecules on cell membrane
		PC(20:4(5Z,8Z,11Z,14Z)-OH(16R)/P-18:1(9Z))	[M+Na]+	807.5778	Oxidized Phosphatidylcholines - signaling molecules on cell membrane
830.5664	19.88	PC(P-18:1(9Z)/20:4(5Z,8Z,11Z,14Z)-OH(16R))	[M+Na]+	807.5778	Oxidized Phosphatidylcholines - signaling molecules on cell membrane

		PC(20:4(5Z,8Z,11Z,14Z)-OH(17)/P-18:1(9Z))	[M+Na]+	807.5778	Oxidized Phosphatidylcholines - signaling molecules on cell membrane
		PC(P-18:1(9Z)/20:4(5Z,8Z,11Z,14Z)-OH(17))	[M+Na]+	807.5778	Oxidized Phosphatidylcholines - signaling molecules on cell membrane
		PC(20:4(5Z,8Z,11Z,14Z)-OH(18R)/P-18:1(9Z))	[M+Na]+	807.5778	Oxidized Phosphatidylcholines - signaling molecules on cell membrane
		PC(P-18:1(9Z)/20:4(5Z,8Z,11Z,14Z)-OH(18R))	[M+Na]+	807.5778	Oxidized Phosphatidylcholines - signaling molecules on cell membrane
		PC(20:4(5Z,8Z,11Z,14Z)-OH(19S)/P-18:1(9Z))	[M+Na]+	807.5778	Oxidized Phosphatidylcholines - signaling molecules on cell membrane
		PC(P-18:1(9Z)/20:4(5Z,8Z,11Z,14Z)-OH(19S))	[M+Na]+	807.5778	Oxidized Phosphatidylcholines - signaling molecules on cell membrane
		PC(20:4(6E,8Z,11Z,14Z)-OH(5S)/P-18:1(9Z))	[M+Na]+	807.5778	Oxidized Phosphatidylcholines - signaling molecules on cell membrane
		PC(P-18:1(9Z)/20:4(6E,8Z,11Z,14Z)-OH(5S))	[M+Na]+	807.5778	Oxidized Phosphatidylcholines - signaling molecules on cell membrane
		PC(20:4(5Z,8Z,11Z,14Z)-OH(20)/P-18:1(9Z))	[M+Na]+	807.5778	Oxidized Phosphatidylcholines - signaling molecules on cell membrane
		PC(P-18:1(9Z)/20:4(5Z,8Z,11Z,14Z)-OH(20))	[M+Na]+	807.5778	Oxidized Phosphatidylcholines - signaling molecules on cell membrane

		PC(20:3(8Z,11Z,14Z)-O(5,6)/P-18:1(9Z))	$[M+Na]^+$	807.5778	Oxidized Phosphatidylcholines - signaling molecules on cell membrane
		PC(P-18:1(9Z)/20:3(8Z,11Z,14Z)-O(5,6))	$[M+Na]^+$	807.5778	Oxidized Phosphatidylcholines - signaling molecules on cell membrane
		PC(20:3(5Z,11Z,14Z)-O(8,9)/P-18:1(9Z))	$[M+Na]^+$	807.5778	Oxidized Phosphatidylcholines - signaling molecules on cell membrane
		PC(P-18:1(9Z)/20:3(5Z,11Z,14Z)-O(8,9))	$[M+Na]^+$	807.5778	Oxidized Phosphatidylcholines - signaling molecules on cell membrane
		PC(20:3(5Z,8Z,14Z)-O(11S,12R)/P-18:1(9Z))	$[M+Na]^+$	807.5778	Oxidized Phosphatidylcholines - signaling molecules on cell membrane
		PC(P-18:1(9Z)/20:3(5Z,8Z,14Z)-O(11S,12R))	$[M+Na]^+$	807.5778	Oxidized Phosphatidylcholines - signaling molecules on cell membrane
		PC(20:3(5Z,8Z,11Z)-O(14R,15S)/P-18:1(9Z))	$[M+Na]^+$	807.5778	Oxidized Phosphatidylcholines - signaling molecules on cell membrane
		PC(P-18:1(9Z)/20:3(5Z,8Z,11Z)-O(14R,15S))	$[M+Na]^+$	807.5778	Oxidized Phosphatidylcholines - signaling molecules on cell membrane
		PC(20:4(5Z,7E,11Z,14Z)-OH(9)/P-18:1(11Z))	$[M+Na]^+$	807.5778	Oxidized Phosphatidylcholines - signaling molecules on cell membrane
		PC(P-18:1(11Z)/20:4(5Z,7E,11Z,14Z)-OH(9))	$[M+Na]^+$	807.5778	Oxidized Phosphatidylcholines - signaling molecules on cell membrane

		PC(20:4(5E,8Z,12Z,14Z)-OH(11R)/P-18:1(11Z))	$[M+Na]^+$	807.5778	Oxidized Phosphatidylcholines - signaling molecules on cell membrane
		PC(P-18:1(11Z)/20:4(5E,8Z,12Z,14Z)-OH(11R))	$[M+Na]^+$	807.5778	Oxidized Phosphatidylcholines - signaling molecules on cell membrane
		PC(20:4(5Z,8Z,10E,14Z)-OH(12S)/P-18:1(11Z))	$[M+Na]^+$	807.5778	Oxidized Phosphatidylcholines - signaling molecules on cell membrane
		PC(P-18:1(11Z)/20:4(5Z,8Z,10E,14Z)-OH(12S))	$[M+Na]^+$	807.5778	Oxidized Phosphatidylcholines - signaling molecules on cell membrane
		PC(20:4(5Z,8Z,11Z,13E)-OH(15S)/P-18:1(11Z))	$[M+Na]^+$	807.5778	Oxidized Phosphatidylcholines - signaling molecules on cell membrane
		PC(P-18:1(11Z)/20:4(5Z,8Z,11Z,13E)-OH(15S))	$[M+Na]^+$	807.5778	Oxidized Phosphatidylcholines - signaling molecules on cell membrane
		PC(20:4(5Z,8Z,11Z,14Z)-OH(16R)/P-18:1(11Z))	$[M+Na]^+$	807.5778	Oxidized Phosphatidylcholines - signaling molecules on cell membrane
		PC(P-18:1(11Z)/20:4(5Z,8Z,11Z,14Z)-OH(16R))	$[M+Na]^+$	807.5778	Oxidized Phosphatidylcholines - signaling molecules on cell membrane
		PC(20:4(5Z,8Z,11Z,14Z)-OH(17)/P-18:1(11Z))	$[M+Na]^+$	807.5778	Oxidized Phosphatidylcholines - signaling molecules on cell membrane
		PC(P-18:1(11Z)/20:4(5Z,8Z,11Z,14Z)-OH(17))	$[M+Na]^+$	807.5778	Oxidized Phosphatidylcholines - signaling molecules on cell membrane

		PC(20:4(5Z,8Z,11Z,14Z)-OH(18R)/P-18:1(11Z))	[M+Na]+	807.5778	Oxidized Phosphatidylcholines - signaling molecules on cell membrane
		PC(P-18:1(11Z)/20:4(5Z,8Z,11Z,14Z)-OH(18R))	[M+Na]+	807.5778	Oxidized Phosphatidylcholines - signaling molecules on cell membrane
		PC(20:4(5Z,8Z,11Z,14Z)-OH(19S)/P-18:1(11Z))	[M+Na]+	807.5778	Oxidized Phosphatidylcholines - signaling molecules on cell membrane
		PC(P-18:1(11Z)/20:4(5Z,8Z,11Z,14Z)-OH(19S))	[M+Na]+	807.5778	Oxidized Phosphatidylcholines - signaling molecules on cell membrane
		PC(20:4(6E,8Z,11Z,14Z)-OH(5S)/P-18:1(11Z))	[M+Na]+	807.5778	Oxidized Phosphatidylcholines - signaling molecules on cell membrane
		PC(P-18:1(11Z)/20:4(6E,8Z,11Z,14Z)-OH(5S))	[M+Na]+	807.5778	Oxidized Phosphatidylcholines - signaling molecules on cell membrane
		PC(20:4(5Z,8Z,11Z,14Z)-OH(20)/P-18:1(11Z))	[M+Na]+	807.5778	Oxidized Phosphatidylcholines - signaling molecules on cell membrane
		PC(P-18:1(11Z)/20:4(5Z,8Z,11Z,14Z)-OH(20))	[M+Na]+	807.5778	Oxidized Phosphatidylcholines - signaling molecules on cell membrane
		PC(20:3(8Z,11Z,14Z)-O(5,6)/P-18:1(11Z))	[M+Na]+	807.5778	Oxidized Phosphatidylcholines - signaling molecules on cell membrane
		PC(P-18:1(11Z)/20:3(8Z,11Z,14Z)-O(5,6))	[M+Na]+	807.5778	Oxidized Phosphatidylcholines - signaling molecules on cell membrane

		PC(20:3(5Z,11Z,14Z)-O(8,9)/P-18:1(11Z))	$[M+Na]^+$	807.5778	Oxidized Phosphatidylcholines - signaling molecules on cell membrane
		PC(P-18:1(11Z)/20:3(5Z,11Z,14Z)-O(8,9))	$[M+Na]^+$	807.5778	Oxidized Phosphatidylcholines - signaling molecules on cell membrane
		PC(20:3(5Z,8Z,14Z)-O(11S,12R)/P-18:1(11Z))	$[M+Na]^+$	807.5778	Oxidized Phosphatidylcholines - signaling molecules on cell membrane
		PC(P-18:1(11Z)/20:3(5Z,8Z,14Z)-O(11S,12R))	$[M+Na]^+$	807.5778	Oxidized Phosphatidylcholines - signaling molecules on cell membrane
		PC(20:3(5Z,8Z,11Z)-O(14R,15S)/P-18:1(11Z))	$[M+Na]^+$	807.5778	Oxidized Phosphatidylcholines - signaling molecules on cell membrane
		PC(P-18:1(11Z)/20:3(5Z,8Z,11Z)-O(14R,15S))	$[M+Na]^+$	807.5778	Oxidized Phosphatidylcholines - signaling molecules on cell membrane
		PC(20:5(6E,8Z,11Z,14Z,17Z)-OH(5)/P-18:0)	$[M+Na]^+$	807.5778	Oxidized Phosphatidylcholines - signaling molecules on cell membrane
		PC(P-18:0/20:5(6E,8Z,11Z,14Z,17Z)-OH(5))	$[M+Na]^+$	807.5778	Oxidized Phosphatidylcholines - signaling molecules on cell membrane
		PC(20:5(5Z,8Z,10E,14Z,17Z)-OH(12)/P-18:0)	$[M+Na]^+$	807.5778	Oxidized Phosphatidylcholines - signaling molecules on cell membrane
		PC(P-18:0/20:5(5Z,8Z,10E,14Z,17Z)-OH(12))	$[M+Na]^+$	807.5778	Oxidized Phosphatidylcholines - signaling molecules on cell membrane

		PC(20:5(5Z,8Z,11Z,14Z,16E)-OH(18)/P-18:0)	$[M+Na]^+$	807.5778	Oxidized Phosphatidylcholines - signaling molecules on cell membrane
		PC(P-18:0/20:5(5Z,8Z,11Z,14Z,16E)-OH(18))	$[M+Na]^+$	807.5778	Oxidized Phosphatidylcholines - signaling molecules on cell membrane
		PC(20:5(5Z,8Z,11Z,14Z,16E)-OH(18R)/P-18:0)	$[M+Na]^+$	807.5778	Oxidized Phosphatidylcholines - signaling molecules on cell membrane
		PC(P-18:0/20:5(5Z,8Z,11Z,14Z,16E)-OH(18R))	$[M+Na]^+$	807.5778	Oxidized Phosphatidylcholines - signaling molecules on cell membrane
		PC(20:4(5Z,8Z,11Z,13E)+=O(15)/P-18:0)	$[M+Na]^+$	807.5778	Oxidized Phosphatidylcholines - signaling molecules on cell membrane
		PC(P-18:0/20:4(5Z,8Z,11Z,13E)+=O(15))	$[M+Na]^+$	807.5778	Oxidized Phosphatidylcholines - signaling molecules on cell membrane
		PC(20:4(6E,8Z,11Z,14Z)+=O(5)/P-18:0)	$[M+Na]^+$	807.5778	Oxidized Phosphatidylcholines - signaling molecules on cell membrane
		PC(P-18:0/20:4(6E,8Z,11Z,14Z)+=O(5))	$[M+Na]^+$	807.5778	Oxidized Phosphatidylcholines - signaling molecules on cell membrane
		PE-NMe(20:3(5Z,8Z,11Z)/20:2(11Z,14Z))	$[M+Na]^+$	807.5778	MonomethylPhosphatidylethanolamine - formed by methylation of Phosphatidylethanolamine as part of biosynthesis of Phosphatidylcholine. Usually found at trace levels

		PE-NMe(20:3(8Z,11Z,14Z)/20:2(11Z,14Z))	$[M+Na]^+$	807.5778	MonomethylPhosphatidylethanolamine - formed by methylation of Phosphatidylethanolamine as part of biosynthesis of Phosphatidylcholine. Usually found at trace levels
		PE-NMe(20:2(11Z,14Z)/20:3(8Z,11Z,14Z))	$[M+Na]^+$	807.5778	MonomethylPhosphatidylethanolamine - formed by methylation of Phosphatidylethanolamine as part of biosynthesis of Phosphatidylcholine. Usually found at trace levels
		PE-NMe(20:2(11Z,14Z)/20:3(5Z,8Z,11Z))	$[M+Na]^+$	807.5778	MonomethylPhosphatidylethanolamine - formed by methylation of Phosphatidylethanolamine as part of biosynthesis of Phosphatidylcholine. Usually found at trace levels
		PE-NMe(22:5(7Z,10Z,13Z,16Z,19Z)/18:0)	$[M+Na]^+$	807.5778	MonomethylPhosphatidylethanolamine - formed by methylation of Phosphatidylethanolamine as part of biosynthesis of Phosphatidylcholine. Usually found at trace levels
		PE-NMe(22:5(4Z,7Z,10Z,13Z,16Z)/18:0)	$[M+Na]^+$	807.5778	MonomethylPhosphatidylethanolamine - formed by methylation of Phosphatidylethanolamine as part of biosynthesis of Phosphatidylcholine. Usually found at trace levels

		PE-NMe(22:4(7Z,10Z,13Z,16Z)/18:1(9Z))	$[M+Na]^+$	807.5778	MonomethylPhosphatidylethanolamine - formed by methylation of Phosphatidylethanolamine as part of biosynthesis of Phosphatidylcholine. Usually found at trace levels
		PE-NMe(22:4(7Z,10Z,13Z,16Z)/18:1(11Z))	$[M+Na]^+$	807.5778	MonomethylPhosphatidylethanolamine - formed by methylation of Phosphatidylethanolamine as part of biosynthesis of Phosphatidylcholine. Usually found at trace levels
		PE-NMe(22:2(13Z,16Z)/18:3(6Z,9Z,12Z))	$[M+Na]^+$	807.5778	MonomethylPhosphatidylethanolamine - formed by methylation of Phosphatidylethanolamine as part of biosynthesis of Phosphatidylcholine. Usually found at trace levels
		PE-NMe(22:2(13Z,16Z)/18:3(9Z,12Z,15Z))	$[M+Na]^+$	807.5778	MonomethylPhosphatidylethanolamine - formed by methylation of Phosphatidylethanolamine as part of biosynthesis of Phosphatidylcholine. Usually found at trace levels
		PE-NMe(22:1(13Z)/18:4(6Z,9Z,12Z,15Z))	$[M+Na]^+$	807.5778	MonomethylPhosphatidylethanolamine - formed by methylation of Phosphatidylethanolamine as part of biosynthesis of Phosphatidylcholine. Usually found at trace levels

		PE-NMe(20:5(5Z,8Z,11Z,14Z,17Z)/20:0)	$[M+Na]^+$	807.5778	MonomethylPhosphatidylethanolamine - formed by methylation of Phosphatidylethanolamine as part of biosynthesis of Phosphatidylcholine. Usually found at trace levels
		PE-NMe(20:4(8Z,11Z,14Z,17Z)/20:1(11Z))	$[M+Na]^+$	807.5778	MonomethylPhosphatidylethanolamine - formed by methylation of Phosphatidylethanolamine as part of biosynthesis of Phosphatidylcholine. Usually found at trace levels
		PE-NMe(20:4(5Z,8Z,11Z,14Z)/20:1(11Z))	$[M+Na]^+$	807.5778	MonomethylPhosphatidylethanolamine - formed by methylation of Phosphatidylethanolamine as part of biosynthesis of Phosphatidylcholine. Usually found at trace levels
		PE-NMe(20:1(11Z)/20:4(5Z,8Z,11Z,14Z))	$[M+Na]^+$	807.5778	MonomethylPhosphatidylethanolamine - formed by methylation of Phosphatidylethanolamine as part of biosynthesis of Phosphatidylcholine. Usually found at trace levels
		PE-NMe(20:1(11Z)/20:4(8Z,11Z,14Z,17Z))	$[M+Na]^+$	807.5778	MonomethylPhosphatidylethanolamine - formed by methylation of Phosphatidylethanolamine as part of biosynthesis of Phosphatidylcholine. Usually found at trace levels

		PE-NMe(20:0/20:5(5Z,8Z,11Z,14Z,17Z))	$[M+Na]^+$	807.5778	MonomethylPhosphatidylethanolamine - formed by methylation of Phosphatidylethanolamine as part of biosynthesis of Phosphatidylcholine. Usually found at trace levels
		PE-NMe(18:4(6Z,9Z,12Z,15Z)/22:1(13Z))	$[M+Na]^+$	807.5778	MonomethylPhosphatidylethanolamine - formed by methylation of Phosphatidylethanolamine as part of biosynthesis of Phosphatidylcholine. Usually found at trace levels
		PE-NMe(18:3(9Z,12Z,15Z)/22:2(13Z,16Z))	$[M+Na]^+$	807.5778	MonomethylPhosphatidylethanolamine - formed by methylation of Phosphatidylethanolamine as part of biosynthesis of Phosphatidylcholine. Usually found at trace levels
		PE-NMe(18:3(6Z,9Z,12Z)/22:2(13Z,16Z))	$[M+Na]^+$	807.5778	MonomethylPhosphatidylethanolamine - formed by methylation of Phosphatidylethanolamine as part of biosynthesis of Phosphatidylcholine. Usually found at trace levels
		PE-NMe(18:1(9Z)/22:4(7Z,10Z,13Z,16Z))	$[M+Na]^+$	807.5778	MonomethylPhosphatidylethanolamine - formed by methylation of Phosphatidylethanolamine as part of biosynthesis of Phosphatidylcholine. Usually found at trace levels

		PE-NMe(18:1(11Z)/22:4(7Z,10Z,13Z,16Z))	$[M+Na]^+$	807.5778	MonomethylPhosphatidylethanolamine - formed by methylation of Phosphatidylethanolamine as part of biosynthesis of Phosphatidylcholine. Usually found at trace levels
		PE-NMe(18:0/22:5(7Z,10Z,13Z,16Z,19Z))	$[M+Na]^+$	807.5778	MonomethylPhosphatidylethanolamine - formed by methylation of Phosphatidylethanolamine as part of biosynthesis of Phosphatidylcholine. Usually found at trace levels
		PE-NMe(18:0/22:5(4Z,7Z,10Z,13Z,16Z))	$[M+Na]^+$	807.5778	MonomethylPhosphatidylethanolamine - formed by methylation of Phosphatidylethanolamine as part of biosynthesis of Phosphatidylcholine. Usually found at trace levels
		PE-NMe(22:6(4Z,7Z,10Z,13Z,16Z,19Z)/20:2(11Z,14Z))	$[M+H]^+$	829.5622	MonomethylPhosphatidylethanolamine - formed by methylation of Phosphatidylethanolamine as part of biosynthesis of Phosphatidylcholine. Usually found at trace levels
		PE-NMe(20:2(11Z,14Z)/22:6(4Z,7Z,10Z,13Z,16Z,19Z))	$[M+H]^+$	829.5622	MonomethylPhosphatidylethanolamine - formed by methylation of Phosphatidylethanolamine as part of biosynthesis of Phosphatidylcholine. Usually found at trace levels

		PE-NMe(22:5(7Z,10Z,13Z,16Z,19Z)/20:3(8Z,11Z,14Z))	[M+H]+	829.5622	MonomethylPhosphatidylethanolamine - formed by methylation of Phosphatidylethanolamine as part of biosynthesis of Phosphatidylcholine. Usually found at trace levels
		PE-NMe(22:5(7Z,10Z,13Z,16Z,19Z)/20:3(5Z,8Z,11Z))	[M+H]+	829.5622	MonomethylPhosphatidylethanolamine - formed by methylation of Phosphatidylethanolamine as part of biosynthesis of Phosphatidylcholine. Usually found at trace levels
		PE-NMe(22:5(4Z,7Z,10Z,13Z,16Z)/20:3(5Z,8Z,11Z))	[M+H]+	829.5622	MonomethylPhosphatidylethanolamine - formed by methylation of Phosphatidylethanolamine as part of biosynthesis of Phosphatidylcholine. Usually found at trace levels
		PE-NMe(22:5(4Z,7Z,10Z,13Z,16Z)/20:3(8Z,11Z,14Z))	[M+H]+	829.5622	MonomethylPhosphatidylethanolamine - formed by methylation of Phosphatidylethanolamine as part of biosynthesis of Phosphatidylcholine. Usually found at trace levels
		PE-NMe(22:4(7Z,10Z,13Z,16Z)/20:4(8Z,11Z,14Z,17Z))	[M+H]+	829.5622	MonomethylPhosphatidylethanolamine - formed by methylation of Phosphatidylethanolamine as part of biosynthesis of Phosphatidylcholine. Usually found at trace levels

		PE-NMe(22:4(7Z,10Z,13Z,16Z)/20:4(5Z,8Z,11Z,14Z))	$[M+H]^+$	829.5622	MonomethylPhosphatidylethanolamine - formed by methylation of Phosphatidylethanolamine as part of biosynthesis of Phosphatidylcholine. Usually found at trace levels
		PE-NMe(20:4(8Z,11Z,14Z,17Z)/22:4(7Z,10Z,13Z,16Z))	$[M+H]^+$	829.5622	MonomethylPhosphatidylethanolamine - formed by methylation of Phosphatidylethanolamine as part of biosynthesis of Phosphatidylcholine. Usually found at trace levels
		PE-NMe(20:4(5Z,8Z,11Z,14Z)/22:4(7Z,10Z,13Z,16Z))	$[M+H]^+$	829.5622	MonomethylPhosphatidylethanolamine - formed by methylation of Phosphatidylethanolamine as part of biosynthesis of Phosphatidylcholine. Usually found at trace levels
		PE-NMe(20:3(8Z,11Z,14Z)/22:5(7Z,10Z,13Z,16Z,19Z))	$[M+H]^+$	829.5622	MonomethylPhosphatidylethanolamine - formed by methylation of Phosphatidylethanolamine as part of biosynthesis of Phosphatidylcholine. Usually found at trace levels
		PE-NMe(20:3(8Z,11Z,14Z)/22:5(4Z,7Z,10Z,13Z,16Z))	$[M+H]^+$	829.5622	MonomethylPhosphatidylethanolamine - formed by methylation of Phosphatidylethanolamine as part of biosynthesis of Phosphatidylcholine. Usually found at trace levels

		PE-NMe(20:3(5Z,8Z,11Z)/22:5(7Z,10Z,13Z,16Z,19Z))	$[M+H]^+$	829.5622	MonomethylPhosphatidylethanolamine - formed by methylation of Phosphatidylethanolamine as part of biosynthesis of Phosphatidylcholine. Usually found at trace levels
		PE-NMe(20:3(5Z,8Z,11Z)/22:5(4Z,7Z,10Z,13Z,16Z))	$[M+H]^+$	829.5622	MonomethylPhosphatidylethanolamine - formed by methylation of Phosphatidylethanolamine as part of biosynthesis of Phosphatidylcholine. Usually found at trace levels
		PC(22:6(4Z,7Z,10Z,13Z,16Z,19Z)/18:2(9Z,12Z))	$[M+H]^+$	829.5622	Phosphatidylcholine - signalling molecule on cell membrane
		PC(22:5(7Z,10Z,13Z,16Z,19Z)/18:3(9Z,12Z,15Z))	$[M+H]^+$	829.5622	Phosphatidylcholine - signalling molecule on cell membrane
		PC(22:5(7Z,10Z,13Z,16Z,19Z)/18:3(6Z,9Z,12Z))	$[M+H]^+$	829.5622	Phosphatidylcholine - signalling molecule on cell membrane
		PC(22:5(7Z,10Z,13Z,16Z,19Z)/16:0)	$[M+Na]^+$	807.5778	Phosphatidylcholine - signalling molecule on cell membrane
		PC(22:5(4Z,7Z,10Z,13Z,16Z)/18:3(6Z,9Z,12Z))	$[M+H]^+$	829.5622	Phosphatidylcholine - signalling molecule on cell membrane
		PC(22:5(4Z,7Z,10Z,13Z,16Z)/18:3(9Z,12Z,15Z))	$[M+H]^+$	829.5622	Phosphatidylcholine - signalling molecule on cell membrane
		PC(22:5(4Z,7Z,10Z,13Z,16Z)/16:0)	$[M+Na]^+$	807.5778	Phosphatidylcholine - signalling molecule on cell membrane
		PC(22:4(7Z,10Z,13Z,16Z)/18:4(6Z,9Z,12Z,15Z))	$[M+H]^+$	829.5622	Phosphatidylcholine - signalling molecule on cell membrane
		PC(22:4(7Z,10Z,13Z,16Z)/16:1(9Z))	$[M+Na]^+$	807.5778	Phosphatidylcholine - signalling molecule on cell membrane

		PC(20:5(5Z,8Z,11Z,14Z,17Z)/20:3(5Z,8Z,11Z))	$[M+H]^+$	829.5622	Phosphatidylcholine - signalling molecule on cell membrane
		PC(20:5(5Z,8Z,11Z,14Z,17Z)/20:3(8Z,11Z,14Z))	$[M+H]^+$	829.5622	Phosphatidylcholine - signalling molecule on cell membrane
		PC(20:5(5Z,8Z,11Z,14Z,17Z)/18:0)	$[M+Na]^+$	807.5778	Phosphatidylcholine - signalling molecule on cell membrane
		PC(20:4(8Z,11Z,14Z,17Z)/20:4(8Z,11Z,14Z,17Z))	$[M+H]^+$	829.5622	Phosphatidylcholine - signalling molecule on cell membrane
		PC(20:4(8Z,11Z,14Z,17Z)/20:4(5Z,8Z,11Z,14Z))	$[M+H]^+$	829.5622	Phosphatidylcholine - signalling molecule on cell membrane
		PC(20:4(8Z,11Z,14Z,17Z)/18:1(9Z))	$[M+Na]^+$	807.5778	Phosphatidylcholine - signalling molecule on cell membrane
		PC(20:4(8Z,11Z,14Z,17Z)/18:1(11Z))	$[M+Na]^+$	807.5778	Phosphatidylcholine - signalling molecule on cell membrane
		PC(20:4(5Z,8Z,11Z,14Z)/20:4(5Z,8Z,11Z,14Z))	$[M+H]^+$	829.5622	Phosphatidylcholine - signalling molecule on cell membrane
		PC(20:4(5Z,8Z,11Z,14Z)/20:4(8Z,11Z,14Z,17Z))	$[M+H]^+$	829.5622	Phosphatidylcholine - signalling molecule on cell membrane
		PC(20:4(5Z,8Z,11Z,14Z)/18:1(9Z))	$[M+Na]^+$	807.5778	Phosphatidylcholine - signalling molecule on cell membrane
		PC(20:4(5Z,8Z,11Z,14Z)/18:1(11Z))	$[M+Na]^+$	807.5778	Phosphatidylcholine - signalling molecule on cell membrane
		PC(20:3(8Z,11Z,14Z)/20:5(5Z,8Z,11Z,14Z,17Z))	$[M+H]^+$	829.5622	Phosphatidylcholine - signalling molecule on cell membrane
		PC(20:3(8Z,11Z,14Z)/18:2(9Z,12Z))	$[M+Na]^+$	807.5778	Phosphatidylcholine - signalling molecule on cell membrane
		PC(20:3(5Z,8Z,11Z)/20:5(5Z,8Z,11Z,14Z,17Z))	$[M+H]^+$	829.5622	Phosphatidylcholine - signalling molecule on cell membrane
		PC(20:3(5Z,8Z,11Z)/18:2(9Z,12Z))	$[M+Na]^+$	807.5778	Phosphatidylcholine - signalling molecule on cell membrane

		PC(20:2(11Z,14Z)/18:3(6Z,9Z,12Z))	[M+Na]+	807.5778	Phosphatidylcholine - signalling molecule on cell membrane
		PC(20:2(11Z,14Z)/18:3(9Z,12Z,15Z))	[M+Na]+	807.5778	Phosphatidylcholine - signalling molecule on cell membrane
		PC(20:1(11Z)/18:4(6Z,9Z,12Z,15Z))	[M+Na]+	807.5778	Phosphatidylcholine - signalling molecule on cell membrane
		PC(18:4(6Z,9Z,12Z,15Z)/22:4(7Z,10Z,13Z,16Z))	[M+H]+	829.5622	Phosphatidylcholine - signalling molecule on cell membrane
		PC(18:4(6Z,9Z,12Z,15Z)/20:1(11Z))	[M+Na]+	807.5778	Phosphatidylcholine - signalling molecule on cell membrane
		PC(18:3(9Z,12Z,15Z)/22:5(7Z,10Z,13Z,16Z,19Z))	[M+H]+	829.5622	Phosphatidylcholine - signalling molecule on cell membrane
		PC(18:3(9Z,12Z,15Z)/22:5(4Z,7Z,10Z,13Z,16Z))	[M+H]+	829.5622	Phosphatidylcholine - signalling molecule on cell membrane
		PC(18:3(9Z,12Z,15Z)/20:2(11Z,14Z))	[M+Na]+	807.5778	Phosphatidylcholine - signalling molecule on cell membrane
		PC(18:3(6Z,9Z,12Z)/22:5(4Z,7Z,10Z,13Z,16Z))	[M+H]+	829.5622	Phosphatidylcholine - signalling molecule on cell membrane
		PC(18:3(6Z,9Z,12Z)/22:5(7Z,10Z,13Z,16Z,19Z))	[M+H]+	829.5622	Phosphatidylcholine - signalling molecule on cell membrane
		PC(18:3(6Z,9Z,12Z)/20:2(11Z,14Z))	[M+Na]+	807.5778	Phosphatidylcholine - signalling molecule on cell membrane
		PC(18:2(9Z,12Z)/22:6(4Z,7Z,10Z,13Z,16Z,19Z))	[M+H]+	829.5622	Phosphatidylcholine - signalling molecule on cell membrane
		PC(18:2(9Z,12Z)/20:3(8Z,11Z,14Z))	[M+Na]+	807.5778	Phosphatidylcholine - signalling molecule on cell membrane
		PC(18:2(9Z,12Z)/20:3(5Z,8Z,11Z))	[M+Na]+	807.5778	Phosphatidylcholine - signalling molecule on cell membrane
		PC(18:1(9Z)/20:4(5Z,8Z,11Z,14Z))	[M+Na]+	807.5778	Phosphatidylcholine - signalling molecule on cell membrane

		PC(18:1(9Z)/20:4(8Z,11Z,14Z,17Z))	$[M+Na]^{+}$	807.5778	Phosphatidylcholine - signalling molecule on cell membrane
		PC(18:1(11Z)/20:4(5Z,8Z,11Z,14Z))	$[M+Na]^{+}$	807.5778	Phosphatidylcholine - signalling molecule on cell membrane
		PC(18:1(11Z)/20:4(8Z,11Z,14Z,17Z))	$[M+Na]^{+}$	807.5778	Phosphatidylcholine - signalling molecule on cell membrane
		PC(18:0/20:5(5Z,8Z,11Z,14Z,17Z))	$[M+Na]^{+}$	807.5778	Phosphatidylcholine - signalling molecule on cell membrane
		PC(16:1(9Z)/22:4(7Z,10Z,13Z,16Z))	$[M+Na]^{+}$	807.5778	Phosphatidylcholine - signalling molecule on cell membrane
		PC(16:0/22:5(4Z,7Z,10Z,13Z,16Z))	$[M+Na]^{+}$	807.5778	Phosphatidylcholine - signalling molecule on cell membrane
		PC(16:0/22:5(7Z,10Z,13Z,16Z,19Z))	$[M+Na]^{+}$	807.5778	Phosphatidylcholine - signalling molecule on cell membrane
769.4224	21.08	PG(20:4(8Z,11Z,14Z,17Z)-2OH(5S,6R)/i-12:0)	$[M+Na]^{+}$	746.4370	Oxidized phosphatidylglycerol
		PG(i-12:0/20:4(8Z,11Z,14Z,17Z)-2OH(5S,6R))	$[M+Na]^{+}$	746.4370	Oxidized phosphatidylglycerol
		PG(20:4(6E,8Z,11Z,13E)-2OH(5S,15S)/i-12:0)	$[M+Na]^{+}$	746.4370	Oxidized phosphatidylglycerol
		PG(i-12:0/20:4(6E,8Z,11Z,13E)-2OH(5S,15S))	$[M+Na]^{+}$	746.4370	Oxidized phosphatidylglycerol
		PG(20:4(6Z,8E,10E,14Z)-2OH(5S,12R)/i-12:0)	$[M+Na]^{+}$	746.4370	Oxidized phosphatidylglycerol
		PG(i-12:0/20:4(6Z,8E,10E,14Z)-2OH(5S,12R))	$[M+Na]^{+}$	746.4370	Oxidized phosphatidylglycerol
188.0707	2.35	Indoleacrylic acid	$[M+H]^{+}$	187.0633	Best known as plant growth hormone, biological role in animals less known. Some evidence that it promotes interstinal epithelial barrier function and mitigates inflammatory responses. Stimulating this compound could potentially have a therapeutic effect. Related compound (indolylacryloylglucine) is excreted

					more in some myopathies (e.g., Duchenne muscoular dystrophy)
		dodec-6-enedioic acid	$[M+H]^+$	228.1362	Medium-chain fatty acid
		dodec-4-enedioic acid	$[M+H]^+$	228.1362	Medium-chain fatty acid
		dodec-5-enedioic acid	$[M+H]^+$	228.1362	Medium-chain fatty acid
		dodec-2-enedioic acid	$[M+H]^+$	228.1362	Medium-chain fatty acid
229.1433	5.30	dodec-3-enedioic acid	$[M+H]^+$	228.1362	Medium-chain fatty acid
813.6839	21.40	SM(d18:2(4E,14Z)/24:0)	$[M+H]^+$	812.6771	Sphingomyelin - in cell membranes, especially the myelin sheath on nerve cell axons. Play a role in signal transduction, accumulated in Niemann-Pick Disease (rare hereditary disease)
		SM(d18:1/24:1(15Z))	$[M+H]^+$	812.6771	Sphingomyelin - in cell membranes, especially the myelin sheath on nerve cell axons. Play a role in signal transduction, accumulated in Niemann-Pick Disease (rare hereditary disease)
377.2659	13.29	Glyceryl monolinoleate	$[M+Na]^+$	354.2770	Lineolic acids and derivatives
		11,12-Epoxyeicosatrienoic acid	$[M+Na]^+$	320.2351	Epoxyeicosatrienoic acid – Formation enhanced by induction of CYP2C8 in native coronary artery endothelial cells by beta-naphthoflavone
		8-hydroxy-5Z,9E,11Z,14Z-eicosatetraenoic acid	$[M+Na]^+$	320.2351	Hydroxyeicosatetraenoic acids
		7-HETE	$[M+Na]^+$	320.2351	Hydroxyeicosatetraenoic acids
343.2241	11.17	18-Hydroxy-5Z,8Z,11Z,14Z-eicosatetraenoic acid	$[M+Na]^+$	320.2351	Hydroxyeicosatetraenoic acids

		15R-hydroxy-5Z,8Z,11Z,13E-eicosatetraenoic acid	$[M+Na]^+$	320.2351	Hydroxyeicosatetraenoic acids
		12S-hydroxy-5E,8Z,10Z,14Z-eicosatetraenoic acid	$[M+Na]^+$	320.2351	Hydroxyeicosatetraenoic acids
		12-HETE	$[M+Na]^+$	320.2351	Eicosanoid – 5-lipoxygenase metabolite of arachidonic acid. Associated with pathogenesis of hypertension and may mediate angiotensin II and TGFbeta induced mesangial cell abnormality in diabetic nephropathy. Elevated in psoriatic lesions. Vasoconstrictor Eicosanoid. 12-HETE is a neuromodulator that is synthesized during ischemia. Its neuronal effects include attenuation of calcium influx and glutamate release as well as inhibition of AMPA receptor (AMPA-R) activation. 12-HETE is found to be associated with peroxisomal biogenesis defect and Zellweger syndrome, which are inborn errors of metabolism.
		12 Hydroxy arachidonic acid	$[M+Na]^+$	320.2351	Long chain fatty acid
		17-HETE	$[M+Na]^+$	320.2351	Cytochrome P450 metabolite of arachidonic acid – Stereospecific effects on sodium transport in the kidney
		13-HETE	$[M+Na]^+$	320.2351	One of main metabolites of 15-lipoxygenases. Inhibit cell proliferation and induce apoptosis

					in several human cancer. Arachidonic acid metabolite in rat
		10-HETE	[M+Na]+	320.2351	Hydroxyeicosatetraenoic acid – Omega-hydroxy derivatives. One of major products of NADPH-dependent arachidonic acid metabolism in rat liver microsomes
		19(S)-HETE	[M+Na]+	320.2351	Hydroxyeicosatetraenoic acids
		5-HETE	[M+Na]+	320.2351	Hydroxyeicosatetraenoic acids – Eicosanoid. Intermediate in arachidonic acid metabolism via glutathione peroxidase. Involved in pathway of leukotriene synthesis. Modulator of tubuloglomerular feedback
		9-HETE	[M+Na]+	320.2351	Hydroxyeicosatetraenoic acids
		18-Hydroxyarachidonic acid	[M+Na]+	320.2351	Substrate for CYP4F8 or Leukotriene B4 20-monooxygenase. Enzymes irreversibly hydroxylates arachidonic acid to (18R)-hydroxyarachidonate in endoplasmic reticulum. Is intermediate in Eicosanoid metabolism
		20-Hydroxyeicosatetraenoic acid	[M+Na]+	320.2351	Metabolite of arachidonic acid. Potent constrictor of renal, cerebral, mesenteric arteries. Involved with activation of protein kinase, Rho kinase, MAP kinase pathway. Increases intracellular Ca2+ via depolarization of vascular smooth

					muscle membrane secondary to blocking the large-conductance Ca2+-activated K+-channels and by direct effect on L-type Ca channels. Elevations in the production of 20-HETE mediate the myogenic response of skeletal, renal, and cerebral arteries to elevations in transmural pressure. There is an important interaction between nitric oxide (NO) and the formation of 20-HETE production. NO inhibits the formation of 20-HETE formation in renal and cerebral arteries. A fall in levels of 20-HETE contributes to the cyclic GMP-independent dilator effect of NO to activate the large-conductance Ca2+-activated K+-channels and to dilate the cerebral arteries
		11(R)-HETE	$[M+Na]^+$	320.2351	Produced from arachidonic acid via COX-1 and COX-2. Secreted by endothelial cells to potentially influence vascular tone, leukocyte function, platelet aggregation
		8-HETE	$[M+Na]^+$	320.2351	Hydroxyeicosatetraenoic acid Eicosanoid. Strong activator of peroxisome proliferator-activated receptors (PPARs) alpha and weak activator of PPAR gamma. Able to

					induce differentiation of preadipocytes
		16(R)-HETE	$[M+Na]^+$	320.2351	Metabolite of arachidonic acid, metabolized by the enzyme Cytochrome P450, family 2, subfamily C. Endogenous lipidic inhibitor of human neutrophil inhibitor of adhesion and aggregation activity. Human polymorphonuclear leukocytes (PMNs) produce 16(R)-HETE that modulates their function
		14,15-Epoxy-5,8,11-eicosatrienoic acid	$[M+Na]^+$	320.2351	Epoxyeicosatrienoic acid (EET), a metabolite of arachidonic acid
		15-HETE	$[M+Na]^+$	320.2351	Hydroxyeicosatetraenoic acids. Antiinflammatory capacities. In vitro, 15-HETE has been shown to inhibit LTB4 formation, 12-HETE formation, and specifically inhibits the neutrophil chemotactic effect of LTB4. 15-HETE inhibits LTB4-induced erythema and edema, and reduces LTB4 in the synovial fluid of carragheenan-induced experimental arthritis in dogs. 15-HETE also has some immunomodulatory effects. It inhibits the mixed lymphocyte reaction, induces generation of murine cytotoxic suppressor T cells, and it decreases interferon production by murine lymphoma

					cells. 15(S)-HETE is found to be associated with Zellweger syndrome, which is an inborn error of metabolism.
		14R,15S-EpETrE	$[M+Na]^+$	320.2351	Dominant extracellular metabolite of epoxygenase products of arachidonic acid released from human luteinised granulosa cells
		8,9-Epoxyeicosatrienoic acid	$[M+Na]^+$	320.2351	Epoxyeicosatrienoic acid Eicosanoid, a metabolite of arachidonic acid
		5,6-Epoxy-8,11,14-eicosatrienoic acid	$[M+Na]^+$	320.2351	Epoxyeicosatrienoic acid Eicosanoid, a metabolite of arachidonic acid
202.1437	3.85	(5Z,7E)-3-hydroxydeca-5,7-dienoic acid	$[M+NH_4]^+$	184.1099	Medium chain-hydroxy fatty acid
		(4E,7E)-3-hydroxydeca-4,7-dienoic acid	$[M+NH_4]^+$	184.1099	Medium chain-hydroxy fatty acid
		(6Z,8E)-3-hydroxydeca-6,8-dienoic acid	$[M+NH_4]^+$	184.1099	Medium chain-hydroxy fatty acid
		(4E,6Z)-3-hydroxydeca-4,6-dienoic acid	$[M+NH_4]^+$	184.1099	Medium chain-hydroxy fatty acid
		N-Acetylaminooctanoic acid	$[M+H]^+$	201.1365	N-acyl-alpha amino acids
		(E)-10-Oxo-8-decenoic acid	$[M+NH_4]^+$	184.1099	Medium chain fatty acid
		Capryloylglycine	$[M+H]^+$	201.1365	Type of acylglycine - normally minor metabolites of fatty acids. Excretion of certain acylglycines is increased in several inborn errors of metabolism. In certain cases, the measurement of these metabolites in body fluids can be used to diagnose disorders associated with mitochondrial fatty acid beta-oxidation, including medium-chain acyl-coenzyme A (CoA) dehydrogenase (MCAD) deficiency

					and multiple acyl-CoA dehydrogenation defect (MAD)
313.1619	4.90	1-Octen-3-yl glucoside	$[M+Na]^+$	290.1729	Fatty acyl glycosides of mono- and disaccharides
415.2818	11.04	3beta,12beta-Dihydroxy-5beta-cholanoic acid	$[M+Na]^+$	392.2927	Bile acid
		Isodeoxycholic acid	$[M+Na]^+$	392.2927	Human fecal bile acid
		7a,12b-dihydroxy-5b-Cholan-24-oic acid	$[M+Na]^+$	392.2927	Bile acid
		7b,12a-Dihydroxycholanoic acid	$[M+Na]^+$	392.2927	Bile acid
		Murocholic acid	$[M+Na]^+$	392.2927	Bile acid
		Hyodeoxycholic acid	$[M+Na]^+$	392.2927	Bile acid
		Isoursodeoxycholic acid	$[M+Na]^+$	392.2927	Bile acid
		Isohyodeoxycholic acid	$[M+Na]^+$	392.2927	Bile acid
		Deoxycholic acid	$[M+Na]^+$	392.2927	Secondary bile acid
		Allochenodeoxycholic acid	$[M+Na]^+$	392.2927	Bile acid
		Chenodeoxycholic acid	$[M+Na]^+$	392.2927	Bile acid
		Allodeoxycholic acid	$[M+Na]^+$	392.2927	Bile acid
		3b,12a-Dihydroxy-5b-cholanoic acid	$[M+Na]^+$	392.2927	Bile acid
		3a,12b-Dihydroxy-5b-cholanoic acid	$[M+Na]^+$	392.2927	Bile acid
		3a,7a-Dihydroxycholanoic acid	$[M+Na]^+$	392.2927	chenodeoxycholic or bile acid present in human blood serum and in the biliary excretion of patients with choledochostomy drainage
		3b,7a-Dihydroxy-5b-cholanoic acid	$[M+Na]^+$	392.2927	Bile acid
		3b,12a-Dihydroxy-5a-cholanoic acid	$[M+Na]^+$	392.2927	Bile acid
		Ursodeoxycholic acid	$[M+Na]^+$	392.2927	Dihydroxy bile acids, alcohols and derivatives
424.3419	9.86	O-Linoleoylcarnitine	$[M+H]^+$	423.3349	Acylcarnitine
		MG(22:4(7Z,10Z,13Z,16Z)/0:0/0:0)	$[M+NH_4]^+$	406.3083	monoacylglycerol - formed biochemically via release of a fatty acid from diacylglycerol by

					diacylglycerol lipase or hormone sensitive lipase, also added to food so potential exposome
		MG(0:0/22:4(7Z,10Z,13Z,16Z)/0:0)	$[M+NH_4]^+$	406.3083	monoacylglycerol - formed biochemically via release of a fatty acid from diacylglycerol by diacylglycerol lipase or hormone sensitive lipase, also added to food so potential exposome
		Linoleyl carnitine	$[M+H]^+$	423.3349	Long-chain acyl fatty acid derivative of carnitine. Long-chain acyl fatty acid derivatives accumulate in the cytosol and serum of patients suffering of mitochondrial carnitine palmitoyltransferase II deficiency. Linoleyl carnitine is found to be associated with glutaric aciduria II, which is an inborn error of metabolism.
		DG(20:3(8Z,11Z,14Z)-2OH(5,6)/0:0/i-16:0)	$[M+NH_4]^+$	650.5122	Diacylglycerols- glycerolipids containing a common glycerol backbone with at least one fatty acyl group esterified. Involved in the phospholipid metabolic pathway
		DG(i-16:0/0:0/20:3(8Z,11Z,14Z)-2OH(5,6))	$[M+NH_4]^+$	650.5122	Diacylglycerols- glycerolipids containing a common glycerol backbone with at least one fatty acyl group esterified. Involved in the phospholipid metabolic pathway
668.5443	8.65	DG(20:3(8Z,11Z,14Z)-2OH(5,6)/i-16:0/0:0)	$[M+NH_4]^+$	650.5122	Diacylglycerols- glycerolipids containing a common glycerol

					backbone with at least one fatty acyl group esterified. Involved in the phospholipid metabolic pathway
		DG(i-16:0/20:3(8Z,11Z,14Z)-2OH(5,6)/0:0)	$[M+NH_4]^+$	650.5122	Diacylglycerols- glycerolipids containing a common glycerol backbone with at least one fatty acyl group esterified. Involved in the phospholipid metabolic pathway
		DG(20:3(8Z,11Z,14Z)-2OH(5,6)/0:0/16:0)	$[M+NH_4]^+$	650.5122	Diacylglycerols- glycerolipids containing a common glycerol backbone with at least one fatty acyl group esterified. Involved in the phospholipid metabolic pathway
		DG(16:0/0:0/20:3(8Z,11Z,14Z)-2OH(5,6))	$[M+NH_4]^+$	650.5122	Diacylglycerols- glycerolipids containing a common glycerol backbone with at least one fatty acyl group esterified. Involved in the phospholipid metabolic pathway
		DG(20:3(8Z,11Z,14Z)-2OH(5,6)/16:0/0:0)	$[M+NH_4]^+$	650.5122	Diacylglycerols- glycerolipids containing a common glycerol backbone with at least one fatty acyl group esterified. Involved in the phospholipid metabolic pathway
		DG(16:0/20:3(8Z,11Z,14Z)-2OH(5,6)/0:0)	$[M+NH_4]^+$	650.5122	Diacylglycerols- glycerolipids containing a common glycerol backbone with at least one fatty acyl group esterified. Involved in the phospholipid metabolic pathway
269.1357	5.45	5'-Deoxyadenosine	$[M+NH_4]^+$	251.1018	Oxidized nucleoside
		3-Hydroxydodecanedioic acid	$[M+Na]^+$	246.1467	Medium-chain hydroxy acids

		Deoxyadenosine	$[M+NH_4]^+$	251.1018	Derivative of nucleoside adenosine - Chronically high levels of deoxyadenosine are associated with adenosine deaminase (ADA) deficiency, an inborn error of metabolism. The main symptoms of ADA deficiency are pneumonia, chronic diarrhea, and widespread skin rashes
320.2429	6.63	xi-8-Hydroxyhexadecanedioic acid	$[M+NH_4]^+$	302.2093	Long-chain fatty acids
		xi-7-Hydroxyhexadecanedioic acid	$[M+NH_4]^+$	302.2093	Long-chain fatty acids

Table S8: Complete List of Tentatively Identified Exogenous Metabolites in Low vs. Moderate Models

m/z	RT	Tentative ID	Adduct	Monoisotopic Mass	Biological Significance
415.2536	3.97	Methyl N-({(2s,3s)-3-[(Propylamino)carbonyl]oxiran-2-Yl}carbonyl)-L-Isoleucyl-L-Prolinate	$[M+NH_4]^+$	397.2213	Peptides
116.0821	0.56	1-Nitrosopiperazine	$[M+H]^+$	115.0746	Piperazines
		4-Hydroxymethylpyrazole	$[M+NH_4]^+$	98.0480	Pyrazoles – Used alone or in combination with hemodialysis. Used as antidote for methanol/ethylene glycol poisoning
239.9939	0.67	2-Aminophenol N-formate sulfate	$[M+Na]^+$	217.0045	Bacterial metabolite
391.1877	9.86	Sepimostat	$[M+NH_4]^+$	373.1539	Naphthalenes
		N-(4-(4-Methyl-1-piperazinyl)phenyl)-9-acridinamine	$[M+Na]^+$	368.2001	Acridines
		Clebopride	$[M+NH_4]^+$	373.1557	N-benzylpiperidines
524.3710	14.21	Anadur	$[M+NH_4]^+$	506.3396	Steroid esters
		2-LysoPhosphatidylcholine	$[M+H]^+$	523.3638	1-acyl-sn-glycero-3-phosphocholines
251.1430	9.04	Non-sulfonylurea	$[M+H]^+$	250.1351	Sulfonylureas
263.0887	6.70	Isopropyl 3-(3,4-dihydroxyphenyl)-2-hydroxypropanoate	$[M+Na]^+$	240.0998	Catechols
		Ethyl 3,4,5-trimethoxybenzoate	$[M+Na]^+$	240.0998	Gallic acid and derivatives
		L-beta-aspartyl-L-glutamic acid	$[M+H]^+$	262.0801	Hybrid peptides – Found in anatidaes, chicken, pigs
		3-(3,4,5-Trimethoxyphenyl)propanoic acid	$[M+Na]^+$	240.0998	Phenylpropanoic acids

188.0707	2.35	6-Chloro-N-(1-methylethyl)-1,3,5-triazine-2,4-diamine	$[M+H]^+$	187.0625	1,3,5-triazine-2,4-diamines – Primary metabolites
549.1857	16.38	Neoacrimarine F	$[M+NH_4]^+$	531.1529	Triacylglycerols
		Neoacrimarine I	$[M+NH_4]^+$	531.1529	Acridones – Found in citrus
646.2575	18.76	Acarbosa	$[M+H]^+$	645.2480	Aminocyclitol glycosides
		Alpha-Acarbose	$[M+H]^+$	645.2480	Aminocyclitol glycosides
		Acarbose	$[M+H]^+$	645.2480	Aminocyclitol glycosides – Drug that inhibits alpha glucosidase that retards the digestion and absorption of carbohydrates in the small intestine, hence reducing the increase in blood-glucose concentration after a carbohydrate load.
229.1433	5.30	Butanedioic acid, octenyl-	$[M+H]^+$	228.1362	Fatty acid esters
		5-Heptyltetrahydro-2-oxo-3-furancarboxylic acid	$[M+H]^+$	228.1362	Gamma butyrolactones
		Traumatic acid	$[M+H]^+$	228.1362	Monounsaturated dicarboxylic acid – Fount in plants. Potent wound healing agent in plants
377.2659	13.29	MG(18:2(9Z,12Z)/0:0/0:0)	$[M+Na]^+$	354.2770	Monoacylglycerol - formed biochemically via release of a fatty acid from diacylglycerol by diacylglycerol lipase or hormone sensitive lipase, also found in food so potential exposome
		MG(0:0/18:2(9Z,12Z)/0:0)	$[M+Na]^+$	354.2770	Monoacylglycerol - formed biochemically via release of a fatty acid from diacylglycerol by

					diacylglycerol lipase or hormone sensitive lipase, also found in food so potential exposome
		4,7,13,16,21,24-Hexaoxa-1,10-diazabicyclo[8.8.8]hexacosane	$[M+H]^+$	376.2573	Trialkylamines
		2-Linoleoyl Glycerol	$[M+Na]^+$	354.2770	Lineolic acids and derivatives
		Rioprostil	$[M+Na]^+$	354.2770	Fatty alcohols
		Lavoltidine	$[M+NH_4]^+$	359.2321	N-benzylpiperidines
		Gepirone	$[M+NH_4]^+$	359.2321	N-arylpiperazines – Antidepressant and anxiolytic drug of azapirone group. gepirone metabolizes into 1-(2-pyrimidinyl)piperazine, which is known to act as a potent antagonist of the α2-adrenergic receptor
343.2241	11.17	(3R-(3alpha,4alpha(2R*,3R*),5beta,6beta))-5-Methoxy-4-(2-methyl-3-(3-methyl-2-butenyl)oxiranyl)-1-oxaspiro(2.5)octan-6-ol carbamate	$[M+NH_4]^+$	325.1889	Carbamate esters
		18-Hydroxyeicosatetraenoic acid	$[M+Na]^+$	320.2351	Hydroxyeicosatetraenoic acids
		(5E,8E,11E,14E)-19-Hydroxyicosa-5,8,11,14-tetraenoic acid	$[M+Na]^+$	320.2351	Hydroxyeicosatetraenoic acids
		(5Z,8Z,11Z,14Z)-3-hydroxyicosa-5,8,11,14-tetraenoic acid	$[M+Na]^+$	320.2351	Hydroxyeicosatetraenoic acids
		12,15-Epoxy-13,14-dimethyloctadeca-12,14,16-trienoic acid	$[M+Na]^+$	320.2351	Unsaturated furan fatty acids – Found in low concentration in food lipids. Found in fish. Excellent antioxidants and radical scavengers. Play an important role in preventing lipid peroxidation

					and protecting polyunsaturated fatty acids
		12,15-Epoxy-13,14-dimethyloctadeca-10,12,14-trienoic acid	[M+Na]+	320.2351	Unsaturated furan fatty acids – Found in low concentration in food lipids. Found in fish. Excellent antioxidants and radical scavengers. Play an important role in preventing lipid peroxidation and protecting polyunsaturated fatty acids
		4-[3-(Tetradeca-2,5,8-trien-1-yl)oxiran-2-yl]butanoic acid	[M+Na]+	320.2351	Epoxy fatty acids
		(14R,15S)-14,15-Epoxy-5,8,11-icosatrienoic acid	[M+Na]+	320.2351	Long-chain fatty acids
		(Z)-7-((2S,3R)-3-((2Z,5Z)-Undeca-2,5-dienyl)oxiran-2-yl)hept-5-enoic acid	[M+Na]+	320.2351	Long-chain fatty acids
		Crispane	[M+Na]+	320.2351	Found in herbs and spices
		12(S)-Hydroxy-5,8,10,14-eicosatetraenoic acid	[M+Na]+	320.2351	Hydroxyeicosatetraenoic acids
		2-Hydroxy-6-tridecylbenzoic acid	[M+Na]+	320.2351	Salicylic acids – Found in fats, oils, nuts
		(ent-2alpha,3beta,15beta)-16-Kaurene-2,3,15-triol	[M+Na]+	320.2351	Kaurane diterpenoids
		(ent-2alpha,3beta,15beta,16beta)-15,16-Epoxy-2,3-kauranediol	[M+Na]+	320.2351	Kaurane diterpenoids
		Ucriol	[M+Na]+	320.2351	Kaurane diterpenoids
		8alpha,13R-Epoxy-14-labden-19-oic acid	[M+Na]+	320.2351	Triterpenoids
		Annoglabasin E	[M+Na]+	320.2351	Kaurane diterpenoids
		15-Hydroxy-5,8,11,13-eicosatetraenoic acid	[M+Na]+	320.2351	Hydroxyeicosatetraenoic acids
		11-Hydroxy-5Z,8Z,11E,14Z-eicosatetraenoic acid	[M+Na]+	320.2351	Hydroxyeicosatetraenoic acids
		Aloxistatin	[M+H]+	342.2155	Leucine and derivatives

		Isocupressic acid	$[M+Na]^+$	320.2351	Diterpenoids
		Mutilin	$[M+Na]^+$	320.2351	Mutilin derivatives
		1-Hydroxy-2,2,5,5-tetramethylpyrrolidine-3-carboxylic acid methyl ester	$[M+H]^+$	201.1365	Pyrrolidine carboxylic acids
		1-Acetylcyclohexyl acetate	$[M+NH_4]^+$	184.1099	Alpha-acyloxy ketones
		(S)-Oleuropeic acid	$[M+NH_4]^+$	184.1099	Menthane monoterpenoids
		alpha-Campholonic acid	$[M+NH_4]^+$	184.1099	Iridoids and derivatives – Found in sage
		(4S,8R)-8,9-Dihydroxy-p-menth-1(6)-en-2-one	$[M+NH_4]^+$	184.1099	Menthane monoterpenoids
		1,3-Bis(hydroxymethyl)-3-methylbicyclo[2.2.1]heptan-2-one	$[M+NH_4]^+$	184.1099	Bicyclic monoterpenoids
		(1'R)-Nepetalic acid	$[M+NH_4]^+$	184.1099	Monocyclic monoterpenoids
		N-(5-Methyl-3-oxohexyl)alanine	$[M+H]^+$	201.1365	Alanine and derivatives
202.1437	3.85	Valproylglycine	$[M+H]^+$	201.1365	N-acyl-alpha amino acids
		3,5-Diisopropylsalicylic acid	$[M+NH_4]^+$	222.1256	Salicylic acids
		3-Hexylsalicylic acid	$[M+NH_4]^+$	222.1256	Salicylic acids
		(+)-Dehydrovomifoliol	$[M+NH_4]^+$	222.1256	Sesquiterpenoids – Found in rice
		Hexyl salicylic acid	$[M+NH_4]^+$	222.1256	O-hydroxybenzoic acid esters
240.1592	5.52	3-Hydroxyibuprofen	$[M+NH_4]^+$	222.1256	Phenylpropanoic acids – Metabolite of ibuprofen. 3-hydroxyibuprofen can be converted into carboxy-

					ibuprofen; which is mediated by the enzyme cytochrome P450 2C9
		2-Hydroxyibuprofen	$[M+NH_4]^+$	222.1256	Phenylpropanoic acids – Metabolite of ibuprofen
		1-Hydroxyibuprofen	$[M+NH_4]^+$	222.1256	Metabolite of ibuprofen
		Moprolol	$[M+H]^+$	239.1521	Beta-adrenergic antagonist or beta blocker – Treats hypertensio, high blood pressure, angina pectoris, arrhythmias, anxiety, glaucoma
		Methyl 3-epi-4,5-didehydrojasmonate	$[M+NH_4]^+$	222.1256	Methyl esters
		Annuionone B	$[M+NH_4]^+$	222.1256	Oxepanes – found in fats, oils, sunflower
		Isoamyl p-anisate	$[M+NH_4]^+$	222.1256	P-methoxybenzoic acids and derivatives
		Dehydrovomifoliol	$[M+NH_4]^+$	222.1256	Sesquiterpenoids
		Petasinine	$[M+H]^+$	239.1521	Pyrrolizidines
		3alpha-Tigloyloxytropane N-oxide	$[M+H]^+$	239.1521	Tropane alkaloids – Found in fruits
		Salbutamol	$[M+H]^+$	239.1521	Short-acting, selective beta2-adrenergic receptor agonist. Salbutamol is generally used for acute episodes of bronchospasm caused by bronchial asthma, chronic bronchitis and other chronic bronchopulmonary disorders such as chronic obstructive pulmonary disorder (COPD). It is also used

					prophylactically for exercise-induced asthma
		Isoetharine	[M+H]+	239.1521	Catechols - drug which is used for the treatment of asthma, wheezing, and chronic asthmatic bronchitis
		N2-Ethyl-2'-deoxyguanosine	$[M+NH_4]^+$	295.1281	Purine 2'-deoxyribonucleosides
313.1619	4.90	N(6),O(2)-Dimethyladenosine	$[M+NH_4]^+$	295.1281	Purine nucleosides
185.0961	6.78	Benzyl phenyl ether	[M+H]+	184.0888	Phenol ethers
		Methyl-[12]-gingerol	[M+Na]+	392.2927	Dimethoxybenzenes – Found in ginger
		4-[(5R,8S,10R,13R,17R)-3,6-Dihydroxy-10,13-dimethyl-2,3,4,5,6,7,8,9,11,12,14,15,16,17-tetradecahydro-1H-cyclopenta[a]phenanthren-17-yl]pentanoic acid	[M+Na]+	392.2927	Dihydroxy bile acids, alcohols and derivatives
		3,7-Dihydroxycholan-24-oic acid	[M+Na]+	392.2927	Dihydroxy bile acids, alcohols and derivatives
415.2818	11.04	3alpha,12beta-Dihydroxy-5alpha-cholan-24-oic Acid	[M+Na]+	392.2927	Dihydroxy bile acids, alcohols and derivatives
424.3419	9.86	[14]-Gingerol	$[M+NH_4]^+$	406.3083	Gingerols – Found in ginger
		9H-Purine-9-butanoic acid, 6-amino-alpha-hydroxy-, methyl ester	$[M+NH_4]^+$	251.1018	6-aminopurines
		2',3'-Dideoxyguanosine	$[M+NH_4]^+$	251.1018	Purine 2',3'-dideoxyribonucleosides
		Cordycepin	$[M+NH_4]^+$	251.1018	Purine 3'-deoxyribonucleosides
269.1357	5.45	Dibutyl malate	[M+Na]+	246.1467	Beta hydroxy acids and derivatives

320.2429	6.63	Gemcabene	$[M+NH_4]^+$	302.2093	Medium-chain fatty acids
263.0814	7.48	Aminonitropyrene	$[M+H]^+$	262.0742	Pyrenes
		Indigo	$[M+H]^+$	262.0742	Oxindole dimer – Detected in human urine and tissue. Mammals produce minute amount but are mostly synthetic. Dye
		Indirubin	$[M+H]^+$	262.0742	Oxindole dimer - – Detected in human urine and tissue. Mammals produce minute amount but are mostly extracted from plants. Indirubin has shown anti-inflammatory and anti-angiogenesis properties in vitro. It has also been studied for potential use in the treatment of ulcerative colitis
		Lamivudine sulfoxide	$[M+NH_4]^+$	245.0470	Nucleoside and nucleotide analogues. In humans, lamivudine sulfoxide is involved in lamivudine metabolism pathway. Can be biosynthesized, but also a synthetic antiprotozoal agent

Table S9: Tentatively Identified Endogenous Metabolites for Low vs. Moderate Disease Activity in Negative Mode

m/z	RT	Tentative ID	Adduct	Monoisotopic Mass	Biological Significance
		7-Hydroxyindole sulfate	$[M-H]^-$	213.0096	arylsulfates
		6-Hydroxyindole sulfate	$[M-H]^-$	213.0096	arylsulfates
212.0026	3.97	Indoxyl sulfate	$[M-H]^-$	213.0096	arylsulfates. Metabolite of tryptophan. Derived through consumption, digestion, microbial processing of protein rich foods (Derived from host and bacteria). Can bind serum albumin. Agonist for arylhydrocarbon receptor. High concentrations in blood associated with chronic kidney disease, and cardiovascular disease. Involved with oxidative stress, impair osteoblast and bone turnover. Is a myotoxin, neurotxoin, etc.
729.1788	11.02	Neocuscutoside C	$[M+Cl]^-$	694.2109	lignan glycosdes
		glyco-beta-muricholic acid	$[M-H]^-$	465.3090	extremely weak basic
		N-Palmitoyl tyrosine	$[M+FA-H]^-$	419.3036	N-acylamides. Is an amino acid conjugate classified as long chain N-acylamide. Could be associated with signaling, cell migration, inflammation, etc.
		Glycohyocholic acid	$[M-H]^-$	465.3090	Bile acid. Involved in excretion, absorption, transport of fats and sterols. But could also disrupt membranes. Plethora of mechanisms to limit accumulation in blood and tissues
464.3020	7.39	Glycocholic acid	$[M-H]^-$	465.3090	acyl glycine and bile acid-glycine conjugate. Secondary bile acid produced by microbiome. Involved in fat/lipid absorption, transport, excretion. Could be associated with in-born

					error of metabolism - alpha-1-antitrypsin deficiency
		Stearidonyl carnitine	$[M+FA-H]^-$	419.3036	acylcarnitine. Associated with energy production and intermediary metabolism.
		3a,7b,12a-Trihydroxyoxocholanyl-Glycine	$[M-H]^-$	465.3090	Acyl glycine and bile acid-glycine conjugate. Secondary bile acid produced by microbiome. Specific ketone bile acid found in urine of infants during neonatal period
		PC(18:1(9Z)-O(12,13)/P-16:0)	$[M+FA-H]^-$	757.5622	Oxidized phosphatidylcholine. Role as signaling molecules. Biosynthesis by COX, LOX, CYP, or through uncontrolled oxidation via free radicals. Key components of lipid bilayer, involved in metabolism, signaling
		PC(P-16:0/18:1(9Z)-O(12,13))	$[M+FA-H]^-$	757.5622	Oxidized phosphatidylcholine. Role as signaling molecules. Biosynthesis by COX, LOX, CYP, or through uncontrolled oxidation via free radicals. Key components of lipid bilayer, involved in metabolism, signaling
		PC(18:1(12Z)-O(9S,10R)/P-16:0)	$[M+FA-H]^-$	757.5622	Oxidized phosphatidylcholine. Role as signaling molecules. Biosynthesis by COX, LOX, CYP, or through uncontrolled oxidation via free radicals. Key components of lipid bilayer, involved in metabolism, signaling
		PC(P-16:0/18:1(12Z)-O(9S,10R))	$[M+FA-H]^-$	757.5622	Oxidized phosphatidylcholine. Role as signaling molecules. Biosynthesis by COX, LOX, CYP, or through uncontrolled oxidation via free radicals. Key components of lipid bilayer, involved in metabolism, signaling
802.5614	13.14	PC(20:2(11Z,14Z)/14:0)	$[M+FA-H]^-$	757.5622	Glycerophospholipid. Derived from oils and liver. Key components of lipid bilayer, metabolism, signaling

		PC(20:1(11Z)/14:1(9Z))	$[M+FA-H]^-$	757.5622	Glycerophospholipid. Derived from oils and liver. Key components of lipid bilayer, metabolism, signaling
		PC(18:2(9Z,12Z)/16:0)	$[M+FA-H]^-$	757.5622	Glycerophospholipid. Derived from oils and liver. Key components of lipid bilayer, metabolism, signaling
		PC(18:1(9Z)/16:1(9Z))	$[M+FA-H]^-$	757.5622	Glycerophospholipid. Derived from oils and liver. Key components of lipid bilayer, metabolism, signaling
		PC(18:1(11Z)/16:1(9Z))	$[M+FA-H]^-$	757.5622	Glycerophospholipid. Derived from oils and liver. Key components of lipid bilayer, metabolism, signaling
		PC(16:1(9Z)/18:1(11Z))	$[M+FA-H]^-$	757.5622	Glycerophospholipid. Derived from oils and liver. Key components of lipid bilayer, metabolism, signaling
		PC(16:1(9Z)/18:1(9Z))	$[M+FA-H]^-$	757.5622	Glycerophospholipid. Derived from oils and liver. Key components of lipid bilayer, metabolism, signaling
		PC(16:0/18:2(9Z,12Z))	$[M+FA-H]^-$	757.5622	Glycerophospholipid. Derived from oils and liver. Key components of lipid bilayer, metabolism, signaling
		PC(14:1(9Z)/20:1(11Z))	$[M+FA-H]^-$	757.5622	Glycerophospholipid. Derived from oils and liver. Key components of lipid bilayer, metabolism, signaling
		PC(14:0/20:2(11Z,14Z))	$[M+FA-H]^-$	757.5622	Glycerophospholipid. Derived from oils and liver. Key components of lipid bilayer, metabolism, signaling
		PE(PGF1alpha/P-18:0)	$[M-H]^-$	803.5676	Oxidized phosphatidylethanolamine. Role as signalling molecules. Biosynthesis by LOX, COX, CYP or by uncontrolled oxidation via

					free radicals. Key components of lipid bilayer, involved in metabolism and signaling
		PE(P-18:0/PGF1alpha)	[M-H]-	803.5676	Oxidized phosphatidylethanolamine. Role as signalling molecules. Biosynthesis by LOX, COX, CYP or by uncontrolled oxidation via free radicals. Key components of lipid bilayer, involved in metabolism and signaling
		PE(18:1(12Z)-2OH(9,10)/20:1(11Z))	[M-H]-	803.5676	Oxidized phosphatidylethanolamine. Role as signalling molecules. Biosynthesis by LOX, COX, CYP or by uncontrolled oxidation via free radicals. Key components of lipid bilayer, involved in metabolism and signaling
		PE(20:1(11Z)/18:1(12Z)-2OH(9,10))	[M-H]-	803.5676	Oxidized phosphatidylethanolamine. Role as signalling molecules. Biosynthesis by LOX, COX, CYP or by uncontrolled oxidation via free radicals. Key components of lipid bilayer, involved in metabolism and signaling
		PE(22:2(13Z,16Z)/15:0)	[M+FA-H]-	757.5622	Phosphatidylethanolamine. Derived from animal fats, dairy. Key component of lipid bilayer, metabolism, signaling
		PE(15:0/22:2(13Z,16Z))	[M+FA-H]-	757.5622	Phosphatidylethanolamine. Derived from animal fats, dairy. Key component of lipid bilayer, metabolism, signaling
		PE-NMe2(20:2(11Z,14Z)/15:0)	[M+FA-H]-	757.5622	Methylphosphatidylethanolamine. Formed by sequential methylation for biosynthesis of phosphatidylcholine.
		PE-NMe2(15:0/20:2(11Z,14Z))	[M+FA-H]-	757.5622	Methylphosphatidylethanolamine. Formed by sequential methylation for biosynthesis of phosphatidylcholine.

		PE-NMe(20:2(11Z,14Z)/16:0)	[M+FA-H]-	757.5622	Methylphosphatidylethanolamine. Formed by sequential methylation for biosynthesis of phosphatidylcholine.
		PE-NMe(16:0/20:2(11Z,14Z))	[M+FA-H]-	757.5622	Methylphosphatidylethanolamine. Formed by sequential methylation for biosynthesis of phosphatidylcholine.
		PE-NMe(22:2(13Z,16Z)/14:0)	[M+FA-H]-	757.5622	Methylphosphatidylethanolamine. Formed by sequential methylation for biosynthesis of phosphatidylcholine.
		PE-NMe(22:1(13Z)/14:1(9Z))	[M+FA-H]-	757.5622	Methylphosphatidylethanolamine. Formed by sequential methylation for biosynthesis of phosphatidylcholine.
		PE-NMe(20:1(11Z)/16:1(9Z))	[M+FA-H]-	757.5622	Methylphosphatidylethanolamine. Formed by sequential methylation for biosynthesis of phosphatidylcholine.
		PE-NMe(18:2(9Z,12Z)/18:0)	[M+FA-H]-	757.5622	Methylphosphatidylethanolamine. Formed by sequential methylation for biosynthesis of phosphatidylcholine.
		PE-NMe(18:0/18:2(9Z,12Z))	[M+FA-H]-	757.5622	Methylphosphatidylethanolamine. Formed by sequential methylation for biosynthesis of phosphatidylcholine.
		PE-NMe(14:1(9Z)/22:1(13Z))	[M+FA-H]-	757.5622	Methylphosphatidylethanolamine. Formed by sequential methylation for biosynthesis of phosphatidylcholine.
		PE-NMe(14:0/22:2(13Z,16Z))	[M+FA-H]-	757.5622	Methylphosphatidylethanolamine. Formed by sequential methylation for biosynthesis of phosphatidylcholine.
		PE-NMe(18:1(9Z)/18:1(11Z))	[M+FA-H]-	757.5622	Methylphosphatidylethanolamine. Formed by sequential methylation for biosynthesis of phosphatidylcholine.

		PE-NMe(18:1(11Z)/18:1(9Z))	[M+FA-H]-	757.5622	Methylphosphatidylethanolamine. Formed by sequential methylation for biosynthesis of phosphatidylcholine.
		PE-NMe(18:1(11Z)/18:1(11Z))	[M+FA-H]-	757.5622	Methylphosphatidylethanolamine. Formed by sequential methylation for biosynthesis of phosphatidylcholine.
		PE-NMe(16:1(9Z)/20:1(11Z))	[M+FA-H]-	757.5622	Methylphosphatidylethanolamine. Formed by sequential methylation for biosynthesis of phosphatidylcholine.
		PE-NMe(18:1(9Z)/18:1(9Z))	[M+FA-H]-	757.5622	Methylphosphatidylethanolamine. Formed by sequential methylation for biosynthesis of phosphatidylcholine.
		PS(22:1(13Z)/15:0)	[M-H]-	803.5676	Phosphatidylserine. Can act as calcium chelator. anionic.
		PS(15:0/22:1(13Z))	[M-H]-	803.5676	Phosphatidylserine. Can act as calcium chelator. anionic.
448.3073	8.18	N-Palmitoyl phenylalanine	[M+FA-H]-	403.3086	N-acylamides. Amino acid conjugate, long chain N-acylamide. Could be associated with signaling, cell migration, inflammation, etc.
		Chenodeoxycholylglycine	[M-H]-	449.3141	Glycinated bile acids and derivatives.
		Glycohyodeoxycholic acid	[M-H]-	449.3141	Glycinated bile acids and derivatives.
		Deoxycholylglycine	[M-H]-	449.3141	Glycinated bile acids and derivatives.
		Chenodeoxyglycocholic acid	[M-H]-	449.3141	Glycine conjugated bile acids. Modulate bile flow and lipid secretion (Important for absorption of fats and vitamins. Facilitate excretion, absorption, transport of fats/steroids.
		Glycoursodeoxycholic acid	[M-H]-	449.3141	Acyl glycine and bile acid-glycine conjugate. Secondary bile acid produced by microbiome.

					Facilitates transport/absorption/excretion of fats/sterols.
		Deoxycholic acid glycine conjugate	$[M-H]^-$	449.3141	Bile salt formed in liver. Secondary bile acid produced by microbiome. Facilitates transport/excretion/absorption of fats/sterols
		Chenodeoxycholic acid glycine conjugate	$[M-H]^-$	449.3141	Acyl glycine and bile acid-glycine conjugate. Secondary bile acid produced by microbiome. Facilitates absorption of fats and is itself absorbed
203.0830	3.58	Nirvanol	$[M-H]^-$	204.0899	Phenylhydantoins. Primary metabolite involved in growth, development, reproduction
		3-Hydroxymethylantipyrine	$[M-H]^-$	204.0899	Phenylpyrazoles.
		D-Tryptophan	$[M-H]^-$	204.0899	Indolyl carboxylic acids and derivatives. Found in all living organisms. Secondary metabolite (Signaling/defense/incomplete metabolism of other secondary metabolites
		L-Tryptophan	$[M-H]^-$	204.0899	Alpha amino acid. Proteinogenic, found in all living organisms. Essential organisms.

Table S10: Tentatively Identified Exogenous Metabolites for Low vs. Moderate Disease Activity in Negative Mode

m/z	RT	Tentative ID	Adduct	Monoisotopic Mass	Biological Significance
212.00258	3.97	4H-Thieno[3,2-b]pyrrole-5-carboxylic acid	$[M+FA-H]^-$	167.0041	Thienopyrroles
729.17876	11.02	Sucrose 1',4'-(4,4'-dihydroxy-3,3'-dimethoxy-b-truxinate)	$[M+Cl]^-$	694.2109	Stilbene - Found in cereals, oats
		1,2-Diferuloylgentiobiose	$[M+Cl]^-$	694.2109	Hydroxycinnamic acid glycosides – Found in broccoli
		Neocuscutoside C	$[M+Cl]^-$	694.2109	Lignan glycosides
		Sesaminol glucosyl-(1->2)-glucoside	$[M+Cl]^-$	694.2109	Lignan glycosides – Found in cereal
343.1707	8.15	Tamoxifen-ol	$[M-H]^-$	344.1776	Stilbene
667.14192	11.98	Etoposide phosphate	$[M-H]^-$	668.1506	Xanthines
		Calcein	$[M+FA-H]^-$	622.1435	Pentacarboxylic acids – Fluorescent dye
388.15593	8.00	Epiroprim	$[M+Cl]^-$	353.1852	Phenylpyrroles
203.08299	3.58	2,3-Diaminonaphthalene	$[M+FA-H]^-$	158.0844	Naphthalene
		1,2-Diaminonaphthalene	$[M+FA-H]^-$	158.0844	Naphthalene
		1,5-Naphthalenediamine	$[M+FA-H]^-$	158.0844	Naphthalene
		Idazoxan	$[M-H]^-$	204.0899	Benzo-1,4-dioxanes
		Nicotyrine	$[M+FA-H]^-$	158.0844	Pyridines
		S-nirvanol	$[M-H]^-$	204.0899	Metabolite of mephenytoin, an anticonvulsant
		4-Hydroxyantipyrine	$[M-H]^-$	204.0899	Metabolite of antipyrine, an analgesic and antipyretic given via mouth or ear drops

		Ethotoin	$[M-H]^-$	204.0899	Hydantoin derivative and anticonvulsant
		1-Benzylimidazole	$[M+FA-H]^-$	158.0844	n-substituted imidazoles

Table S11: Peaks of overlapping features across models

Feature Peak	m/z
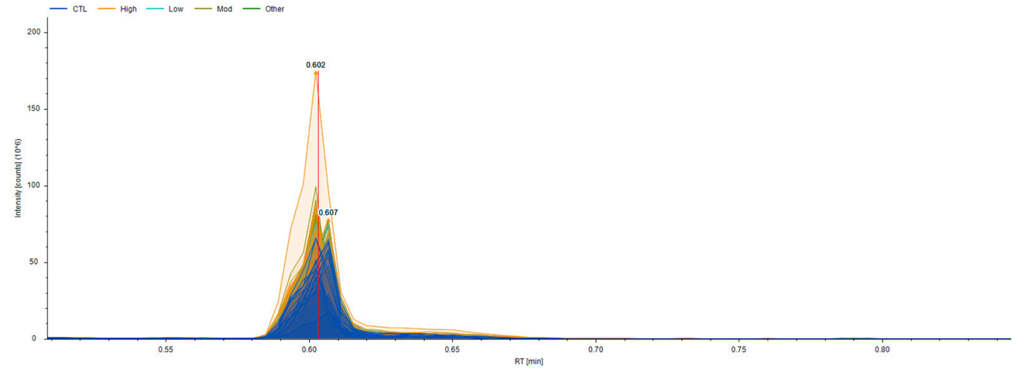	116.0708
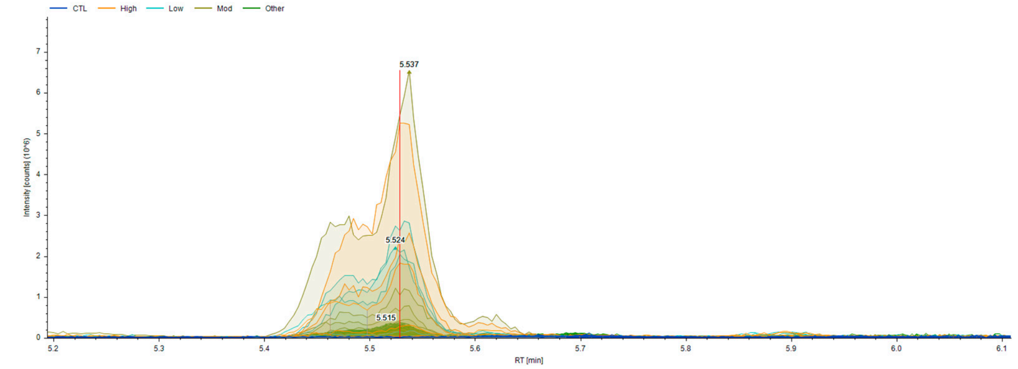	159.1168
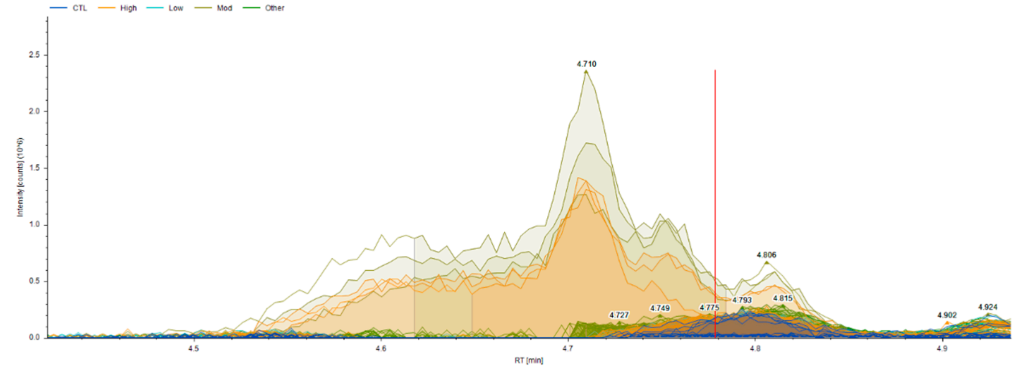	215.1279

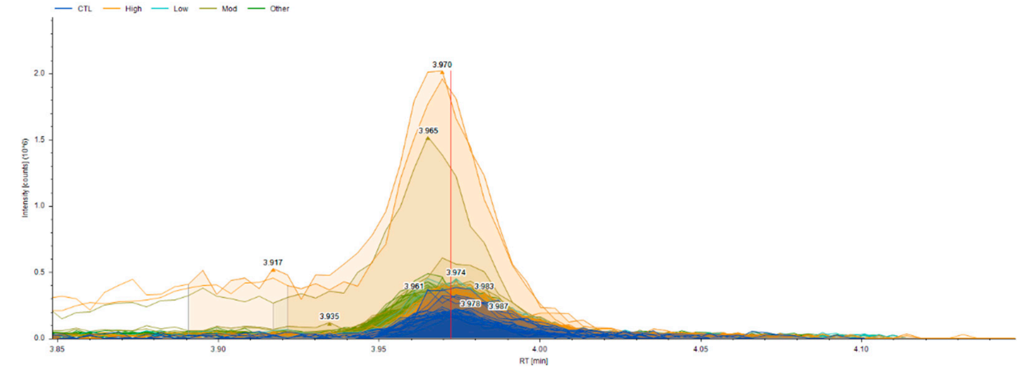	415.2536
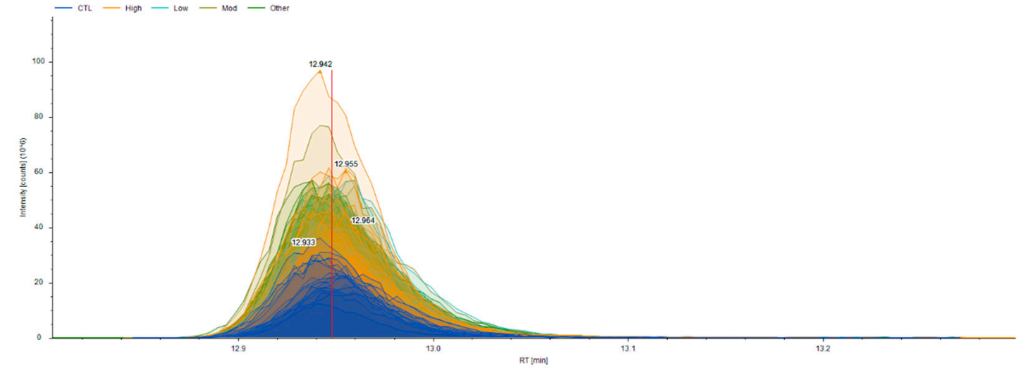	496.3397
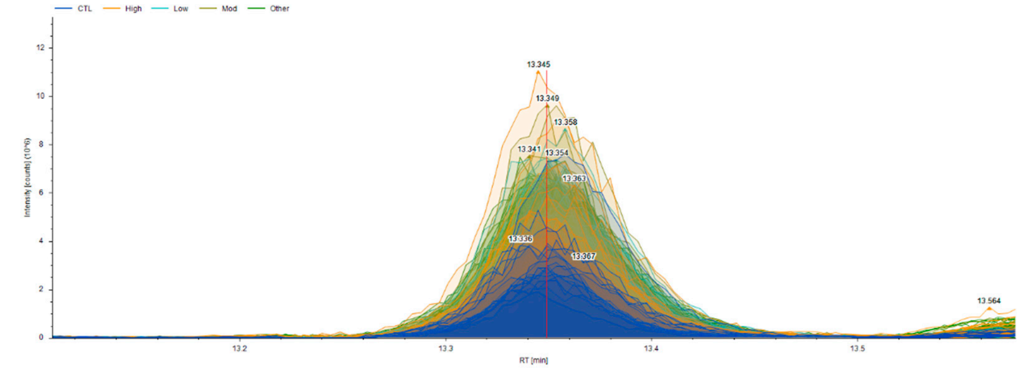	544.3373
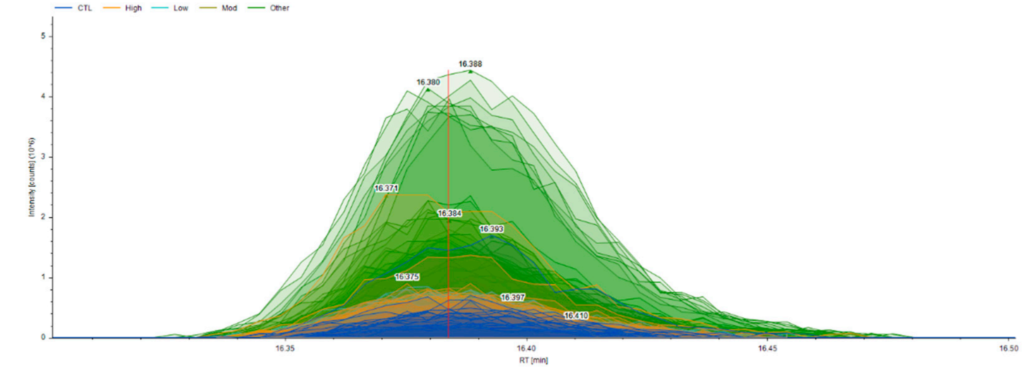	549.1857

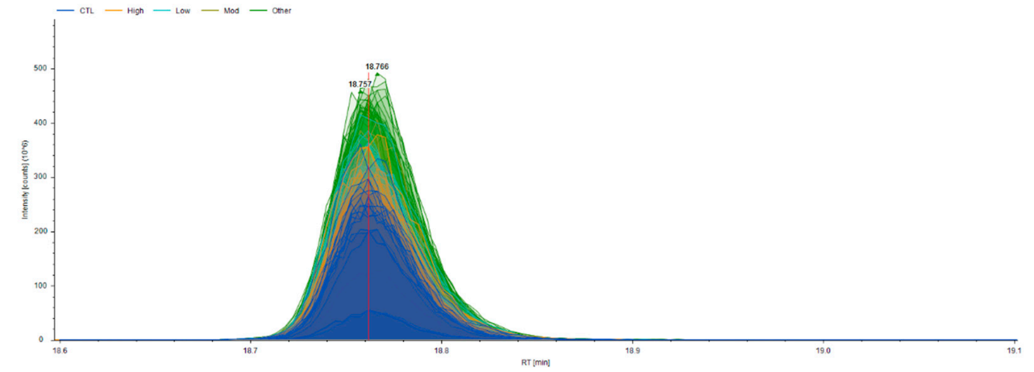

646.2575

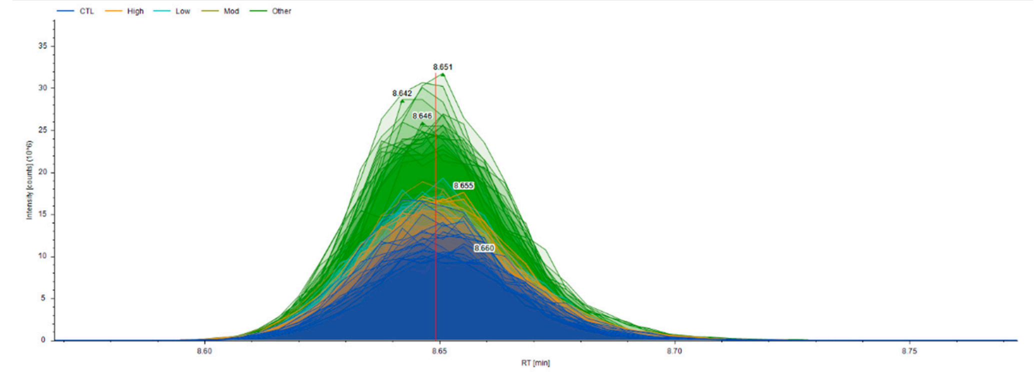

668.5443

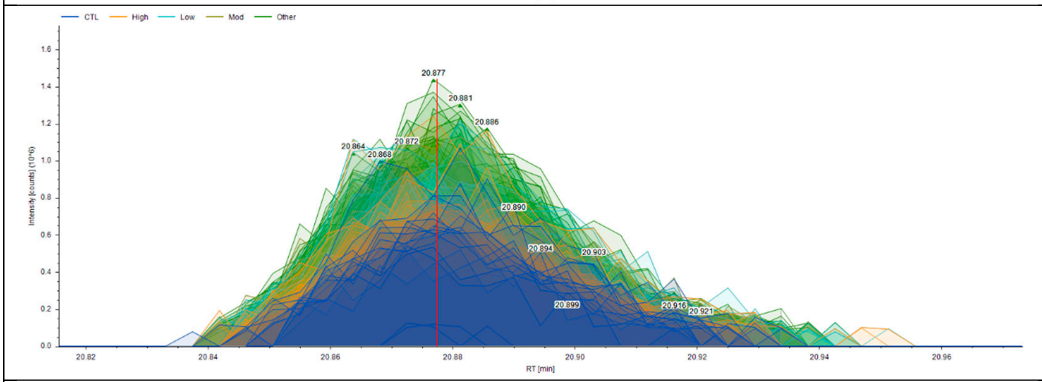

680.3549

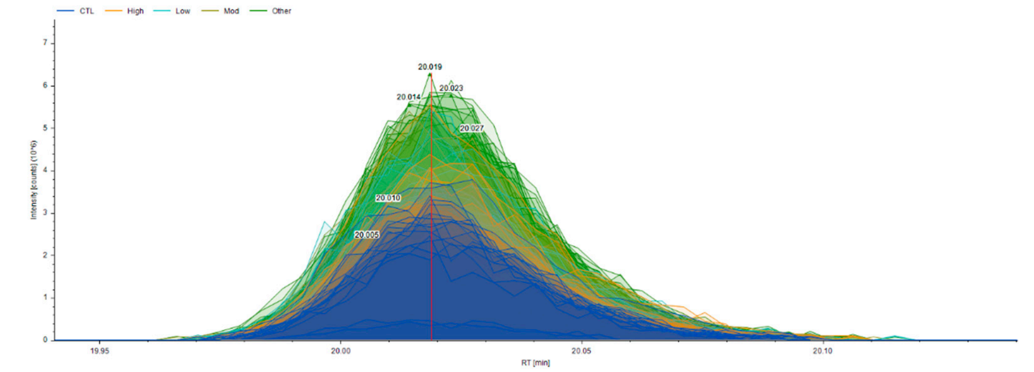

688.3047

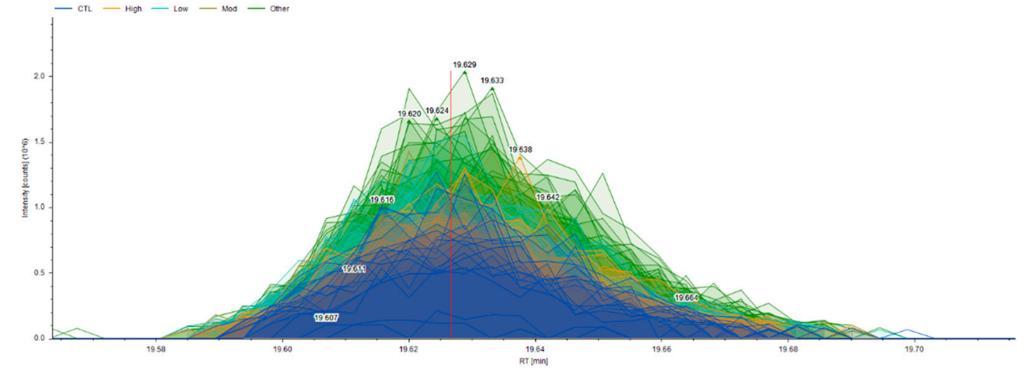	736.2599
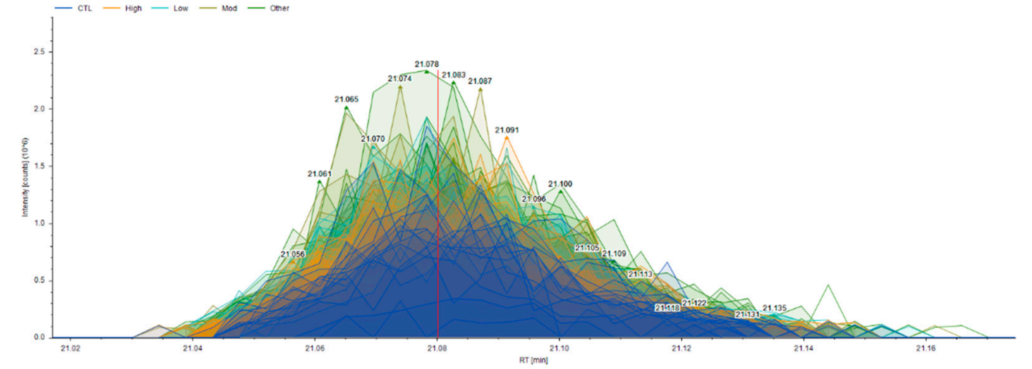	769.4224

Table S12: Demographics information for low PASI classified patients

ID	PASI	Sex	Age (years)	Treatment History	BMI	Psoriasis duration (years)	PsA duration (years)	Comorbidities
797	0.1	FEMALE	60	NSAIDs		27.0	19.0	Hyperlipidemia
3168	0.3	MALE	49	DMARDs	40.9	27.3	14.3	none
3621	0.3	FEMALE	43	NSAIDs	22.0	3.2	3.2	none
3591	0.3	FEMALE	53	NSAIDs		44.3	21.3	Depression + Hyperlipidemia
3475	0.4	MALE	28	DMARDs	26.0	2.0	2.0	Hyperlipidemia
3642	0.4	FEMALE	34	NSAIDs	19.8	16.5	6.5	none
3733	0.5	FEMALE	44	NSAIDs + DMARDs	34.8	13.7	3.7	none
385	0.6	MALE	50	NSAIDs	29.7	30.2	21.2	Hyperlipidemia
3491	0.6	FEMALE	45	NSAIDs	41.0	36.5	2.5	none
3620	0.6	FEMALE	34	NSAIDs + DMARDs	30.1	6.4	1.4	none
268	0.7	FEMALE	62	NSAIDs + DMARDs	27.6	46.8	28.8	Hyperlipidemia
3157	0.8	MALE	46	NSAIDs + DMARDs	28.1	19.9	4.9	none
3064	0.8	FEMALE	58	none	20.6	35.5	9.5	Depression
3273	0.9	MALE	50	NSAIDs	27.1	2.8	2.8	none
3573	0.9	FEMALE	59	none		12.1	4.1	none
3670	0.9	MALE	28	NSAIDs	23.5	15.9	1.9	none
3471	0.9	FEMALE	57	none	26.9	10.5	7.5	none
3079	1	FEMALE	42	none	21.2	34.1	1.1	none
3302	1	MALE	22	DMARDs	31.1	4.0	3.0	none
776	1.2	MALE	41	none	23.5	9.3	4.3	none
3205	1.2	FEMALE	45	NSAIDs		1.4	1.4	Hyperlipidemia
3174	1.2	MALE	18	NSAIDs	16.2	11.1	1.1	none
345	1.2	MALE	47	NSAIDs	24.7	27.2	24.2	none
3169	1.2	FEMALE	49	NSAIDs	25.2	41.7	6.7	none
367	1.2	FEMALE	65	NSAIDs + DMARDs	36.6	27.6	27.6	Hypertension + Hyperlipidemia

693	1.3	FEMALE	42	DMARDs	21.6	21.8	9.8	none
3632	1.3	FEMALE	31	NSAIDs	24.8	6.6	0.6	none
3577	1.5	MALE	60	NSAIDs	23.2	14.5	13.5	none
593	1.5	MALE	75	none	29.7	53.6	18.6	Hypertension + Diabetes + Hyperlipidemia
3438	1.7	MALE	22	none	23.3	0.5	0.5	none
382	1.8	MALE	49	none		23.4	23.4	Hypertension + Diabetes + Hyperlipidemia
417	1.8	MALE	46	NSAIDs	27.9	23.5	23.5	none
3417	1.8	MALE	34	NSAIDs + DMARDs	26.1	2.0	1.0	none
3265	1.8	FEMALE	53	DMARDs + Steroids	49.3	22.9	8.9	Hypertension + Hyperlipidemia
746	1.8	MALE	72	none	30.3	14.9	14.9	none
3649	1.8	MALE	41	NSAIDs	25.6	21.3	1.3	Depression
3613	1.8	MALE	40	DMARDs + Steroids	25.0	20.0	0.0	none
3518	2.1	MALE	49	NSAIDs	26.6	16.9	3.9	none
3507	2.4	FEMALE	34	NSAIDs		0.5	0.5	none
3179	2.6	FEMALE	46	DMARDs	30.4	25.9	2.9	none
574	2.7	FEMALE	68	NSAIDs + DMARDs	21.2	27.6	9.6	none
3412	2.8	FEMALE	44	none	31.6	30.8	10.8	none
3152	3.2	FEMALE	51	none		13.7	0.7	none
3311	3.2	FEMALE	39	NSAIDs	28.7	6.5	5.5	Depression
356	3.3	MALE	65	DMARDs	23.4	27.9	25.9	Hypertension
3661	3.3	MALE	64	NSAIDs + DMARDs	25.8	22.3	22.3	none
3315	3.5	MALE	23	NSAIDs + DMARDs	42.6	8.7	0.7	none
683	3.6	MALE	60	NSAIDs	28.1	31.8	12.8	Hypertension + Hyperlipidemia
3665	3.9	MALE	57	NSAIDs	33.1	12.4	4.4	none
379	4	FEMALE	56	NSAIDs	33.2	30.4	20.4	none
3161	4	MALE	32	NSAIDs		11.0	3.0	none
321	4.1	FEMALE	49	NSAIDs	24.3	38.7	23.7	none
3320	4.2	FEMALE	35	NSAIDs + DMARDs	23.4	14.4	11.4	none

501	4.6	MALE	42	none	31.2	15.4	11.4	Hypertension + Diabetes + Hyperlipidemia
459	4.8	FEMALE	45	NSAIDs + DMARDs	33.3	20.4	15.4	none
3111	4.8	MALE	33	NSAIDs	24.2	22.7	0.7	none

NSAID- non steroidal anti-inflammatory drugs, **DMARD**- disease modifying anti-rheumatic drugs.

Table S13: Demographics information for moderate PASI classified patients

ID	PASI	Sex	Age (yrs)	Treatment History	BMI	Duration of psoriasis (years)	Duration of PsA (years)	Comorbidities
391	5	MALE	60	none	24.3	36.7	34.7	none
700	5.1	MALE	55	NSAIDs	25.9	50.1	23.1	Diabetes + Hyperlipidemia
3380	5.1	MALE	39	DMARDs	22.9	12.5	2.5	Depression
3659	5.1	FEMALE	42	NSAIDs	18.2	16.0	2.0	none
663	5.2	MALE	54	none	28.0	39.0	17.0	none
3321	5.2	MALE	49	NSAIDs	29.0	21.2	1.2	Depression
3313	5.2	MALE	58	DMARDs		33.9	26.9	none
3540	5.3	FEMALE	72	NSAIDs + DMARDs	24.2	44.6	1.6	none
3484	5.3	MALE	41	none	32.9	33.6	0.6	none
3462	5.4	MALE	53	NSAIDs + DMARDs		32.8	10.8	Hyperlipidemia
3482	5.4	FEMALE	54	DMARDs	33.2	33.8	0.8	Hyperlipidemia
332	5.4	MALE	58	NSAIDs	29.9	29.9	28.9	none
3385	5.5	FEMALE	45	none		27.8	1.8	none
619	5.7	MALE	47	NSAIDs	31.9	4.4	4.4	none
1	5.7	MALE	64	NSAIDs	32.8	46.4	31.4	none
486	5.7	MALE	50	NSAIDs + DMARDs		19.2	13.2	none
3266	5.7	FEMALE	31	none	21.8	29.5	3.5	none
3447	5.7	FEMALE	38	NSAIDs	38.1	22.5	0.5	none
3683	5.7	MALE	74	NSAIDs + DMARDs	27.0	25.7	0.7	none
550	5.8	MALE	61	DMARDs	24.9	19.4	21.4	none
3050	6	FEMALE	30	NSAIDs	19.8	6.0	6.0	none
495	6	MALE	47	NSAIDs	22.7	15.2	20.2	Hyperlipidemia
638	6.2	FEMALE	60	none	38.4	31.6	14.6	Depression
3495	6.2	FEMALE	44	NSAIDs + DMARDs	29.2	28.7	15.7	none
3425	6.2	FEMALE	36	NSAIDs	35.6	18.7	10.7	Hyperlipidemia

360	6.3	FEMALE	50	NSAIDs + DMARDs	23.5	20.2	19.2	none
427	6.3	MALE	44	NSAIDs	23.9	21.1	22.1	none
3360	6.4	MALE	54	NSAIDs	52.1	8.2	9.2	none
3439	6.5	MALE	34	NSAIDs + DMARDs	19.8	12.1	3.1	none
3365	6.6	MALE	29	NSAIDs + DMARDs	40.0	4.9	4.9	none
643	6.9	FEMALE	47	NSAIDs + DMARDs	27.1	25.6	6.6	none
3361	7	MALE	56	NSAIDs		30.6	0.6	none
688	7.4	FEMALE	47	NSAIDs + DMARDs	32.4	26.6	16.6	none
3185	7.4	FEMALE	60	DMARDs	27.4	4.3	4.3	none
3527	7.5	FEMALE	19	none	24.6	3.3	3.3	Depression
312	7.5	MALE	45	NSAIDs + DMARDs	27.1	23.1	22.1	none
3434	7.5	MALE	50	NSAIDs		10.5	10.5	none
3444	7.6	FEMALE	47	NSAIDs	35.5	11.4	1.4	Hyperlipidemia
3734	7.8	FEMALE	56	NSAIDs	25.3	31.2	0.2	none
370	8	MALE	39	NSAIDs + DMARDs	30.1	19.7	19.7	none
3080	8.1	FEMALE	62	NSAIDs + DMARDs		37.5	4.5	none
463	8.2	FEMALE	49	NSAIDs + DMARDs + Steroids	28.3	16.9	16.9	none
692	8.4	MALE	48	none	27.1	9.8	5.8	none
3436	8.6	FEMALE	33	none	28.8	10.3	1.3	none
587	8.9	FEMALE	45	DMARDs	25.5	27.1	15.1	none
422	9	FEMALE	54	DMARDs	41.7	28.2	24.2	none
642	9.2	MALE	63	NSAIDs	23.2	16.1	16.1	Hypertension
533	9.2	FEMALE	40	DMARDs		19.1	18.1	Depression
3112	9.2	FEMALE	43	none	28.6	24.7	24.7	none
3274	9.2	MALE	43	none	27.8	28.9	13.9	none
3324	9.4	FEMALE	42	NSAIDs + DMARDs		16.4	0.4	none
3554	9.8	MALE	62	NSAIDs + DMARDs	22.6	3.1	2.1	none
310		FEMALE	67	NSAIDs + DMARDs	38.8	36.8	24.8	Hypertension
79		FEMALE	60	none	22.1	42.1	37.1	none

NSAID- non steroidal anti-inflammatory drugs, **DMARD**- disease modifying anti-rheumatic drugs.

Table S14: Demographics information for high PASI classified patients

ID	PASI	Sex	Age (yrs)	Treatment History	BMI	Duration of psoriasis (years)	Duration of PsA (years)	Comorbidities
3287	10.1	MALE	27	NSAIDs + DMARDs	25.5	14.8	0.8	none
3277	10.2	FEMALE	44	DMARDs	27.4	36.5	22.5	none
3593	10.5	MALE	81	none		16.2	3.2	Hypertension
708	10.7	MALE	49	DMARDs	22.7	19.2	3.2	none
3189	10.8	MALE	46	NSAIDs	27.1	6.8	0.8	none
3487	11.1	MALE	29	none	25.6	19.6	13.6	none
3264	12.2	FEMALE	33	DMARDs + Steroids	28.3	0.4	0.4	none
3336	12.6	MALE	39	NSAIDs + DMARDs	34.1	25.4	0.4	none
3247	12.9	MALE	53	none	33.4	36.2	6.2	Depression
3374	13.3	MALE	51	NSAIDs + DMARDs	26.1	45.2	0.2	none
3407	13.8	FEMALE	56	NSAIDs + DMARDs	37.3	16.5	1.5	Diabetes + Hyperlipidemia
502	14	MALE	40	NSAIDs + DMARDs	28.1	14.5	13.5	none
3303	14.7	FEMALE	27	none		18.2	0.2	none
416	15	MALE	45	NSAIDs	34.3	16.9	14.9	Hypertension
714	16	MALE	50	none	33.0	12.1	2.1	none
3057	16.2	FEMALE	53	DMARDs	36.3	16.9	14.9	none
153	16.2	FEMALE	72	none	25.4	30.7	30.7	Diabetes
681	16.5	MALE	62	none	31.0	35.7	32.7	Hypertension + Hyperlipidemia
597	17.4	MALE	65	DMARDs	27.5	17.2	9.2	none
3368	18	MALE	47	NSAIDs	18.8	8.8	1.8	none
766	18.9	FEMALE	40	none	40.1	15.8	10.8	none

726	19.2	FEMALE	53	DMARDs + Steroids	30.1	36.2	32.2	none
3756	19.3	MALE	23	NSAIDs	21.3	6.3	0.3	none
206	19.5	MALE	59	DMARDs	30.6	37.3	37.3	Hyperlipidemia
3823	20.8	MALE	28	NSAIDs + DMARDs	32.5	8.1	0.1	Depression
3534	21.3	MALE	43	NSAIDs	25.9	14.3	14.3	none
742	22.4	MALE	44	NSAIDs	25.6	18.8	6.8	none
492	24.6	FEMALE	34	NSAIDs + DMARDs	35.3	20.8	16.8	none
3261	26.2	MALE	23	NSAIDs	24.8	5.5	0.0	none
3225	27	MALE	49	NSAIDs	22.6	30.8	0.8	none
3245	28	FEMALE	57	none	24.7	9.8	1.8	Diabetes + Hyperlipidemia
3316	28.2	MALE	41	none		34.8	9.8	none
3190	28.4	MALE	56	none	27.2	6.0	0.0	none
3705	33.3	MALE	65	none	20.3	22.6	19.6	none
3097	37.5	MALE	39	NSAIDs	33.7	22.0	17.0	none
3520	38.4	MALE	54	DMARDs + Steroids	23.9	7.5	7.5	none
3486	38.7	MALE	44	DMARDs	32.9	14.2	1.2	none
3307	40.7	MALE	50	NSAIDs + DMARDs	32.2	10.1	4.1	Hyperlipidemia
623	41.8	FEMALE	51	NSAIDs + DMARDs	36.7	25.7	30.7	none
3194	54.6	FEMALE	57	none	19.1	5.6	5.6	none

Dur. PS: duration of psoriasis (in years). **Dur. PsA**: duration of psoriatic arthritis (in years). **NSAID**- non steroidal anti-inflammatory drugs, **DMARD**- disease modifying anti-rheumatic drugs.
